# In vitro and ex vivo anti-myeloma effects of nanocomposite As_4_S_4_/ZnS/Fe_3_O_4_

**DOI:** 10.1038/s41598-022-22672-5

**Published:** 2022-10-26

**Authors:** Danka Cholujova, Lenka Koklesova, Zdenka Lukacova Bujnakova, Erika Dutkova, Zuzana Valuskova, Patricia Beblava, Anna Matisova, Jan Sedlak, Jana Jakubikova

**Affiliations:** 1 Department of Tumor Immunology, Biomedical Research Center, Cancer Research Institute, Slovak Academy of Sciences, Dubravska Cesta 9, Bratislava, 84505 Slovakia; 2 Centre for Advanced Materials Application, Slovak Academy of Sciences, Dubravska Cesta 9, Bratislava, 84511 Slovakia; 3 Department of Obstetrics and Gynecology, Jessenius Faculty of Medicine, Comenius University in Bratislava, Martin, 03601 Slovakia; 4 Department of Mechanochemistry, Institute of Geotechnics, Slovak Academy of Sciences, Watsonova 45, Košice, 04001 Slovakia

**Keywords:** Cancer, Cell biology, Nanoscience and technology

## Abstract

Nanoparticles in medicine can integrate actively targeted imaging agents and drug delivery vehicles, and combining multiple types of therapeutics in a single particle has numerous advantages, especially in multiple myeloma. MM is an incurable hematological disorder characterized by clonal proliferation of plasma cells in the bone marrow. In this study, we evaluated the anti-myeloma activity of 3 nanocomposites (3NPs): As_4_S_4_/ZnS/Fe_3_O_4_ (1:4:1), As_4_S_4_/ZnS/Fe_3_O_4_ with folic acid (FA), and As_4_S_4_/ZnS/Fe_3_O_4_ with FA and albumin with reduced survival MM cell lines and primary MM samples by each of 3NP. Cytotoxic effects of 3NPs were associated with caspase- and mitochondria-dependent apoptosis induction and reduced c-Myc expression. Modulation of cell cycle regulators, such as p-ATM/ATM and p-ATR/ATR, and increases in p-Chk2, cyclin B1, and histones were accompanied by G_2_/M arrest triggered by 3NPs. In addition, 3NPs activated several myeloma-related signaling, including JNK1/2/3, ERK1/2 and mTOR. To overcome BM microenvironment-mediated drug resistance, nanocomposites retained its anti-MM activity in the presence of stroma. 3NPs significantly decreased the stem cell-like side population in MM cells, even in the context of stroma. We observed strong synergistic effects of 3NPs combined with lenalidomide, pomalidomide, or melphalan, suggesting the potential of these combinations for future clinical studies.

## Introduction

Multiple myeloma (MM) is an incurable hematological malignancy that is characterized by uncontrolled proliferation of clonal plasma cells within the bone marrow (BM), high levels of monoclonal protein in serum and/or urine, and BM plasmacytosis associated with organ dysfunction^1^. The pathogenesis of MM is a result of a number of primary genetic abnormalities, including chromosomal translocations involving immunoglobulin heavy-chain genes and aneuploidy, as well as secondary genetic alterations such as copy-number variants, oncogenic mutations, and epigenetic alterations^2^. In addition to genetic anomalies intrinsic to MM clones, the tumor microenvironment plays a central role in myelomagenesis, mediating resistance to cell death and promoting sustained proliferation, cell homing and invasion, thereby contributing to MM progression^3^. Ongoing studies continue to characterize the deregulation of these genetic aberrations and the intra-clonal heterogeneity leading to MM transformation in the BM microenvironment. The BM biopsy sample and blood biomarker assays used to confirm an MM diagnosis and assess MM stage fail to account for spatial heterogeneity; however, clinical diagnostic imaging modalities, such as PET, CT, and MRI, have been successfully utilized to monitor MM activity in all bodily locations within patients^4,5^. Before developing clinical symptoms, MM is preceded by premalignant precursor conditions known as monoclonal gammopathy of undetermined significance (MGUS) and smoldering (asymptomatic) MM (SMM), which do not require therapy^6^. Immunomodulatory drugs, proteasome inhibitors, histone deacetylate inhibitors, and monoclonal antibodies are currently used to treat patients with symptomatic MM^7^. Despite advances in current MM treatment options, the disease eventually relapses and drug resistance develops, leading to patient death due to unmitigated disease progression.

The use of nanoparticles in medicine, called nanomedicine, can combine various therapeutic agents with different mechanisms of action into a single carrier to augment drug delivery and/or facilitate longitudinal imaging to monitor disease progression and tumor responses to treatment, thereby targeting only tumor cells and minimizing off-target toxicity^8^. Compared to small molecules or antibody conjugates, nanoparticles show higher surface area-to-volume ratios, which enhance drug delivery or molecular imaging agent incorporation, and/or promote controlled release. Therapeutic and diagnostic nanosuspensions as well as theranostic agents, simultaneously capable of molecular imaging and drug delivery with one vehicle, have unique chemical and physical constituents that show high biocompatibility, prolonged circulation time, the ability to dissolve a broad variety of pharmaceuticals with low solubility, augmentation of tissue-specific delivery, and/or stimuli-responsive interactions with biological components^9,10^. Nanoparticles (NPs), typically between 5 and 200 nm in size and derived from either organic- or inorganic-based NPs, can deliver therapeutic or imaging moieties by passive, active or triggered targeting. In preclinical in vivo MM studies, tumor burden has shown significant reduction with less toxicity, compared to the effect of free drugs, with liposomal bortezomib and carfilzomib with or without liposomal doxorubicin treatment^11–13^. Nanoparticles encapsulating small-molecule chemotherapeutics, including albumin-based protein-drug conjugates formulated with paclitaxel (Abraxane; NCT01646762) and liposomal formulations of doxorubicin (Doxil and Myocet), are FDA-approved nanomedicine-based treatments for MM. However, similar to single agents, combinations of Abraxane with lenalidomide (NCT02075021) and Doxil with bortezomib^14^ have not improved outcomes in the treatment of relapsed or refractory MM patients. Despite advances, several challenges, such as the ability to combine molecularly targeted agents with various mechanisms of action and longitudinal imaging of MM clonality while minimizing off-target toxicity, need to be addressed via the use of nanomedicines.

The nanocomposite As_4_S_4_/ZnS/Fe_3_O_4_ demonstrating therapeutic, magnetic and optical functionality has been evaluated in terms of its particle size distribution, zeta potential, and long-term stability^15^. Previous studies have shown strong in vitro and in vivo anti-cancer effects of As4S4 nanoparticles (realgar) in MM, leukemia and melanoma^16–18^. In this study, we evaluated the concentration- and time-dependent cytotoxicity of nanosuspensions comprising 3 composite nanoparticles (3NPs): As_4_S_4_/ZnS/Fe_3_O_4_ (1:4:1), As_4_S_4_/ZnS/Fe_3_O_4_ (1:4:1) with folic acid (FA), and As_4_S_4_/ZnS/Fe_3_O_4_ (1:4:1) with FA and albumin (Alb) against several MM cell lines in vitro and ex vivo isolated PC, without significant toxicity toward normal cells, with a higher anti-MM activity observed by As_4_S_4_/ZnS/Fe_3_O_4_ with FA and Alb. To assess the resistance of the stromal compartment in the MM microenvironment, the effects of composite 3NPs were determined. Each 3NP type inhibited MM cell proliferation and showed anti-MM activity even in the context of the stromal cells. Evaluation of combinations of either novel or conventional anti-MM agents with 3NPs revealed strong synergistic effects with lenalidomide, pomalidomide, or melphalan, suggesting that these combinations have clear potential for use in future MM clinical studies. In particular, we showed a significant reduction in the proportion of a myeloma-initiating stem-like side population (SP) after treatment by 3NPs in MM, even in the context of the BM stromal cells. Cellular and molecular mechanisms of action by composite 3NPs included early activation of signaling molecules (including p-ERK1/2, p-JNK, p-mTOR, and histones p-H3/H3 and p-H2AX/H2AX), and these effects were accompanied by G_2_/M block of the cell cycle via modulation of cell cycle regulatory molecule levels (including upregulation of Cyclin B1, p-chk2, p-ATR, and downregulation of p-ATM/ATM, ATR, chk1, and chk2) as well as induction of apoptosis with modulation of apoptotic-signaling molecules (caspase- and mitochondria-dependent) associated with downregulation of c-Myc, suggesting that 3NPs are strong potential therapeutic candidates for the treatment of MM.

## Materials and methods

### Reagents

Nanosuspensions of 3NPs: As_4_S_4_/ZnS/Fe_3_O_4_, As_4_S_4_/ZnS/Fe_3_O_4_ with FA, and As_4_S_4_/ZnS/Fe_3_O_4_ with FA and Alb were prepared by wet stirred media milling in a laboratory circulation mill MiniCer (Netzsch, Germany). The samples were prepared in 1:4:1 molar ratio between As_4_S_4_, ZnS and Fe_3_O_4_. Three grams of mechanochemically synthesized As_4_S_4_/ZnS/Fe_3_O_4_ nanocomposite samples were subjected to milling in the presence of 300 ml of Poloxamer 407 (PX407) water solution (0.5 wt%), 300 ml of PX407 water solution (0.5 wt%) containing FA water solution (0.1 wt%) or 300 ml of PX407 water solution (0.5 wt%) containing FA and Alb water solution (0.1 wt%) for 60 min at a milling speed of 3500 rpm. The mill was loaded with yttrium-stabilized ZrO2 milling balls (diameter 0.6 mm). The resulting nanoparticle suspensions were centrifuged at 3000 rpm, filtered through a 0.22 μm sterile filter, and then stored at 4 °C. Basic characterization of composite nanoparticle suspensions were measured by photon cross-correlation spectroscopy using a Nanophox particle size analyzer (Sympatec, Germany) to evaluate distribution of particle size; a Zetasizer Nano ZS (Malvern, Great Britain) to measure the electrophoretic mobility of the particles, which is converted into the zeta potential by using the Smoluchowski equation built into the Malvern zetasizer software; magnetic properties of NPs tested in magnetic field up to 0.3 T using the VSM M-555 (PARC, USA) magnetometer; and a Tensor 29 infrared spectrometer (Bruker, Germany) using a ATR method to record the Fourier transform infrared spectroscopy (FTIR) spectra measurements^15^. In addition, optical absorption spectra were detected using a UV–Vis spectrophotometer Helios Gamma (Thermo Electron Corporation, Great britan), photoluminescence spectra by spectrofluorometer PC1 (ISS, USA) and fluorescence by Leica DM6000B with excitation filter 450–490 nm^15^. Bortezomib (BTZ; Velcade), lenalidomide (LEN; CC5013) and pomalidomide (POM; CC4043) were obtained from Selleck Chemicals (Houston, TX, USA). Doxorubicin (DOX); dexamethasone (DEX), and melphalan (MEL) were obtained from Sigma-Aldrich.

### Primary cells and cell lines

Multiple myeloma (MM) cell lines MM.1S and U266 were obtained from the ATCC (American Type Culture Collection, Manassas, VA) and OPM-2 and L-363 cells were obtained from the DSMZ (Deutsche Sammlung von Mikroorganismen und Zellkulturen GmbH, Braunschweig, Germany). The chemosensitive cell line RPMI 8226-S and its sublines resistant to doxorubicin (RPMI-Dox40), mitoxantrone (RPMI-MR20), and melphalan (RPMI-LR5) were kindly provided by Dr. William S. Dalton (Lee Moffitt Cancer Center, Tampa, FL, USA). Human MM cell lines OPM-1, KMS-11, and KMS-34 were kindly donated by Dr. Teru Hideshima (Dana Farber Cancer Institute, Boston, MA, USA). Adenocarcinoma Caco-2 cells, breast cancer MCF-7 cell line and the human bone marrow stromal cell line HS-5 were obtained the ATCC (American Type Culture Collection, Manassas, VA). Acute promyelocytic leukemia cell lines: HL60, HL60-MDR1, and HL60/PLB-ABCG2 cells were gifted from Dr. Jendzelovsky. All MM and leukemia cell lines were cultured in RPMI 1640 medium (Cellgro, Mediatech, VA) supplemented with 10% heat-inactivated fetal bovine serum (FBS; Harlan, Indianapolis, IN), 100 u/ml penicillin, 100 μg/ml streptomycin and 2 mM L-glutamine (GIBCO, Grand Island, NY) at 37 °C in 5% CO_2_. In addition, Caco-2, MCF-7 cells as well as stromal cell line HS-5 were cultured in Dulbecco’s modified Eagle medium (DMEM; Cellgro, Mediatech, VA) supplemented with 10% heat-inactivated fetal bovine serum (FBS; Harlan, Indianapolis, IN), 100 u/ml penicillin, 100 μg/ml streptomycin and 2 mM L-glutamine (GIBCO, Grand Island, NY) at 37 °C in 5% CO_2_.

Fresh mononuclear cells (MNCs) were obtained from patients and healthy volunteers by Ficoll-Hypaque (Pharmacia, Piscataway, NJ, USA) density sedimentation. Patient MM cells were purified by cell sorting using CD138-PE monoclonal antibody to isolate CD138 + PC cells (MM cells) and tumor microenvironment (accessory) cells (non-PC cells) from freshly obtained BM of the same MM patient. Cells were cultured in RPMI 1640 medium containing 20% heat-inactivated FBS, 100 u/ml penicillin, 100 μg/ml streptomycin and 2 mM L-glutamine, and then maintained at 37 °C in 5% CO_2_. Approval for this study was obtained from the Biomedical Research Center Institutional Review Board under the protocol Myelom 001. Informed consent was obtained from all patients and healthy volunteers, in accordance with the Declaration of Helsinki protocol.

### Composite nanoparticles sensitivity of cell-based assays

The effect of composite nanoparticles (NPs) on MM cells was evaluated using the following assays: viability assessment with colorimetric survival 3-[4,5-dimethylthiazol-2-yl]-2,5-diphenyltetrazolium bromide (MTT) assay and luminescent CellTiterGlo (CTG) assay; annexin V-fluorescein isothiocyanate assay for quantification of apoptosis; assessment of mitochondrial membrane potential by flow cytometric analysis using the JC-1 fluorescent probe; flow cytometric analysis of DNA content of nuclei labeled with propidium iodide (PI) for evaluation of cell cycle changes; assessment of cell division of MM cells by carboxyfluorescein diacetate succinimidyl ester (CFSE) staining as well as to distinguish between CFSE-labeled MM cells and unlabeled HS-5 stromal cells; and determination of non-viable (dead) cells by PI staining. Detailed information on these assays is included in the Supplemental Methods.

### Molecular profiling analyses and functional assays

The molecular sequelae of composite nanoparticles (NPs) treatment in MM cells were evaluated by Western immunoblotting analyses, as described in Supplemental Methods. The functional Hoechst 33,342 assay was used to assess impact of 3 composite nanoparticles (3NPs) on the stem cell-like SP, either alone or in co-culture model with BMSC HS-5 cells, by labeling of MM cells with carboxyfluorescein diacetate succinimidyl ester (CFSE) and non-viable (dead) cells by 7-AAD staining, according to manufacturer’s instructions and detailed in the Supplemental Methods.

### Statistical analysis

The statistical significance of differences in composite nanoparticles (NPs)-treated versus control samples was determined using Student’s t test. Data are presented as mean ± standard deviation. The minimal level of significance was *p* < 0.05. The half maximal effective concentration (EC50) of 3 composite nanoparticles (3NPs) was evaluated using CalcuSyn software (Biosoft, Ferguson, MO, USA).

## Results

### Cytotoxic effects of composite NPs in MM cell lines

**Figure 1:**
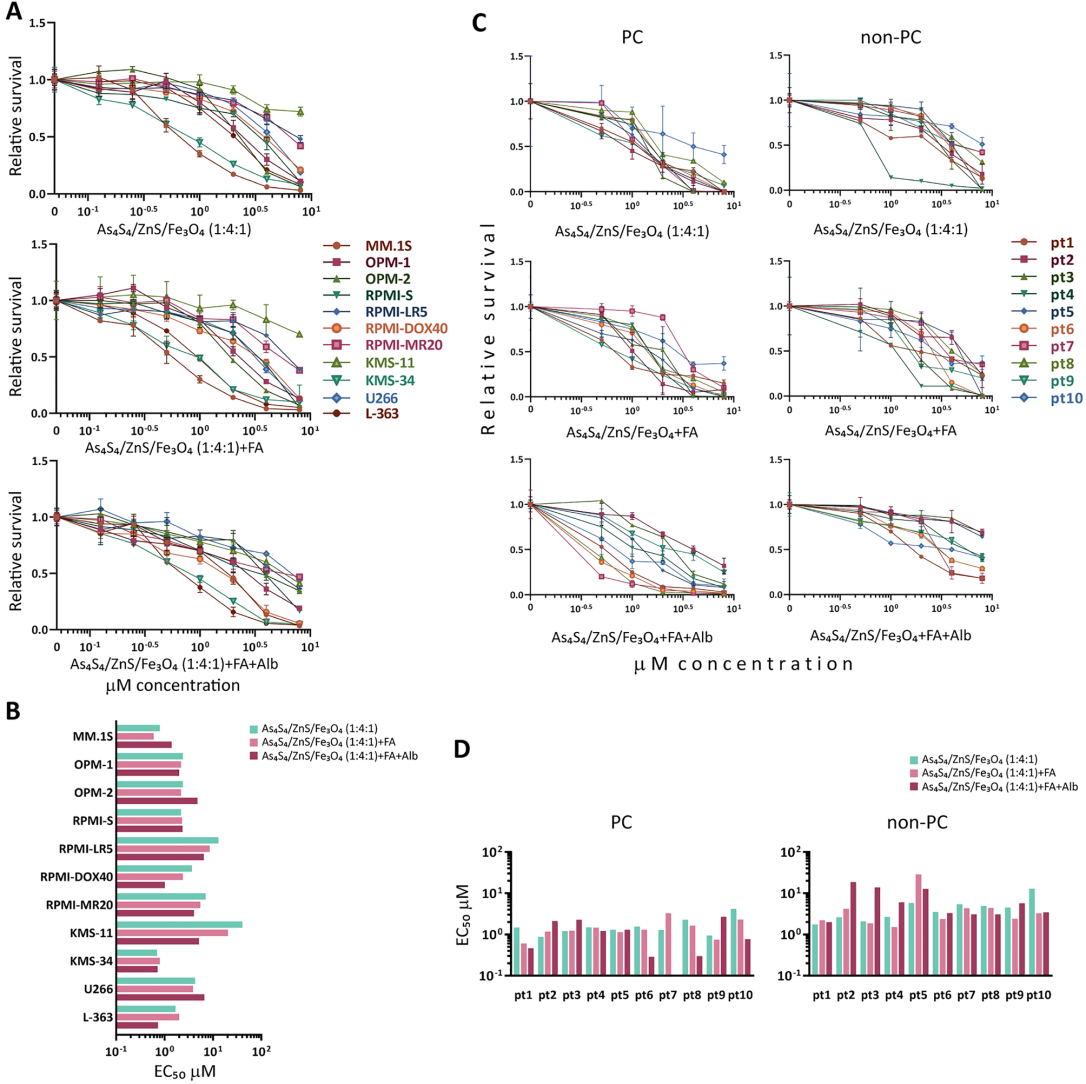
Cytotoxic effects of As_4_S_4_/ZnS/Fe_3_O_4_ (1:4:1), As_4_S_4_/ZnS/Fe_3_O_4_ (1:4:1) with FA, and As_4_S_4_/ZnS/Fe_3_O_4_ (1:4:1) with FA and Alb in MM cells. (**A**) MM cell lines (MM.1S, OPM-1, OPM-2, RPMI-S, RPMI-LR5, RPMI-DOX40, RPMI-MR20, KMS-11, KMS-34, U266, and L-363) were treated with all 3NPs in the concentrations: 0.125, 0.25, 0.5, 1, 2, 4, and 8 µM for 48 h and cell survival was analyzed by MTT assay. (**B**) The EC_50_ values of all 3NPs were determined in MM cells for 48 h by the CalcuSyn software. (**C**) Freshly sorted bone marrow CD138 + PC from MM patients (pt; N = 10) and its tumor microenvironment cells (non-PC; N = 10) were cultured with 3NPs (0–8 µM) for 48 h. Relative survival (fold change to control) was assessed using a CellTiterGlo assay. (**D**) The EC_50_ values of all 3NPs in PC and non-PC populations were determined for 48 h by the CalcuSyn software. Each treatment with a specific concentration of NPs was done in triplicate. The data presented are mean ± standard deviation, expressed as survival/viability relative to untreated controls.

To assess the anti-cancer activities of 3 composite nanoparticles (3NPs): As_4_S_4_/ZnS/Fe_3_O_4_ (1:4:1), As_4_S_4_/ZnS/Fe_3_O_4_ (1:4:1) with FA, and As_4_S_4_/ZnS/Fe_3_O_4_ (1:4:1) with FA and Alb in MM, the cytotoxic effects of 3NPs against MM cell lines were examined. We treated 11 MM cell lines (MM.1S, OPM-1, OPM-2, RPMI-S, RPMI-LR5, RPMI-DOX40, RPMI-MR20, KMS-11, KMS-34, U266, and L-363 cell lines) with different concentrations of each 3NP type in the range of 0–8 μM for 48 h and evaluated cell survival by MTT assay. All 3NPs significantly decreased cell viability for the whole panel of MM cell lines in a concentration-dependent manner (Fig. 1A). A concentration-dependent reduction in cell viability was determined by EC_50_ value (the concentration at which 50% of cells survive) for all 3NPs using CalcuSyn software. Sensitive MM cells, including MM.1S, KMS-34, and L-363 cells, exhibited EC_50_ values < 2 μM, whereas the majority of MM cells had an EC_50_ in the range of 2–40.7 μM, with the highest resistance observed in KMS-11 and RPMI-LR5 cells exposed to As_4_S_4_/ZnS/Fe_3_O_4_ (1:4:1) (Fig. 1B). Similarly, we observed a significant decrease in MM cell viability at 24 h and 72 h after treatment with each 3NP type in a concentration- and time-dependent manner, with the strongest activity determined for As_4_S_4_/ZnS/Fe_3_O_4_ (1:4:1) with FA and Alb, by MTT viability assay (Suppl. Fig. S1A). The EC_50_ values of As_4_S_4_/ZnS/Fe_3_O_4_ (1:4:1) with FA and Alb were 2–5 times lower than those of As_4_S_4_/ZnS/Fe_3_O_4_ (1:4:1) with/without FA at 72 h in most MM cell lines (Suppl. Fig. S1B). Moreover, we determined the cytotoxic effects of 3NPs on acute promyelocytic leukemia cell lines (HL60, HL60-MDR1, and HL60/PLB-ABCG2 cell lines) and adenocarcinoma Caco-2 and breast cancer MCF-7 cell lines (Suppl. Fig. S2A), with the strongest effects found in HL60 cells with EC_50_ values of 1.1–3.6 μM for As_4_S_4_/ZnS/Fe_3_O_4_ (1:4:1) with/without FA at all time points (Suppl. Fig. S2B).

Next, we extended the evaluation of the antiproliferative effects of 3NPs to a panel of CD138^+^ PC and non-PC of the tumor microenvironment; all cells were derived from the BM of different MM patients (N = 10) (Fig. 1C). All 3NPs displayed high one-digit micromolar-level of anti-MM activity against all 10 CD138^+^ PC derived from MM patients, with the strongest effect exerted by As_4_S_4_/ZnS/Fe_3_O_4_ (1:4:1) with FA and Alb, for which the EC_50_ was on average 3–sixfold lower than that of non-PC of the same patients (Fig. 1D). In addition, we determined the effects of 3NPs on healthy peripheral blood mononuclear cells isolated from 13 normal donors (MNCs; N = 13) (Suppl. Fig. S3A). Healthy MNCs were significantly more resistant than MM tumor cells, MM cell lines or primary patient-derived PC. The most effective EC_50_ values were obtained for As_4_S_4_/ZnS/Fe_3_O_4_ (1:4:1) with FA and Alb; they were on average threefold higher in healthy MNCs than in non-PC cells and were on average 19-fold higher than those in CD138^+^ PC population derived from MM patients (Suppl. Fig. S3B). Our results confirmed that 3NPs, with the highest anti-tumor activity shown by As_4_S_4_/ZnS/Fe_3_O_4_ (1:4:1) with FA and Alb, specifically decreased the survival of MM cells (both MM cell lines and primary patient-derived PC/MM cells) while inducing significantly less cytotoxicity on non-PC and healthy cells.

### Apoptosis was increased with 3NPs in MM cell lines

**Figure 2:**
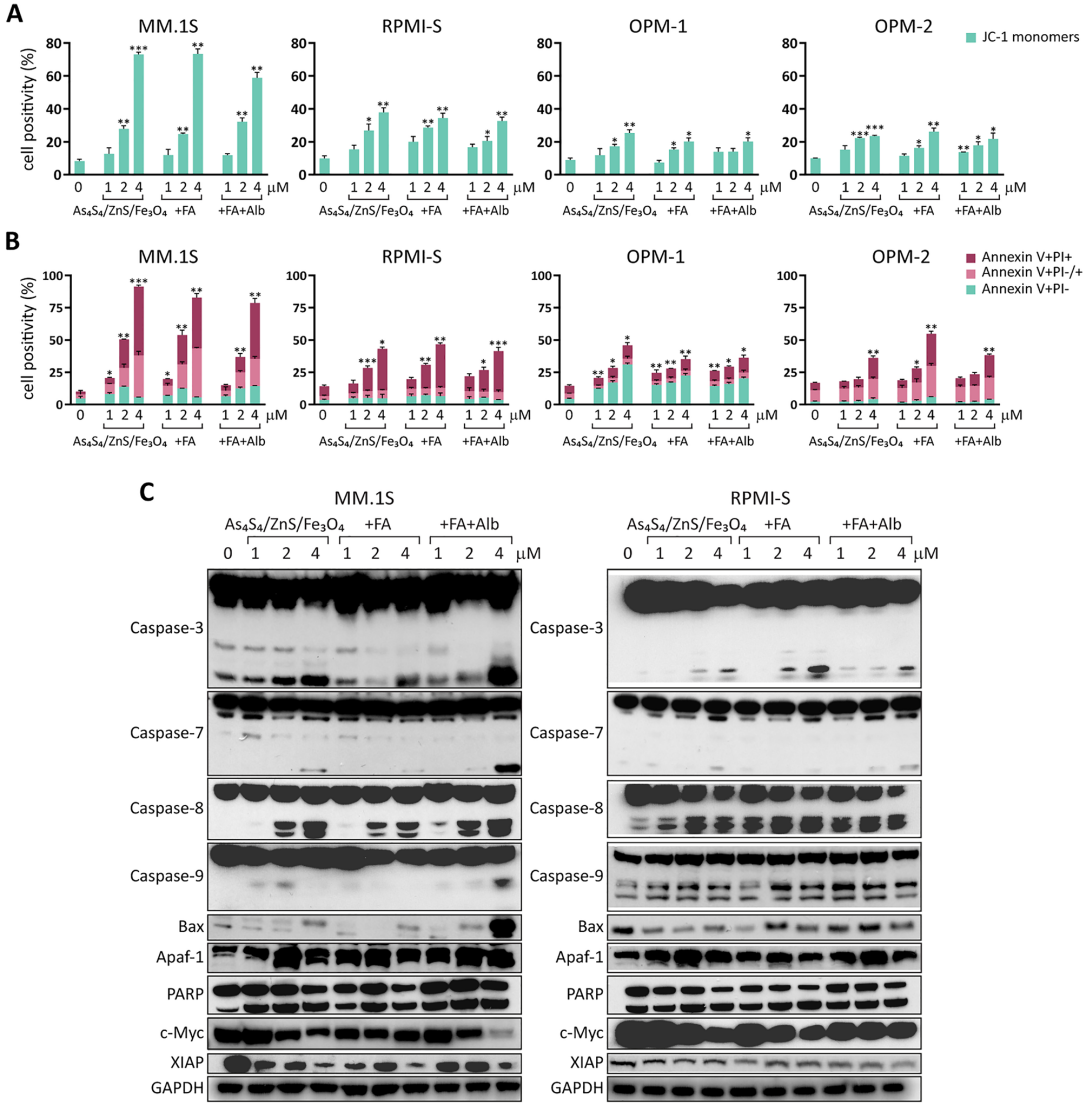
As_4_S_4_/ZnS/Fe_3_O_4_ (1:4:1), As_4_S_4_/ZnS/Fe_3_O_4_ (1:4:1) with FA, and As_4_S_4_/ZnS/Fe_3_O_4_ (1:4:1) with FA and Alb trigger apoptosis and induce apoptosis-associated signaling in MM cells. MM.1S, RPMI-S, OPM-1, and OPM-2 cells were cultured with all 3NPs at 1, 2, and 4 µM for 48 h. (**A**) Depletion of mitochondrial membrane potential in 3NPs-treated MM cell lines was quantified by straining with the fluorescent JC1 dye by increased levels of JC1 monomers, and analyzed by a FACS Canto II flow cytometer. Data are from three independent experiments. (**B**) Effects of all 3NPs on induction of apoptosis and necrosis were evaluated with Annexin V-FITC and PI staining. Percentages of early apoptotic (Annexin V-FITC +/PI −), late apoptotic (Annexin V +/PI +/−) and necrotic (Annexin V +/PI+) cells were analyzed by a FACS Canto II flow cytometer. Data are from two independent experiments, presented as mean ± standard deviation. Significant differences between treatments and control were identified by t-test with *p < 0.05, **p < 0.01, and ***p < 0.001. (**C**) MM.1S and RPMI-S cells were cultured with all 3NPs at 1, 2, and 4 µM for 48 h. Whole cell lysates (20 μg of proteins/lane) were subjected to western blot analyses using anti-caspase-3, -caspase-7, -caspase-8, -caspase-9, -Bax, -Apaf-1 (apoptotic protease activating factor-1), -PARP, -c-Myc, and -XIAP (x-linked inhibitor of apoptosis) antibodies. GAPDH served as a loading control. Results are representative of two independent experiments.

To address whether the cytotoxic effect of 3NPs directly correlates with the induction of apoptosis, cell damage was estimated by the loss of mitochondrial membrane potential (MMP), a hallmark of apoptosis. Aggregation of JC-1 dimers in viable cells and their disassociation in dying cells results in the production of JC-1 monomers that are directly correlated with a decrease in MMP. Therefore, the percentage of JC-1 monomers was determined to delineate 3NPs-induced MMP alterations in 4 MM cell lines (MM.1S, RPMI-S, OPM-1, and OPM-2 cells) (Fig. 2A). We observed a significant decrease in MMP, as characterized by a higher percentage of JC-1 monomers in MM cell lines; the decrease followed a dose-dependent pattern (2 and 4 μM) and was more pronounced in MM.1S and RPMI-S cells compared to OPM-1 and OPM-2 cells exposed to all 3NPs at 48 h. Similar observations in the disruption of MMP were detected at 24 h (Suppl. Fig. S4A), with no significant differences among all 3 NPs observed.

To confirm the apoptotic effects of composite 3NPs, we evaluated early apoptotic events associated with transmembrane phosphatidylserine externalization, as determined by Annexin V staining, in all composite nanoparticle-treated MM cells (MM.1S, RPMI-S, OPM-1, and OPM-2 cells) at different treatment concentrations (1, 2, and 4 μM) and durations (24, 48, and 72 h) (Fig. 2B and Suppl. Fig. S4B). Late necrotic events are accompanied by PI internalized into the nucleus of dead cells. All 3NPs induced significant apoptosis in the following cells: MM.1S > RPMI-S > OPM-2 = OPM-1 cells in a concentration-dependent manner at 48 h. Early apoptosis is represented by a higher percentage of Annexin V +/PI− cells primarily in OPM-1 cells treated with 3NPs and late apoptosis, which is indicated by Annexin V +/PI +/− cells, showed the highest proportion in MM.1S and OPM-2 cells treated with 3NPs. An increase in the percentage of necrosis is evidenced by the proportion of Annexin V +/PI+ cells, and the highest percentage of these cells was observed in MM.1S and RPMI-S cells (Fig. 2B). Similar proportions of apoptotic/necrotic cells were observed at 24 h of 3NP treatment (Suppl. Fig. S4B). Moreover, no significant differences in the induction of apoptosis were revealed among 3NPs.

Furthermore, a western immunoblot analysis was performed to examine the molecular mechanism of apoptosis (Fig. 2C). Exposure to 3NPs for 48 h triggered cleavage of caspase-3, -7, -8, -9 and PARP, with a increase in the pro-apoptotic protein Apaf-1 level. In addition, the pro-apoptotic protein Bax was only upregulated in MM.1S. In addition, X-linked inhibitor of apoptosis XIAP was downregulated in both MM.1S and RPMI-S cells. Importantly, we observed that treatment with all 3NPs markedly downregulated c-Myc in a concentration-dependent manner. Taken together, our data indicate that a significant decrease in cell survival was associated with the induction of apoptosis, disruption of mitochondrial membrane potential, activation of caspases and modulation in the levels of pro- and anti-apoptotic factors.

### G_2_/M block of the cell cycle induced by 3NPs in MM cell lines

**Figure 3:**
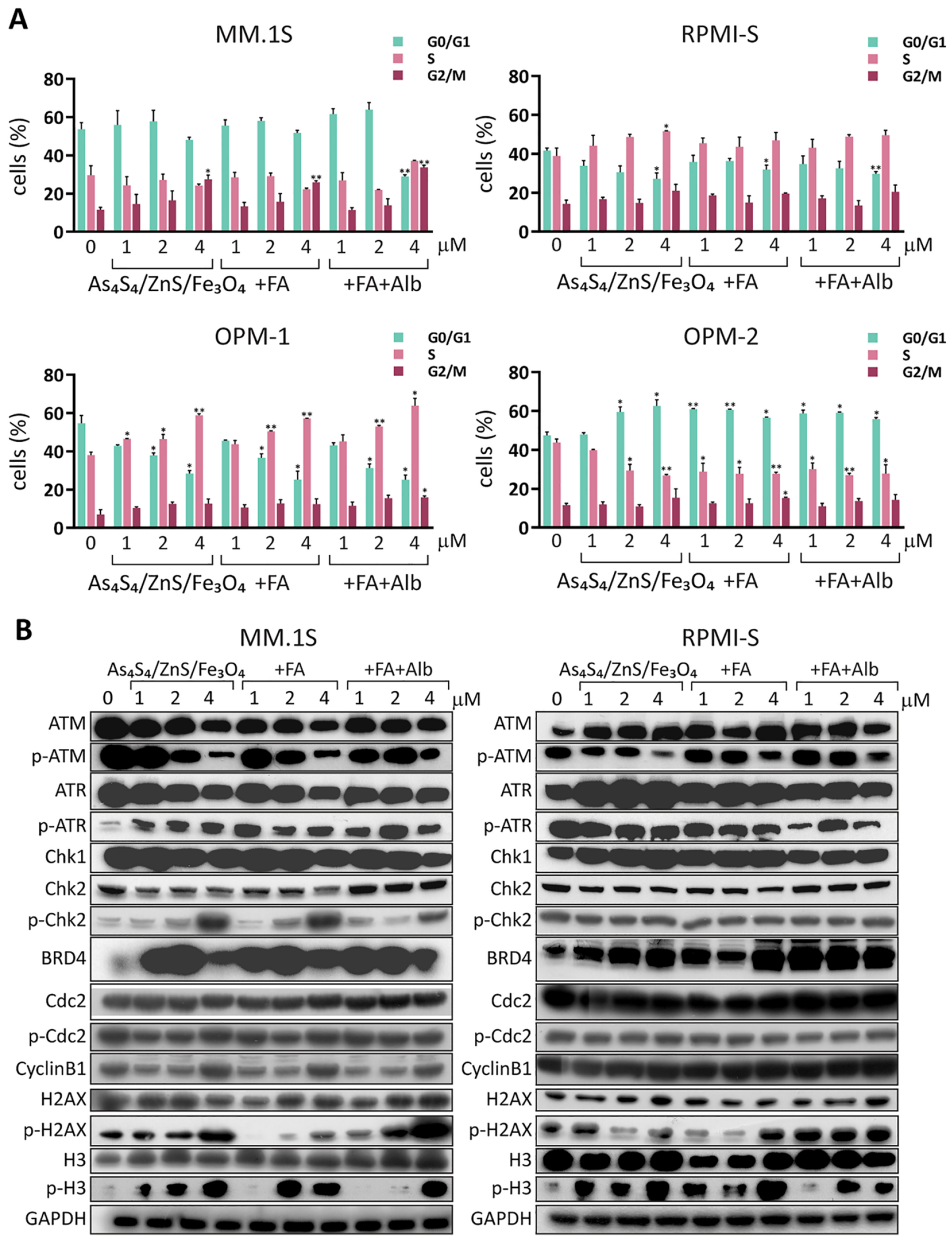
As_4_S_4_/ZnS/Fe_3_O_4_ (1:4:1), As_4_S_4_/ZnS/Fe_3_O_4_ (1:4:1) with FA, and As_4_S_4_/ZnS/Fe_3_O_4_ (1:4:1) with FA and Alb trigger cell cycle arrest and induce cell cycle-associated signaling in MM cells. (**A**) MM.1S, RPMI-S, OPM-1, and OPM-2 cells were cultured with all 3NPs at 1, 2, and 4 µM for 24 h. The distribution of cells in G_0_/G_1_, S, and G_2_/M phase were measured by a FACS Canto II flow cytometer and analyzed by De Novo FCS Express software. Data are representative of three independent experiments. (**B**) MM.1S and RPMI-S cells were cultured with all 3NPs at 1, 2, and 4 µM for 24 h. Whole cell lysates (20 μg of proteins/lane) were immunoblotted using anti-ATM, -p-ATM, -ATR, -p-ATR, -Chk1, -Chk2, -p-Chk2, -Cdc-2, -p-Cdc-2, -Cyclin B1, -histone H2AX (H2AX), -p-histone H2AX (p-H2AX), -histone H3 (H3), -p-histone H3 (p-H3), and -GAPDH (used as a loading control) antibodies. Results are representative of three independent experiments, presented as mean ± standard deviation. Significant differences between treatments and control were identified by t-test with *p < 0.05 and **p < 0.01.

To analyze the cellular mechanisms triggered by NPs in MM cell lines, we examined the cell cycle profile of MM cell lines (MM.1S, RPMI-S, OPM-1, and OPM-2 cells) treated with 3NPs (1, 2, and 4 μM) for 24 and 48 h using flow cytometry (Fig. 3 and Suppl. Fig. S5). We observed a significant increase in the proportion of MM.1S cells in the G_2_/M phase at the highest concentration (4 µM) at 24 and 48 h. The highest increase in the G_2_/M phase was induced by As_4_S_4_/ZnS/Fe_3_O_4_ (1:4:1) with FA and Alb, associated with a decrease in the percentage of cells in the S or G_0_/G_1_ phases (Fig. 3A and Suppl. Fig. S5). However, exposure of OPM-1 cells to 3NPs triggered an increase in the proportion of cells in the S phase, whereas pronounced G_0_/G_1_ block was detected in OPM-2 cells after treatment with all 3NPsat 24 h (Fig. 3A). At 48 h, the proportion in the G_2_/M phase was increased (Suppl. Fig. S5).

To further evaluate the molecular mechanisms accompanied by the cell cycle, we examined the association of 3NPs with the increased proportion of most sensitive MM.1S cells in the G_2_/M phase of the cell cycle compared to RPMI-S cells at 24 h by western immunoblot analyses (Fig. 3B). The levels of the key cell cycle regulatory molecules ATM and ATR were decreased in all 3NPs-treated MM.1S cells, but the levels of both total ATM and ATR proteins were higher in RPMI-S cells. On the other hand, an increase in p-ATR and a decrease in p-ATM was detected in MM.1S cells treated with 3NPs, whereas lower activation of phosphorylated forms p-ATM and p-ATR was observed in RPMI-S cells treated with 3NPs. Treatment with 3NPs showed a dose-dependent phosphorylation of checkpoint kinase 2 (p-Chk2) and a decrease in Chk2 and checkpoint kinase 1 (Chk1) levels in MM.1S cells, while upregulation of Chk1 expression was detected in RPMI-S cells without changes to p-Chk2 or total Chk2 levels. Moreover, upregulation of Cyclin B1 without a significant change in p-Cdc2 and total Cdc2 protein levels triggered by 3NPs was detected in MM.1S cells, whereas no changes were detected in RPMI-S cells. In addition, exposure to 3NPs markedly increased the levels of BRD4 and histones, including phosphorylated histone H3 (p-H3), histone H3 (H3; only in MM.1S cells), phosphorylated histone H2AX (p-H2AX), and histone H2AX (H2AX), with more pronounced effects in MM.1S cells than in RPMI-S cells. Overall, the anti-MM effects of all 3NPs were associated with an accumulation of cells in G_2_/M cell cycle arrest, which was accompanied by the changes in the levels of regulatory and signaling molecules in the cell cycle.

### Molecular mechanism triggered by 3NPs in MM cell lines

**Figure 4:**
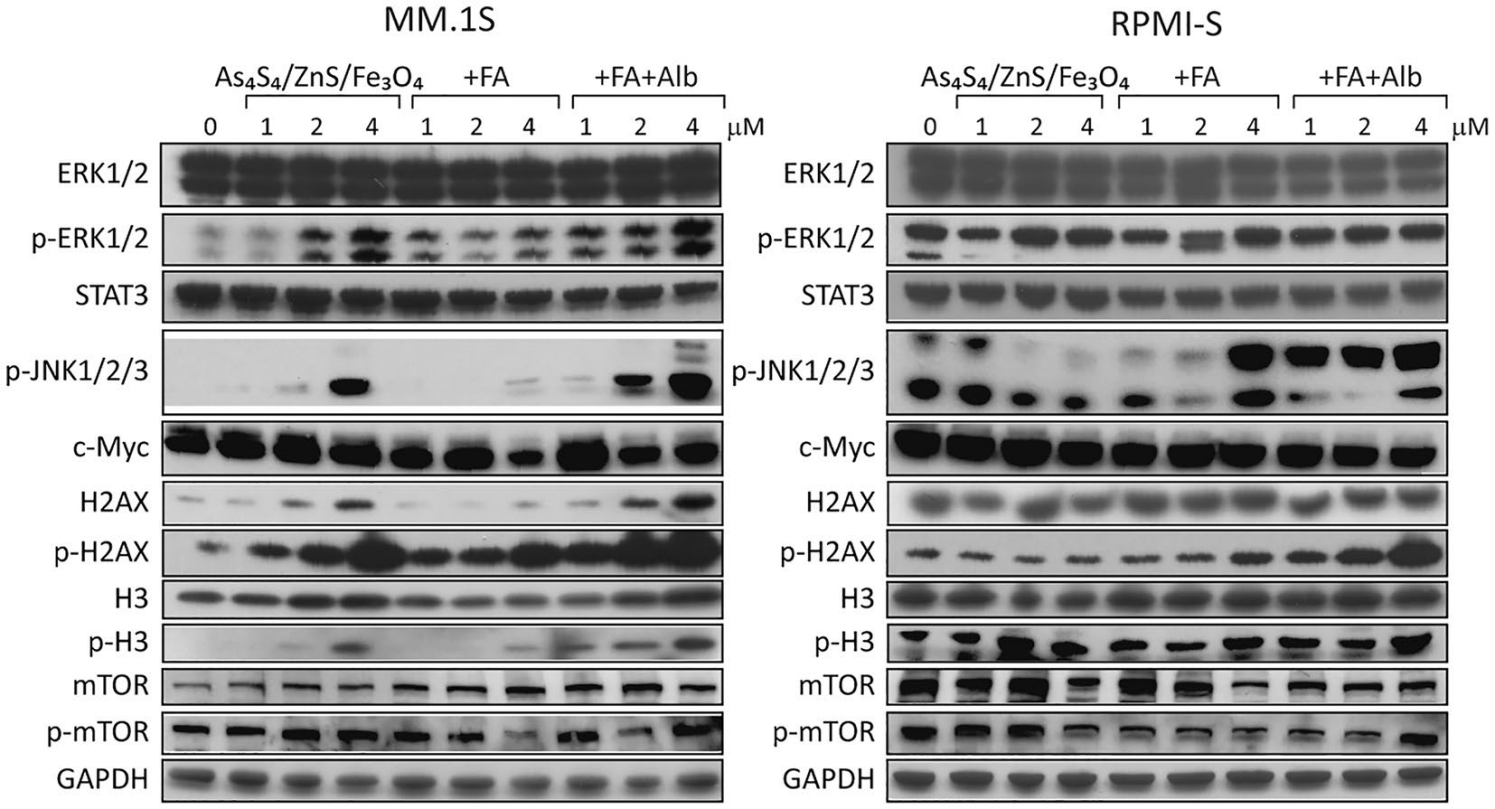
As_4_S_4_/ZnS/Fe_3_O_4_ (1:4:1), As_4_S_4_/ZnS/Fe_3_O_4_ (1:4:1) with FA, and As_4_S_4_/ZnS/Fe_3_O_4_ (1:4:1) with FA and Alb induce activation in several signaling pathways and modulate expression of key regulatory molecules. MM.1S and RPMI-S cells were cultured with all 3NPs at 1, 2, and 4 µM for 8 h. Protein concentrations of whole cell lysates were measured using a Bradford protein assay kit. Western blot analysis from whole cell lysates (20 μg of proteins/lane) were immunoblotted using anti-ERK1/2, -p-ERK1/2, -STAT3, -p-JNK1/2/3, -c-Myc, -H2AX, -p-H2AX, -H3, -p-H3, -mTOR, and -p-mTOR antibodies. GAPDH served as a loading control. Results are representative of two independent experiments.

To examine changes in the levels of early molecular and signaling molecules in MM.1S and RPMI-S cells after treatment with the composite nanoparticles, several different mechanisms affecting signal transduction pathways and cell functions were evaluated after 8 h of exposure to all 3NPs (Fig. 4). Various mitogen-activated protein (MAP) kinases, such as extracellular signal-regulated kinase (Erk) 1 and 2 and c-Jun amino-terminal kinase (JNK or SAPK), were mostly activated/phosphorylated in MM.1S cells, with no significant changes in the expression of the total proteins ERK1/2, STAT3, or. Moreover, treatment with 3NPs increased the activation (phosphorylation) of histones (p-H3 and p-H2AX) in a dose-dependent manner in MM cells, which exhibited an increase in the total levels of histones H3 and H2AX only in MM.1S cells. Similarly, early upregulation of p-mTOR by 3NPs treatment was also detected in MM cells, whereas modulations of mTOR levels were detected in both MM cell lines. However, composite 3NPs did not change c-Myc level in MM.1S and RPMI-S cells after 8 h duration. Overall, our data indicate modulations in the levels of several MM-associated signaling pathways after 3NPs treatment.

### BM stromal cells did not attenuate the anti-MM activity of 3NPs

**Figure 5:**
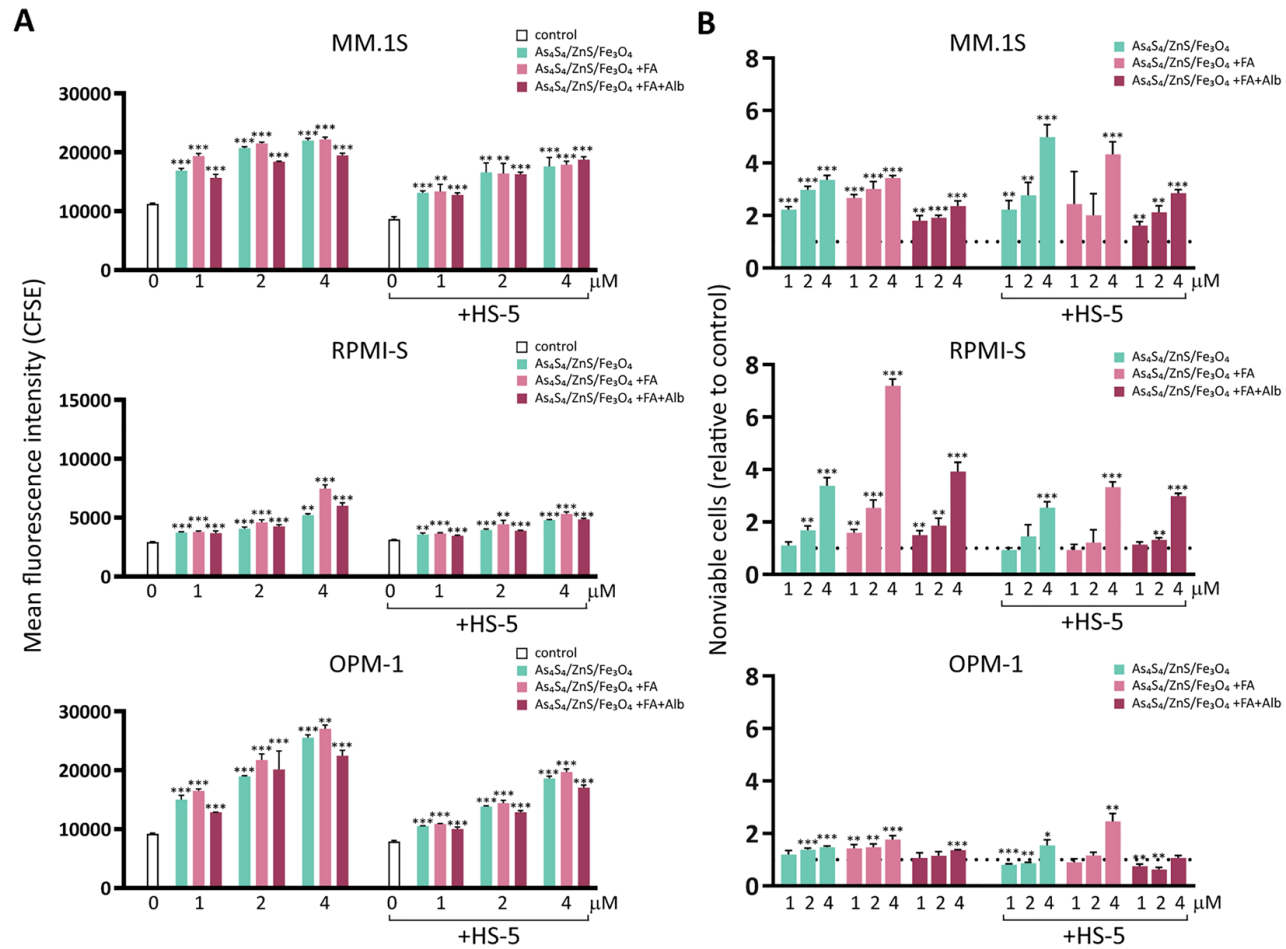
As_4_S_4_/ZnS/Fe_3_O_4_ (1:4:1), As_4_S_4_/ZnS/Fe_3_O_4_ (1:4:1) with FA, and As_4_S_4_/ZnS/Fe_3_O_4_ (1:4:1) with FA and Alb inhibit proliferation of MM cells alone and in co-culture with bone marrow stromal cells (BMSC). Carboxyfluorescein diacetate succinimidyl ester (CFSE)-stained MM.1S, RPMI-S and OPM-1 cells, alone or in co-culture with BMSC HS-5 cells, were treated with all 3NPs at 1, 2, and 4 µM for 48 h. Non-viable MM (CFSE +/PI +) cells were determined by PI staining and analyzed by a FACS Canto II flow cytometer. (**A**) Fluorescence intensity of gated CFSE^+^PI^−^-stained MM cells is shown as a function of all 3NPs concentration (1, 2, and 4 µM). (**B**) The ratio of fraction affected (non-viable cells) MM cells triggered by 3NPs relative to untreated controls is shown as a function of all 3NPs concentration (1, 2, and 4 µM). Each treatment with a specific concentration of NPs was done in triplicate. Data are from two independent experiments and are presented as mean ± standard deviation. Significant differences between treatments and control were identified by t-test with *p < 0.05, **p < 0.01, and ***p < 0.001.

The BM microenvironment plays an essential role in MM pathogenesis by promoting tumor growth, survival and drug resistance. Therefore, we next examined whether the BM microenvironment in the form of BM stromal cells reduced the effects of 3NPs in MM cells. Cell division was assessed by CFSE staining. MM cells were stained with CFSE and cultured either alone or with HS-5 stromal cells and then treated with different concentrations (1, 2, and 4 μM) of 3NPs for 24 and 48 h (Fig. 5 and Suppl. Fig. S6). The increase in CFSE fluorescence intensity correlates with a decrease in or inhibition of cell proliferation. Our data confirmed that 3NPs at all concentrations inhibited the proliferation of MM cells (MM.1S, RPMI-S, and OPM-1 cells) with or without HS-5 BM stromal cells at 24 h (Suppl. Fig. S6A) and 48 h (Fig. 5A). To distinguish live cells from nonviable cells, PI staining was performed. The fraction of nonviable MM cells after 3NPs treatment was significantly increased in a concentration-dependent manner at 24 h (Suppl. Fig. S6B) and 48 h (Fig. 5B). The nonviable MM cells after As_4_S_4_/ZnS/Fe_3_O_4_ (1:4:1) with/without FA treatment and cultured with HS-5 stromal cells were higher, especially in MM.1S cells at 48 h; the results were similar for RPMI-S cells without exposure to HS-5 stromal cells at 48 h. Furthermore, As_4_S_4_/ZnS/Fe_3_O_4_ (1:4:1) with FA and Alb led to a similar increase in the fraction of nonviable MM cells when cultured both alone and in the presence of stromal cells. These data indicate that 3NPs treatment can overcome BM microenvironment-mediated growth and drug resistance.

### Decrease in the stem cell-like SP phenotype by 3NPs-treated MM cell lines

**Figure 6:**
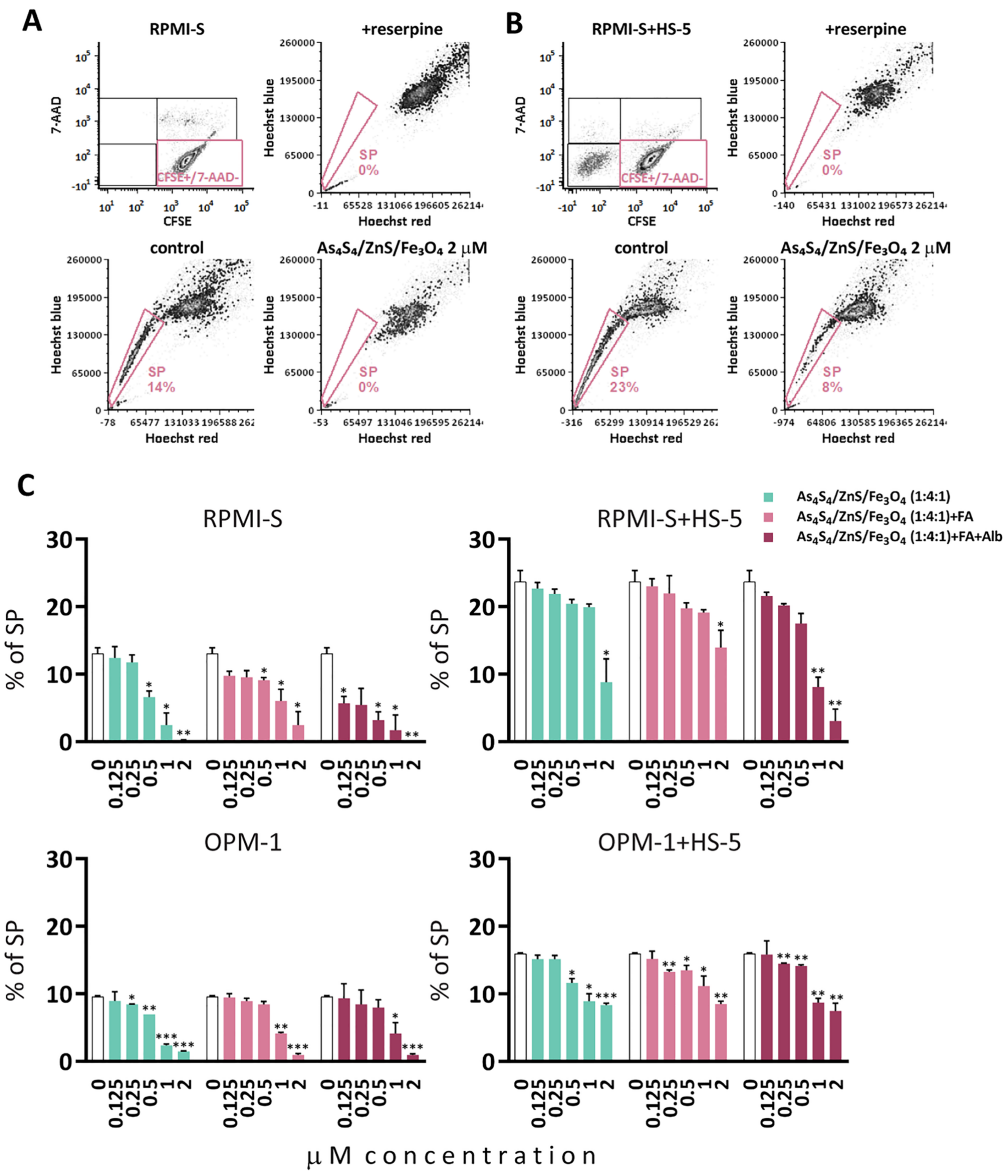
As_4_S_4_/ZnS/Fe_3_O_4_ (1:4:1), As_4_S_4_/ZnS/Fe_3_O_4_ (1:4:1) with FA, and As_4_S_4_/ZnS/Fe_3_O_4_ (1:4:1) with FA and Alb decrease SP fraction of MM cells alone and in co-culture with bone marrow stromal cells. (**A**) Carboxyfluorescein diacetate succinimidyl ester (CFSE)-stained RPMI-S and OPM-1 cells were treated with all 3NPs at 0.125, 0.25, 0.5, 1, and 2 µM for 48 h, stained by Hoechst 33,342 dye, and analyzed by FACS Aria Special Sorter UV laser flow cytometer. Viable MM (CFSE +/7-AAD-) cells without BMSC HS-5 cells were determined by 7-aminoactinomycin D (7-AAD) staining (upper row, left dot plot). Low intracellular fluorescence of Hoechst 33,342 to detect SP cell fraction was analyzed by gating only on viable CFSE +/7-AAD- MM cells using De Novo FCS Express software. SP cells are determined by low intracellular staining with Hoechst 33,342 fluorescence dye (lower row, left dot plot). SP disappeared when cells were treated with reserpine (upper row, right dot plot) and 2 µM As_4_S_4_/ZnS/Fe_3_O_4_ (lower row, right dot plot). (**B**) CFSE-stained RPMI-S and OPM-1 cells in co-culture with HS-5 cells were treated with all 3NPs at 0.125, 0.25, 0.5, 1, and 2 µM for 48 h, stained by Hoechst 33,342 dye, and analyzed by FACS Aria Special Sorter UV laser flow cytometer. Viable MM (CFSE +/7-AAD-) cells in the context of BMSC HS-5 cells (CFSE −/7-AAD-) were determined by 7-AAD staining (upper row, left dot plot). Low intracellular fluorescence of Hoechst 33,342 to detect SP cell fraction was analyzed by gating only on viable CFSE +/7-AAD- MM cells using De Novo FCS Express software. SP cells are determined by low intracellular staining with Hoechst 33,342 fluorescence dye (lower row, left dot plot). SP disappeared when cells were treated with reserpine (upper row, right dot plot) and decreased by 2 µM As_4_S_4_/ZnS/Fe_3_O_4_ (lower row, right dot plot). (**C**) Data are representative of three independent experiments with triplicates and are presented as mean ± standard deviation. Significant differences between treatments and control were identified by t-test with *p < 0.05, **p < 0.01, and ***p < 0.001.

Previously, our data have shown that realgar (As_4_S_4_) nanoparticles significantly decreased the fraction and clonogenicity of the MM stem cell-like side population (SP) in MM cells^19^ alone or with the BM stromal cells^16^. To evaluate the effects of 3NPs on the proportion of the cell SP fraction, we treated CFSE-labeled MM cells (with a high SP fraction, such as RPMI-S and OPM-1 cells), cultured either alone or with HS-5 BM stromal cells, with different nontoxic concentrations (0.125, 0.25, 0.5, 1, and 2 μM) of all 3NPs for 48 h. Low intracellular content of Hoechst 33,342 dye determined by flow cytometry analysis represents SP cells that disappeared when cells were treated with the multi-ABC transporter inhibitor reserpine. The highest concentration (2 μM) of As_4_S_4_/ZnS/Fe_3_O_4_ significantly abolished SP cells in RPMI-S cells alone, in addition attenuated SP fraction in RPMI-S cells in the presence of HS-5 stromal cells (Fig. 6A and B). The flow cytometry evaluation of MM cells for low fluorescence intensity of Hoechst 33,342 dye showed that 3NPs significantly downregulated the SP fraction in MM cells and even in the co-culture with HS-5 stromal cells in a dose-dependent manner at 48 h (Fig. 6C). The highest decrease in the proportion of the stem cell-like SP was observed for As_4_S_4_/ZnS/Fe_3_O_4_ (1:4:1) with FA and Alb treatment in RPMI-S cells and for As_4_S_4_/ZnS/Fe_3_O_4_ (1:4:1) in OPM-1 cells, both in the absence and presence of HS-5. Our data showed that all 3NPs significantly decreased the stem cell-like SP in MM cells, even in the presence of the BM stromal cells.

### Anti-MM agents enhance cytotoxicity in combination with 3NPs

**Figure 7:**
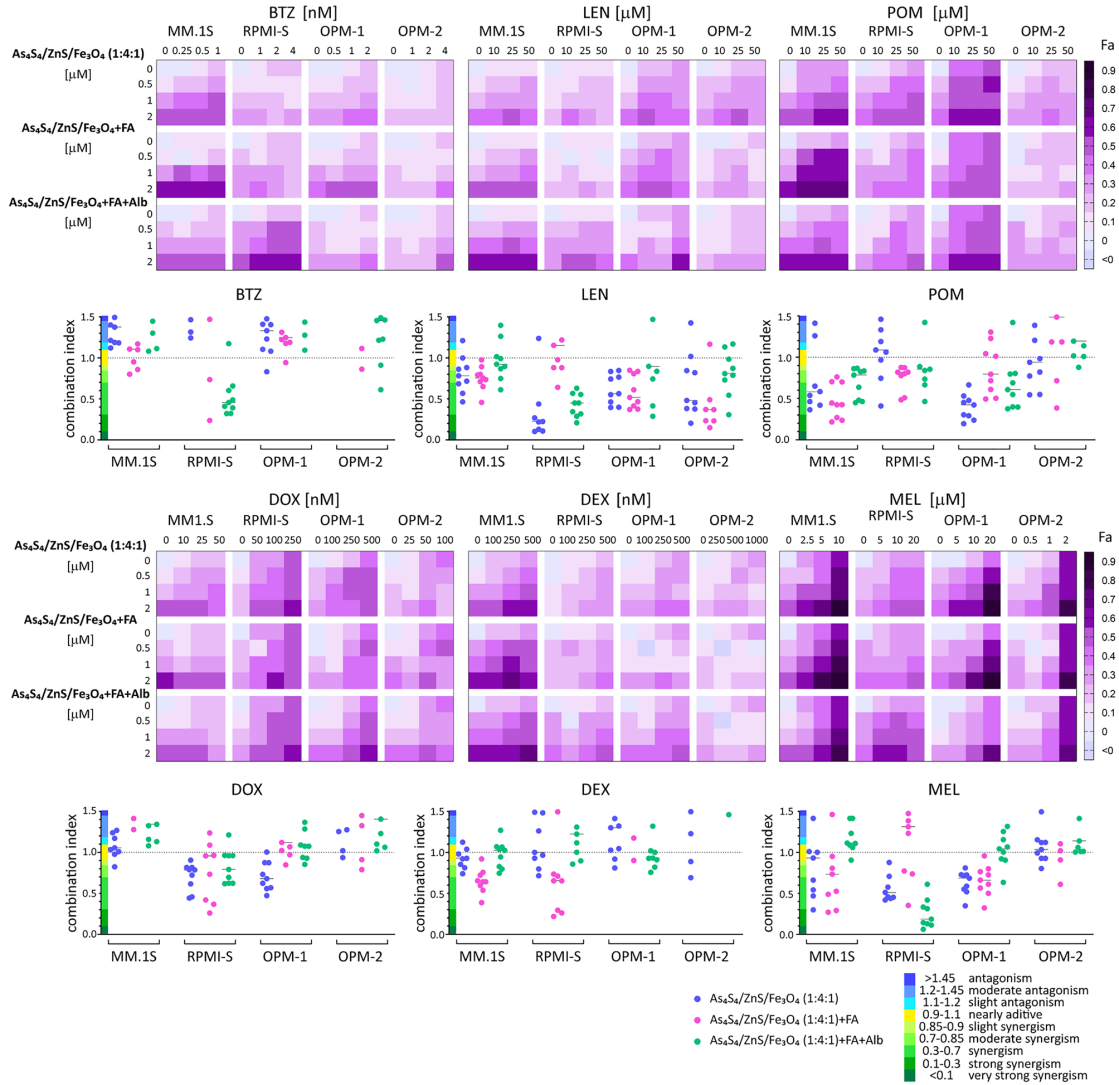
As_4_S_4_/ZnS/Fe_3_O_4_ (1:4:1), As_4_S_4_/ZnS/Fe_3_O_4_ (1:4:1) with FA, and As_4_S_4_/ZnS/Fe_3_O_4_ (1:4:1) with FA and Alb enhance the effect of anti-MM agents in vitro. MM.1S, RPMI-S, OPM-1, and OPM-2 cells were cultured with all 3NPs in combination with novel anti-MM drugs: bortezomib (velcade; BTZ), lenalidomide (LEN) and pomalidomide (POM) and conventional anti-MM drugs: doxorubicin (DOX), dexamethasone (DEX) and melphalan (MEL) for 48 h. Cell viability was then assessed using the MTT assay. Fractions-affected (Fa—ratio of number of nonviable MM cells/total number of MM cells) cells was visualized in a color-coded format and compared to treatment with each drug alone. Isobologram analysis was performed to calculate the combination index (CI) for each combination by the Chou–Talalay method. The x-axis corresponds to the fractional effect at various combination doses and the y-axis represents the CI; CI < 1 indicates drug synergy, whereas CI = 1 is considered additive, and CI > 1 indicates antagonism. All experiments were performed in triplicate. Data represent mean (± standard deviation) of triplicate cultures.

Combinations of novel and/or conventional anti-MM treatments show better clinical response outcomes than single therapies. Therefore, we evaluated whether all 3NPs enhance cytotoxicity when administered in combination with novel anti-MM agents (bortezomib, lenalidomide, and pomalidomide) and conventional drugs (dexamethasone, doxorubicin and melphalan). The anti-tumor activity of combination treatments was analyzed in MM cell lines (MM.1S, RPMI-S, OPM-1, and OPM-2 cells) at 24 h and 48 h by MTT assay, and the effects were evaluated using CalcuSyn software. The fraction of affected cells (Fa) is presented in heatmaps, and the combination indices (CI) for each of the combinations are presented in isobologram graphs (Fig. 7 and Suppl. Fig. S7).

The proteasome inhibitor bortezomib (BTZ) showed an additive or slight/moderate synergistic effects (defined according to the criteria detailed by Chou-Talalay)^20^ in combination with all 3NPs in all 4 MM cell lines at 24 h. On the other hand, prolonged treatment for 48 h decreased the synergistic effects and led mostly to antagonism, except in RPMI-S cells treated with As_4_S_4_/ZnS/Fe_3_O_4_ (1:4:1) with FA and Alb combined with BTZ, for which synergism was detected at 24 h and 48 h. The first-generation immunomodulatory agent lenalidomide (LEN) combined with all 3NPs at 24 h observed mostly additive or slight/moderate synergism, except in OPM-1 and OPM-2 cells, for which synergism was detected with As_4_S_4_/ZnS/Fe_3_O_4_ (1:4:1) with/without FA and As_4_S_4_/ZnS/Fe_3_O_4_ (1:4:1), respectively. In contrast to BTZ, combined treatment with 3NPs and LEN for longer periods augmented synergism with 48 h in MM cells. The second-generation immunomodulatory drug pomalidomide (POM), when combined with each of 3NP type, showed mostly moderate synergistic to synergistic effects, except antagonism was observed for the combinations of As_4_S_4_/ZnS/Fe_3_O_4_ (1:4:1) with FA or As_4_S_4_/ZnS/Fe_3_O_4_ (1:4:1) with FA and Alb with POM in OPM-2 cells at 24 h; this effect was mitigated within 48 h (Fig. 7 and Suppl. Fig. S7).

Our analyses showed that all 3NPs combinations with doxorubicin (DOX) exerted mostly additive or even antagonistic effects in MM.1S, RPMI-S, and OPM-1 cells, except for a few combinations of As_4_S_4_/ZnS/Fe_3_O_4_ (1:4:1) with DOX in OPM-2 cells at 24 h, which led to synergism. In addition, synergism was detected when all 3NPs were combined with DOX in RPMI-S cells and when As_4_S_4_/ZnS/Fe_3_O_4_ (1:4:1) was combined with DOX in OPM-1 cells at 48 h. Moreover, combinations of all 3NPs with dexamethasone (DEX) showed mostly antagonistic effects, except for a few concentrations of As_4_S_4_/ZnS/Fe_3_O_4_ (1:4:1) and As_4_S_4_/ZnS/Fe_3_O_4_ (1:4:1) with FA in combination with DEX, which detected slight synergism in OPM-1 and OPM-2 cells at 24 h. Similarly, additive or antagonistic effects were observed at 48 h, with the exception of combinations of As_4_S_4_/ZnS/Fe_3_O_4_ (1:4:1) with FA (in MM.1S and RPMI-S cells) with DEX exhibiting synergism. On the other hand, synergism was confirmed in combinations of As_4_S_4_/ZnS/Fe_3_O_4_ (1:4:1) (in MM.1S, RPMI-S and OPM-1 cells) and As_4_S_4_/ZnS/Fe_3_O_4_ (1:4:1) with FA (in MM.1S, OPM-1 and OPM-2 cells) with melphalan (MEL) in MM cells at 24 h and 48 h. Additive effects or even antagonism were mostly observed in combinations of As_4_S_4_/ZnS/Fe_3_O_4_ (1:4:1) with FA and Alb with MEL in MM cells at 24 h and 48 h, except for synergism detected in RPMI-S cells at 48 h (Fig. 7 and Suppl. Fig. S7). The strong synergistic effects of all 3NPs combined with lenalidomide or pomalidomide, as well as As_4_S_4_/ZnS/Fe_3_O_4_ (1:4:1) or As_4_S_4_/ZnS/Fe_3_O_4_ (1:4:1) with FA combined with melphalan, suggest the potential use of these combinations in future clinical studies.

## Discussion

Nanoparticles, small submicron particles between 5 and 200 nm in diameter, have a wide range of applications in biomedicine. Different classes of NPs derived either from (i) organic-based nanoparticles, including liposomes^21,22^, polymeric nanoparticles^23^, micelles, vesicles^24,25^, dendrimers, or carbon nanotubes^26^, or (ii) inorganic-based nanoparticles (such as semiconductor-based quantum dots)^27^ and solid nanoparticles comprised of gold, zinc oxide, iron oxide or other metallic components^28–30^ have been utilized in the past, depending on the application. Liposomes display biocompatibility and high drug-loading capability, mostly water-insoluble drugs, and incorporate different functionalities. In particular, PEG (polyethylene glycol)-ylated liposomal nanoparticles encapsulated with DOX and carfilzomib (CFZ)^11^ or DEX^22^; BCMA (B-cell maturation antigen)-targeted liposomes ^21^; and VLA-4 (Very Late Antigen-4, also known as α4β1 integrin) and LPAM-1 (Leukocyte Peyer’s Patch Adhesion Molecule-1, also known as α4β7 integrin) dual-targeted liposomes^31^ show potent anti-myeloma activity. Similar to liposomes, micelles are artificial vesicles formed by the self-assembly of amphiphilic lipids, which enclose a hydrophobic core and encapsulated hydrophobic drugs. Most polymeric micelles, such as PEG conjugates prepared either from PEG-b-polydithiolane trimethylene-co-ε-caprolactone (PEG-P(DTC-co-CL)) copolymers^25^ or PEG-b-poly(n-2-benzoyloxypropyl methacrylamide (mPEG-b-p(HPMA-Bz))^24^ to deliver CFZ; hyaluronic acid-shelled and core-disulfide-crosslinked biodegradable micelles (HA-CCMs) to deliver BTZ^32^; and titanocene (TC) micelles to deliver radiolabeled fluorodeoxyglucose (^18^FDG)^33^ to myeloma cells, have been evaluated. In addition, polymeric nanoparticles constituted by chitosan or poly(lactic-co-glycolic acid) (PLGA), such as chitosan-PLGA encapsulating miR-34a^34^, PLGA loaded with LEN, chitosan loaded with BTZ^35^, and targeted BCMA-specific peptide-encapsulated PLGA nanoparticles^21^ have been developed. In addition, Dickkopf-1 and programmed death-ligand 1 (PD-L1) have been delivered by PLGA/PEI nanoparticles^36^. Lipoic acid-crosslinked hyaluronic acid nanoparticles loaded with DOX^37^, a catechol-functionalized polycarbonate core that encapsulates BTZ^38^, and anti-CD38 antibody–conjugated poly(ethyleneoxide)-block-poly(-benzylcarboxylate-”-caprolactone) (PEO-b-PBCL) NPs loaded with S3I-1757, an inhibitor of STAT3^30^, have been developed as potential therapeutic candidates in the treatment of MM. Furthermore, advanced drug delivery systems, bioimaging, and diagnostic tools based on inorganic nanoparticles, including gold^29^, iron^30,39^ or zinc oxide^28^, and titanium dioxide nanoparticles^40^, as well as snake venom-loaded silica nanoparticles^41^, have been widely investigated for MM therapy. Carbon-based materials characterized by a graphene structure, such as graphene oxide conjugated with DOX^23^ or PEG-modified cadmium telluride quantum dots loaded with DOX ^27^, and carbon nanotubes with metastasis-associated lung adenocarcinoma transcript 1(MALAT1)^26^ have received significant interest in anti-myeloma therapy.

The multifunctional nanocomposite with the three inorganic components As_4_S_4_/ZnS/Fe_3_O_4_ demonstrated therapeutic, magnetic and optical functionality and was prepared by high-energy milling in dry mode^15^. Nanocomposite suspensions were covered with biocompatible PX407 solution, a nonionic surfactant composed of polyethylene-polyoxypropylene triblock copolymers, to prevent aggregation and to form a highly oxidized state via the process of wet stirred media milling. Moreover, coating with PX407 provided better biomedical functionality and NPs stability and less toxicity compared to those of other copolymers^42^. Advanced milling technologies, such as nanocrystalline dispersion via media milling processes^43^, fracture micron-sized drug crystals into homogeneous nanoparticle dispersions < 200 nm in diameter in a reproducible manner. A unimodal particle size distribution with an average particle diameter of 120–140 nm, long-term stability and optical fluorescence properties were the basic characteristics of our As_4_S_4_/ZnS/Fe_3_O_4_ nanocomposite^15^. In ancient and modern medicine, arsenic as an active compound, including orpiment (As_2_S_3_), realgar (As_4_S_4_), and arsenolite-arsenic trioxide (As_2_O_3_), has been effective in treating various hematological cancers, such as acute promyelocytic leukemia (APL), myelodysplastic syndrome, and MM^44–46^. Arsenic trioxide (ATO) is approved for the treatment of relapsed/refractory APL patients^46,47^ and shows high response rates, increases survival and presents a favorable toxicity profile even in standard front-line therapy^48,49^. Similarly, realgar (As_4_S_4_; REA) achieves a high rate of complete remission, long disease-free survival and acceptable side effects for APL patients^50^. To overcome poor As_4_S_4_ solubility in aqueous and in most organic solvents, due to its high intrinsic lattice energy^51^, modification of insoluble As_4_S_4_ (REA) into nanoparticles (NREA) was performed, increasing the dissolution rate and surface area by one or more orders of magnitude^52^ and thus improving bioavailability. Our preclinical in vitro and in vivo studies showed that NREA (< 150 nm nanoparticles) demonstrated greater anti-cancer activity than ATO^16^, suggesting its promise as a potential novel therapy in MM. Magnetic nanoparticles, especially biocompatible Fe_3_O_4_ (magnetite), can be easily separated from the matrix via an external magnetic field and target therapeutic agents^53^. Zinc sulfide nanoparticles, ZnS, are extensively studied semiconductor materials with optical properties that can be used in the production of luminescent NPs with multifunctional properties for novel diagnostic, therapeutic and imaging technologies in biomedicine^54,55^. When conjugated to drugs or diagnostic markers, FA retains its ability to bind to the folate receptor, which is overexpressed in most cancers, but not in healthy tissues^56^. Bovine serum albumin, the most abundant and well-characterized protein in mammalian plasma, is commonly used as a biomolecule to cover NPs, mostly sulfide NPs such as MnS and CuS, which are used in gas and photothermal therapies, respectively^57,58^.

In this study, we assessed the cytotoxic effects of composite nanoparticles involving arsenic in the form of NREA (As_4_S_4_), ZnS and Fe_3_O_4_ at a ratio of 1:4:1, As_4_S_4_/ZnS/Fe_3_O_4_ (1:4:1) with FA, and As_4_S_4_/ZnS/Fe_3_O_4_ (1:4:1) with FA and Alb in MM. We observed significant dose- and time-dependent cytotoxicity against a panel of MM cell lines, covering the most common molecular MM subtypes, with an EC_50_ in the range of 2–40.7 μM for the majority of MM cells, with the highest anti-tumor activity shown by As_4_S_4_/ZnS/Fe_3_O_4_ (1:4:1) with FA and Alb. The respective EC_50_ values of all 3NPs, As_4_S_4_/ZnS/Fe_3_O_4_ (0.7–40.7 μM), As4S4/ZnS/Fe_3_O_4_ (1:4:1) with FA (0.6–20.5 μM), and As_4_S_4_/ZnS/Fe_3_O_4_ (1:4:1) with FA and Alb (0.72–6.68 μM), were higher than those of NREA alone (0.24–1.86 μM)^16^, indicating lower or even inhibitory effects of ZnS and Fe_3_O_4_ nanoparticles. In our previous study, NREA treatment showed significant in vitro (in MM cell lines and primary patient-derived PC) and in vivo anti-MM activity in MM cell line-derived xenograft and MM patient-derived huBMsc mouse models^16^. Similarly, CuInSe2/ZnS multiparticulate nanocomposites in sodium dodecyl sulfate (SDS) nanosuspensions possess anti-myeloma sensitizing potential^59^. The favorable therapeutic window against ex vivo isolated PCs of primary myeloma patients for use of composite NPs suggested that an EC_50_ in the range 0.08–2.7 μM was on average 3–6-fold lower than that of non-PCs of the tumor microenvironment (with an EC_50_ in the range 2–18.8 μM). In addition, the cytotoxic effects of the nanocomposite 3NPs were similar to that of NREA (0.32–3 μM)^16^, suggesting their therapeutic potential in MM. The BM microenvironment consists of stromal cells, endothelial cells, immune cell subsets, and the extracellular matrix and contributes significantly to the behavior of MM cells, playing a crucial role in myelomagenesis. In particular, the interaction of MM cells with their supporting stromal cells has been key to the evolution and progression of MM because it leads to resistance to apoptosis, sustained growth and proliferation, cell homing and invasion, and stemness or self-renewal^3^. To overcome BM microenvironment-mediated MM growth and drug resistance, inhibition of MM proliferation associated with decreased MM viability induced by 3NPs was observed in cells cultured with and without BM stromal cells, with the highest anti-MM activity shown by As_4_S_4_/ZnS/Fe_3_O_4_ (1:4:1) with/without FA treatment. Similar to non-PCs, healthy mononuclear cells were significantly more resistant than either MM cell lines or primary MM patient-derived PCs. The lowest EC_50_ values, obtained for As_4_S_4_/ZnS/Fe_3_O_4_ (1:4:1) with FA and Alb, were on average threefold higher in healthy MNCs than in non-PCs and on average 19-fold higher than the CD138^+^ PC population in MM patients, suggesting a rationale for composite NPs clinical evaluation in MM.

Cellular mechanisms triggered by 3NPs in MM decreased cell survival, which was associated with apoptotic cell death accompanied by the cleavage of caspase-3, -7, -8, and PARP, an increase in the pro-apoptotic proteins Bax and Apaf-1, and a decrease in the level of the anti-apoptotic protein XIAP. Moreover, 3NPs depolarized mitochondrial membrane potential, which has been associated with caspase-9 activation. However, no significant differences in the induction of apoptosis in cells treated with each of 3NP type were detected. Similar to our previous study, NREA triggered apoptosis and was accompanied by the cleavage of caspases-3, -8, -9, and -12; an increase in the level of the pro-apoptotic protein Bax; and a decrease in the levels of the anti-apoptotic proteins Mcl-1 and Bcl-2^16^. Zinc oxide (ZnO) nanoparticles are also promising drug nanocarriers that produce fewer side effects^60^; they induce oxidative stress associated with mitochondrial dysfunction and caspase-dependent induction of apoptosis in MM^28^. Moreover, magnetic iron oxide (Fe_3_O_4_) nanoparticles combined with paclitaxel and anti-ABCG2 monoclonal antibody inhibited the proliferation of myeloma cancer stem cells with a CD138-/CD34- phenotype in correlation with the elevated expression of caspase-9, -8 and -3 and downregulation of NF-κB in vitro and in vivo^39^. Similarly, Fe_3_O_4_ nanoparticles conjugated with BTZ and gambogic acid showed anti-myeloma activity via G_2_/M block, phosphorylation of Akt, downregulation of PI3K and Bcl-2 and the induction of apoptosis by increased caspase-3 and Bax expression^30^, suggesting their potential as nanomedicines, particularly due to their biocompatibility, biodegradability, and low toxicity**.** The early cellular mechanisms triggered by 3NPs led to the accumulation of MM cells in either the G_0_/G_1_/S or G_2_/M phase, whereas prolonged treatments increased the percentage of cells in the G_2_/M phase of the cell cycle, which was associated with a decrease in the S or G_0_/G_1_ phases. G_2_/M block triggered by 3NPs was accompanied by activation (phosphorylation) of p-Chk2 and p-ATR; upregulation of cyclin B1; and a decrease in the levels of Chk1, Chk2, phosphorylated p-ATM, ATM and ATR proteins. Other molecular mechanisms accompanying the cell cycle were significantly modulated, as indicated by an increase in the levels of histones, such as phosphorylated p-H3, p-H2AX, and histones H3 and H2AX. Our previous results showed that treatment with NREA induced cell cycle arrest in the G_2_/M phase, which was associated with activation and increased expression of H3 and H2AX and increased levels of cyclin B1, p53 and its targets p21 and the pro-apoptotic protein Puma^16^. We observed early activation of the signal transduction proteins ERK1/2, JNK/SAPK, and m-TOR, but no significant changes in the expression in total ERK1/2 and STAT3 proteins. Composite 3NPs triggered dose-dependent activation of p-H3 and p-H2AX together with significant upregulation of H3 and H2AX expression. Histone phosphorylation is a cellular response to DNA damage with phosphorylated histone H2AX demarcating large chromatin domains around a DNA break site. It has been established that γ-H2AX arises in response to various types of DNA lesions and is one of the earliest responses to DNA damage^61^. Activation of the oncogenic protein MYC, which acts as a transcription factor, is one of the central molecular events leading to MM progression, and it is manifested by several mechanisms, including translocations^62,63^, the gain and amplification of 8q24.21 locus^2^, mutations in RAS genes^64^, activation of MYC translation through the PI3K/AKT/mTOR pathway^65^, MYC transcription mediated by IRF4^66^, and dysregulated LIN28B activity^67^. We showed that treatment with all 3NPs markedly downregulated c-Myc level that was associated with apoptotic cell death. Overall, the activation of early signal transduction proteins, modulation of the levels of several cell cycle- and apoptosis-associated regulators and downregulation of MM-related signaling triggered by 3NPs highlight composite NPs as strong potential therapeutic candidates for the treatment of MM.

In phase I/II clinical trials, ATO, a single agent, demonstrated only limited activity compared to BTZ and LEN in relapsed/refractory MM patients^68^, whereas an increase in the anti-MM activity of ATO in combination with ascorbic acid (AA) and MEL^69^, with AA and DEX^70^, and with AA and BTZ^71^ has been demonstrated. In addition, the combination of ATO with high-dose MEL, BTZ and AA achieved high effectiveness in relapsed/refractory MM patients in a randomized phase II clinical trial^72^, although there was toxicity associated with long-term use. In our previous preclinical study, ATO in combination with both conventional (MEL, DEX, and moderately DOX) and the novel agent LEN profoundly augmented the synergistic effects in MM, whereas antagonism was observed with BTZ^16^. On the other hand, NREA showed only synergism in combination with LEN and MEL, while either antagonistic or additive effects have been detected in combination with DEX, DOX, and BTZ^16^. In this study, we observed that composite NPs, namely As_4_S_4_/ZnS/Fe_3_O_4_ (1:4:1), As_4_S_4_/ZnS/Fe_3_O_4_ (1:4:1) with FA, and As_4_S_4_/ZnS/Fe_3_O_4_ (1:4:1) with FA and Alb in combination with immunomodulatory drugs, LEN or POM, showed significant synergistic anti-MM activity. Similarly, 3NPs showed synergism in combination with the conventional alkylating agent MEL, mainly with As_4_S_4_/ZnS/Fe_3_O_4_ (1:4:1) with/without FA. By evaluating other conventional anti-MM drugs, such as DOX and DEX, only enhanced synergism was observed in the combination of As_4_S_4_/ZnS/Fe_3_O_4_ (1:4:1) with DOX (RPMI-S and OPM-1 cells) and As_4_S_4_/ZnS/Fe_3_O_4_ (1:4:1) with FA and DEX (MM.1S and RPMI-S cells), whereas the majority of 3NPs combinations with either DOX or DEX exhibited additive or antagonistic effects. In addition, we showed mostly antagonistic effects by 3NPs in combination with the proteasome inhibitor BTZ. Overall, the synergism observed in the combination of all 3NPs with LEN or POM, as well as As_4_S_4_/ZnS/Fe_3_O_4_ (1:4:1) or As_4_S_4_/ZnS/Fe_3_O_4_ (1:4:1) with FA combined with MEL, suggests the rationale for their further evaluation in future MM clinical studies.

Myeloma cancer stem cells (CSCs), comprising a rare subpopulation of MM cells that maintain growth and proliferation, have the potential for self-renewal and contribute to drug resistance. Therefore, CSCs are considered to be the major cause of tumor recurrence or disease relapse. Previous controversial studies have identified myeloma CSCs in (i) the CD138-negative fraction, either among B cells (CD138 −/CD19 +)^73,74^ or memory B cells (CD19 +/CD27 +/CD20 +)^75^; (ii) the dominant malignant CD138-positive plasma cells^76,77^ or (iii) in the bidirectional transition from pre-plasma cells (CD19-/CD138-) to plasma cells (CD19 −/CD138 +), with clonogenic, tumorigenic and propagating potential^11^. In our previous study, we identified the “side population (SP)” based on the stem cell-like capacity to efflux Hoechst 33,342 dye by an ATP-binding cassette (ABC) membrane transporter, which is enriched in tumor-initiating cells with clonogenic and tumorigenic stem cell properties in MM^19^. Stemness-associated transcription factors, such as Sox2, Oct3/4, Nanog, Klf4, β-catenin and c-Myc, play major roles in the maintenance of myeloma stem cell-like SP cells in MM^78,79^. In addition, the major signaling pathways, including Hedgehog, Wingless, Notch and PI3K/Akt/mTOR, are involved in the regulation of self-renewal and differentiation of myeloma CSCs. In particular, BM stromal cells, the main cellular components of the BM microenvironment, and BM hypoxia are crucial for the self-renewal and survival of myeloma CSCs^19,79^. Therefore, myeloma CSCs represent promising targets for novel effective cancer therapies. In our previous study, NREA significantly reduced the MM SP cell fraction and decreased the clonogenic potential of SP cells in the absence and presence of the BM stromal cells, but no changes were observed with ATO^16^. Therefore, we evaluated the anti-SP effects of composite NPs, and all 3NPs significantly reduced the SP fraction in MM cells. Moreover, 3NPs abrogated the stimulatory effect of BM stromal cells in coculture with MM cells by significantly eliminating myeloma SP cells, suggesting their potential in anti-myeloma CSC therapies.

In summary, the anti-MM activities of composite 3NPs, As_4_S_4_/ZnS/Fe_3_O_4_ (1:4:1), As_4_S_4_/ZnS/Fe_3_O_4_ with FA, and As_4_S_4_/ZnS/Fe_3_O_4_ with FA and Alb, were confirmed by the decreased survival of MM cell lines and primary patient-derived MM cells and by higher anti-MM activities in combination with lenalidomide, pomalidomide, or melphalan. The mechanisms involved with the anti-MM activity of 3NPs included the induction of apoptosis, disruption of mitochondrial membrane potential, G_2_/M cell cycle arrest, and modulation of myeloma-associated signaling. Notably, 3NPs significantly attenuated the stem cell-like side population fraction in MM cells, even in the context of stromal cells, providing the rationale for future clinical evaluation of composite NPs to improve patient outcome in MM.

## Supplementary Information

Supplementary Information 1.

Supplementary Information 2.

Supplementary Information 3.

Supplementary Information 4.

Supplementary Information 5.

Supplementary Information 6.

Supplementary Information 7.

Supplementary Information 8.

Supplementary Information 9.

Supplementary Information 10.

Supplementary Information 11.

## Data Availability

All the data that support this study are presented in the paper or supplementary file.
