# Supplementary figures and images for: In vitro and ex vivo anti-myeloma effects of nanocomposite As4S4/ZnS/Fe3O4
Source: Sci Rep. 2022 Oct 26;12:17961. doi: 10.1038/s41598-022-22672-5 (PMC9606304; doi:10.1038/s41598-022-22672-5)

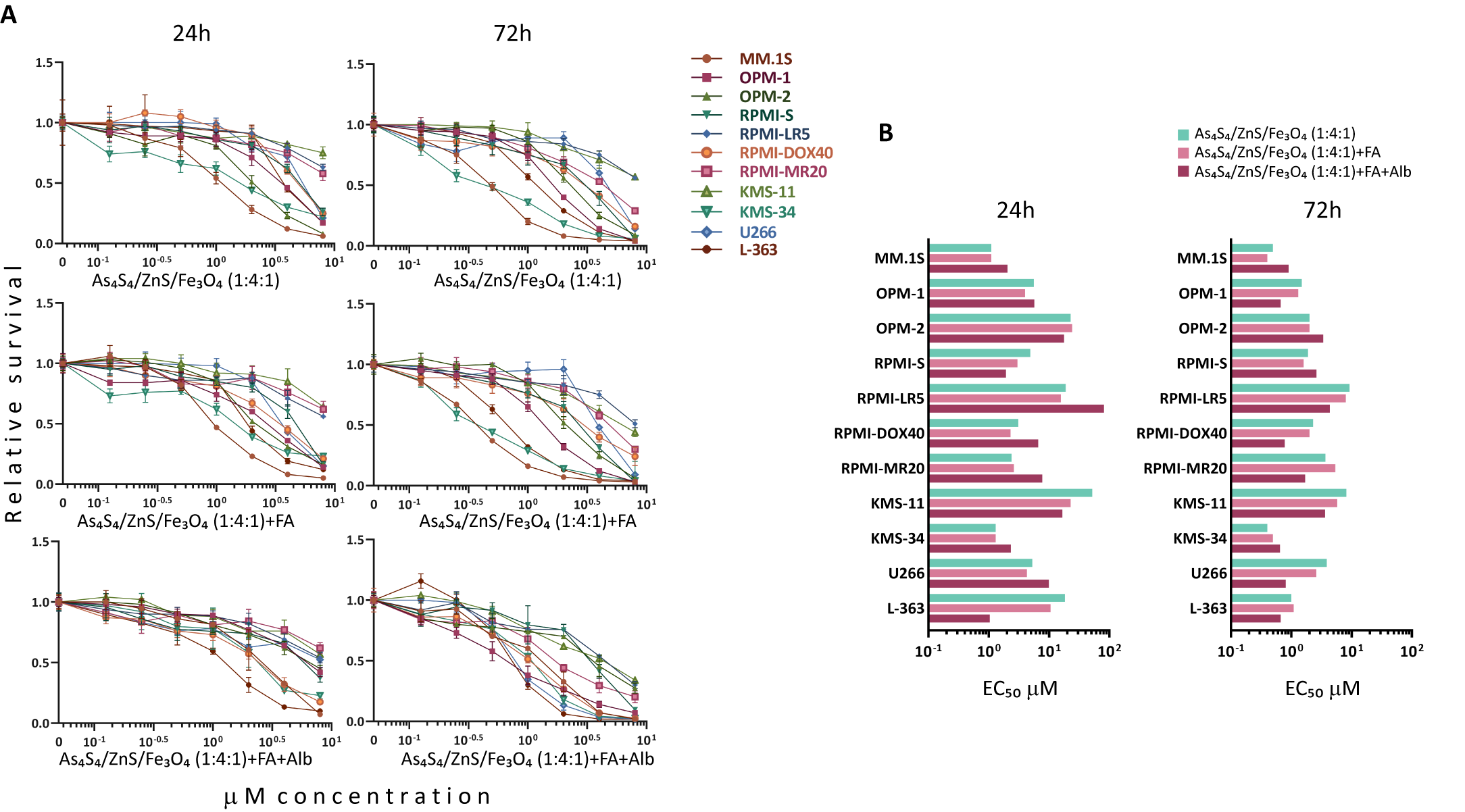

Supplement: Supplementary file 1 — Supplementary Information 1. [file 41598_2022_22672_MOESM1_ESM.tif]

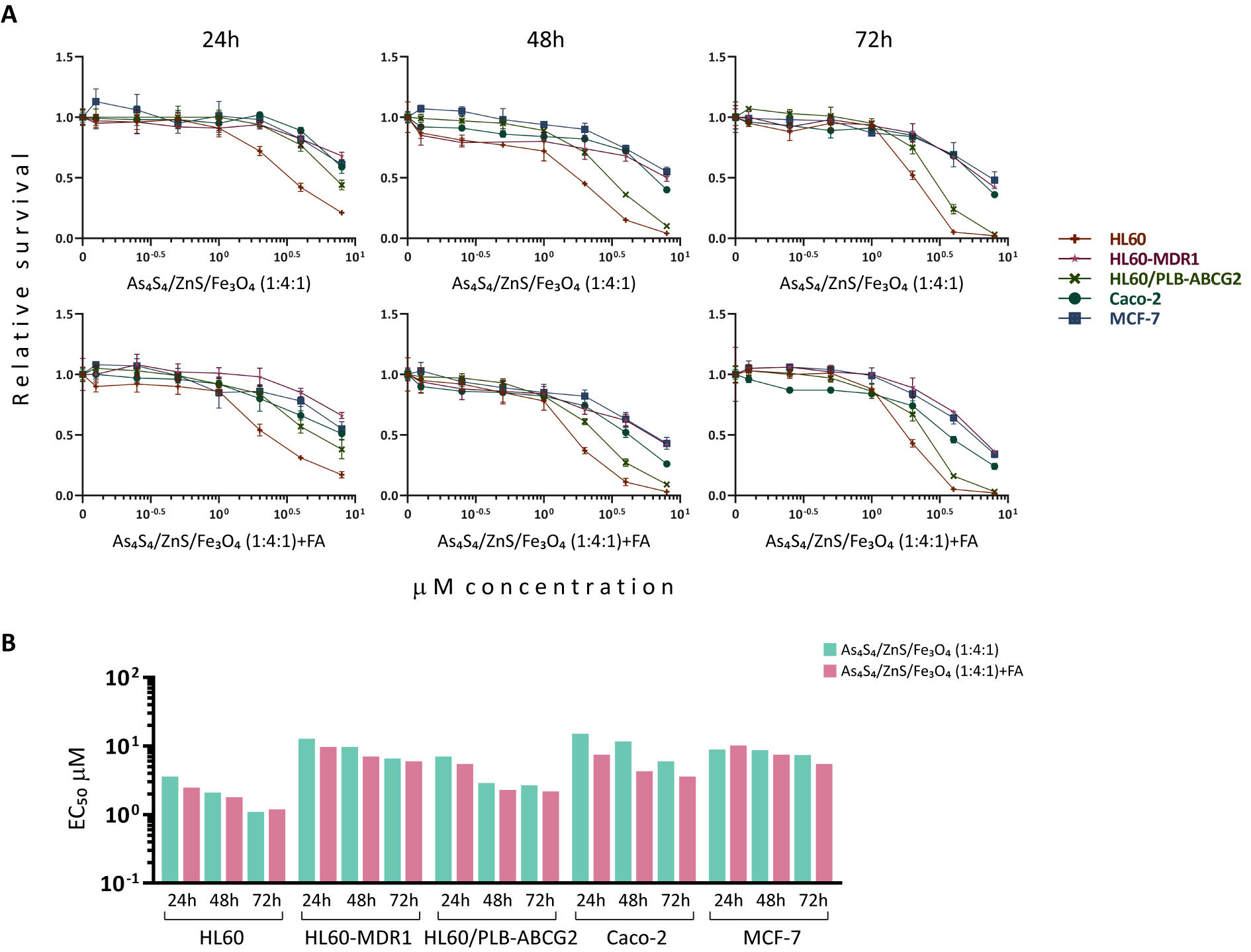

Supplement: Supplementary file 2 — Supplementary Information 2. [file 41598_2022_22672_MOESM2_ESM.tif]

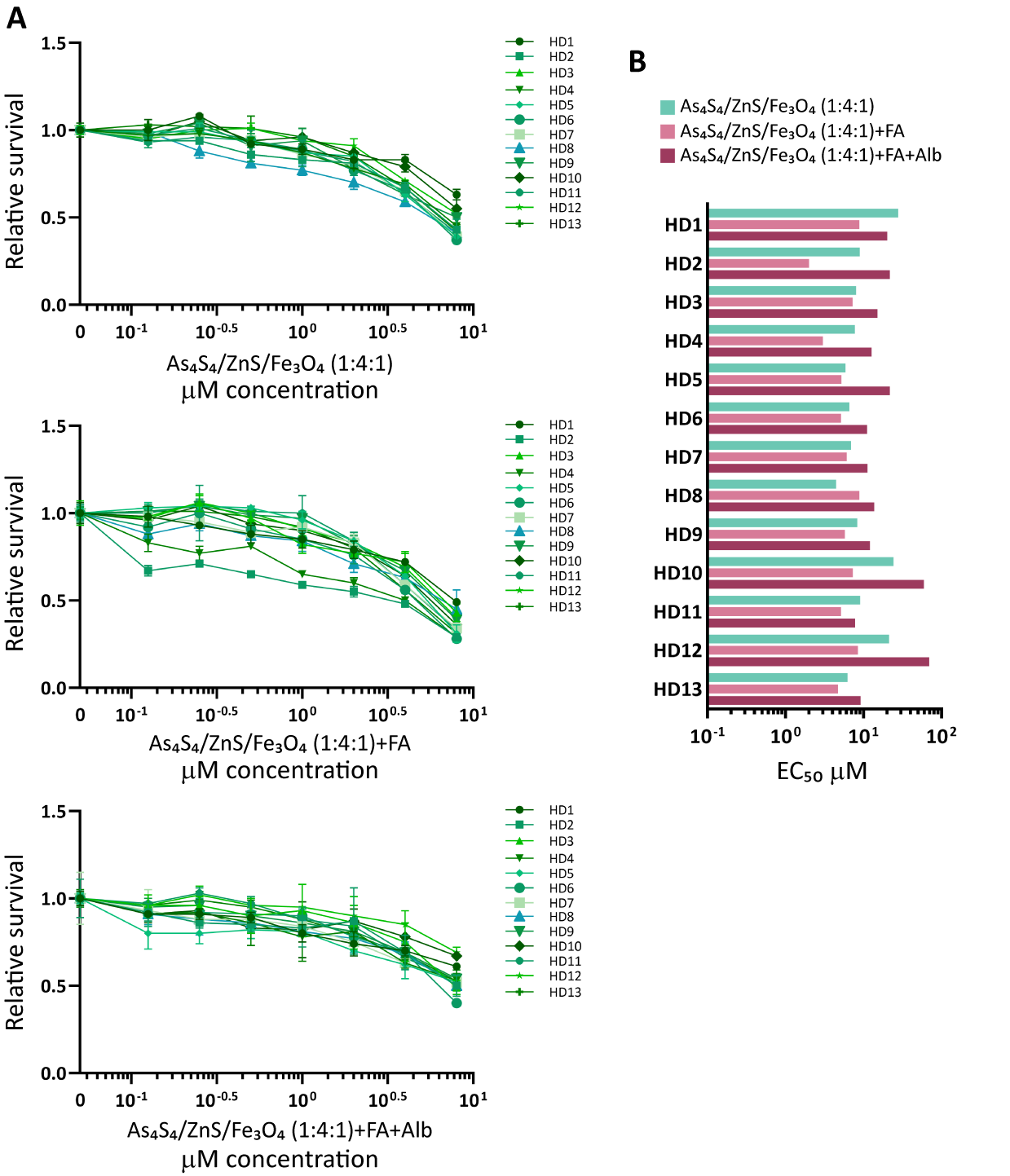

Supplement: Supplementary file 3 — Supplementary Information 3. [file 41598_2022_22672_MOESM3_ESM.tif]

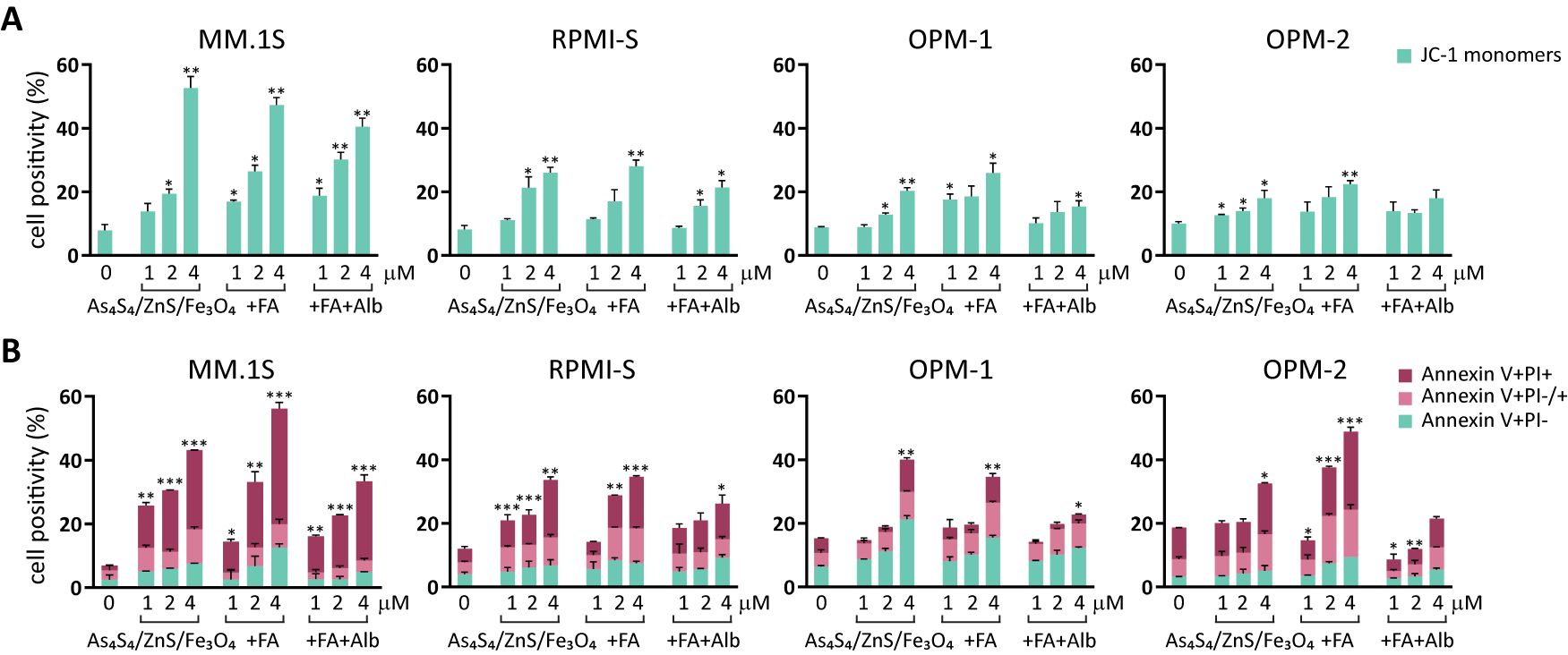

Supplement: Supplementary file 4 — Supplementary Information 4. [file 41598_2022_22672_MOESM4_ESM.tif]

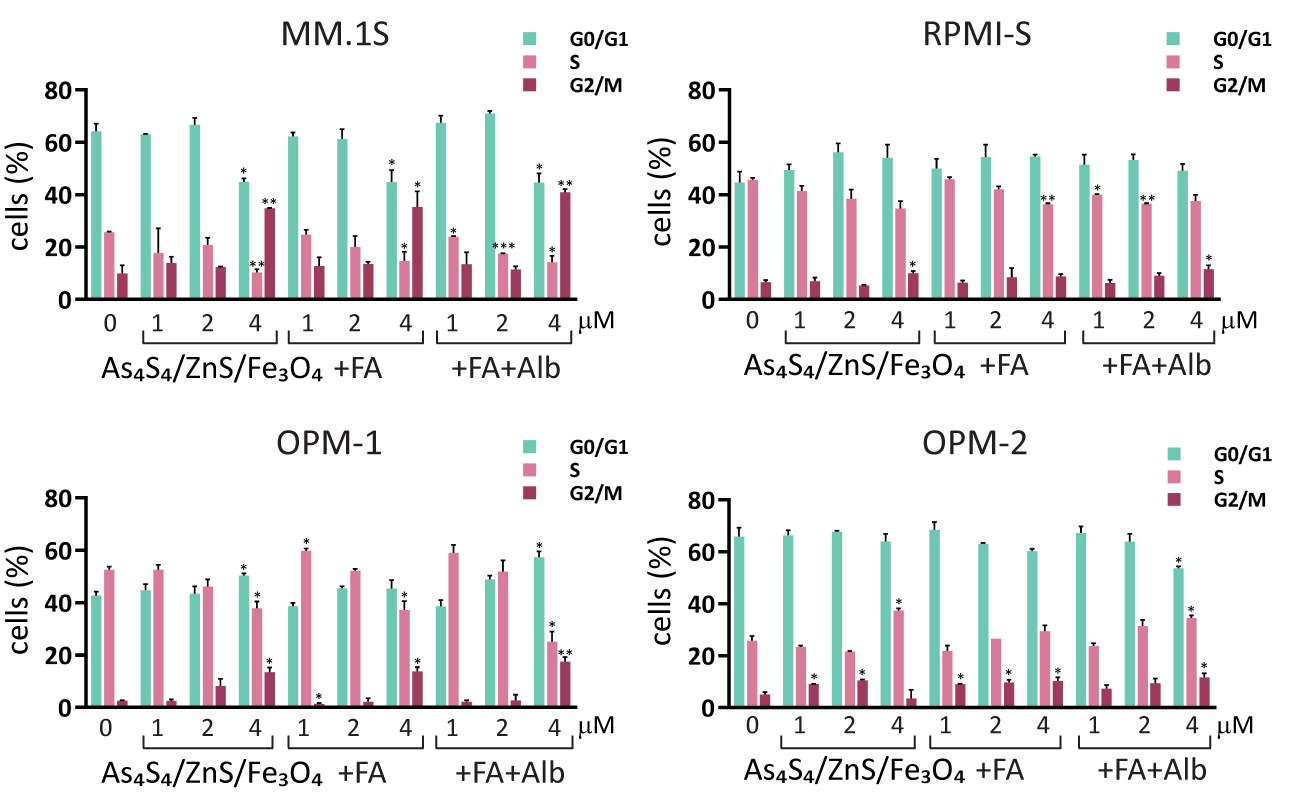

Supplement: Supplementary file 5 — Supplementary Information 5. [file 41598_2022_22672_MOESM5_ESM.tif]

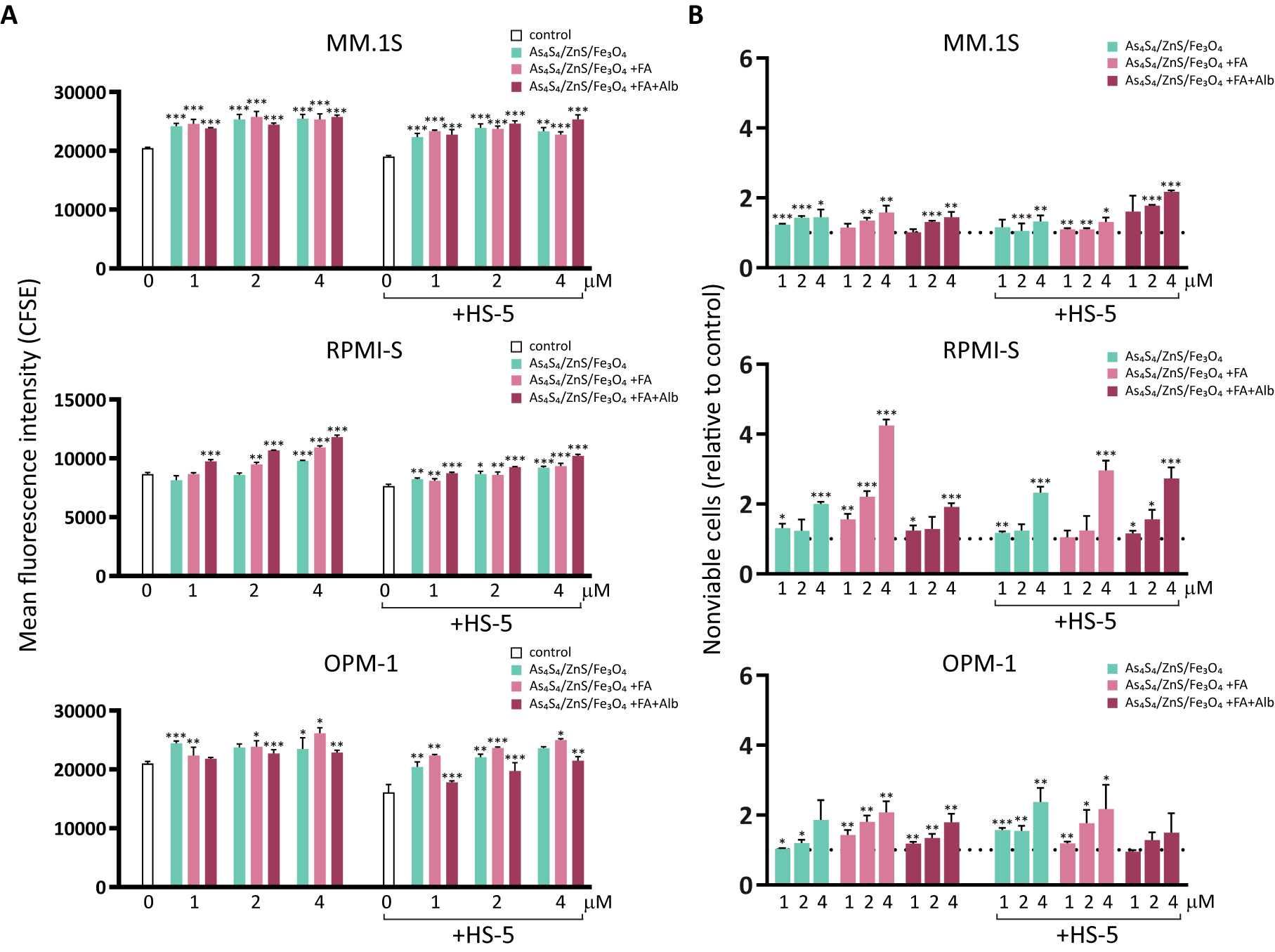

Supplement: Supplementary file 6 — Supplementary Information 6. [file 41598_2022_22672_MOESM6_ESM.tif]

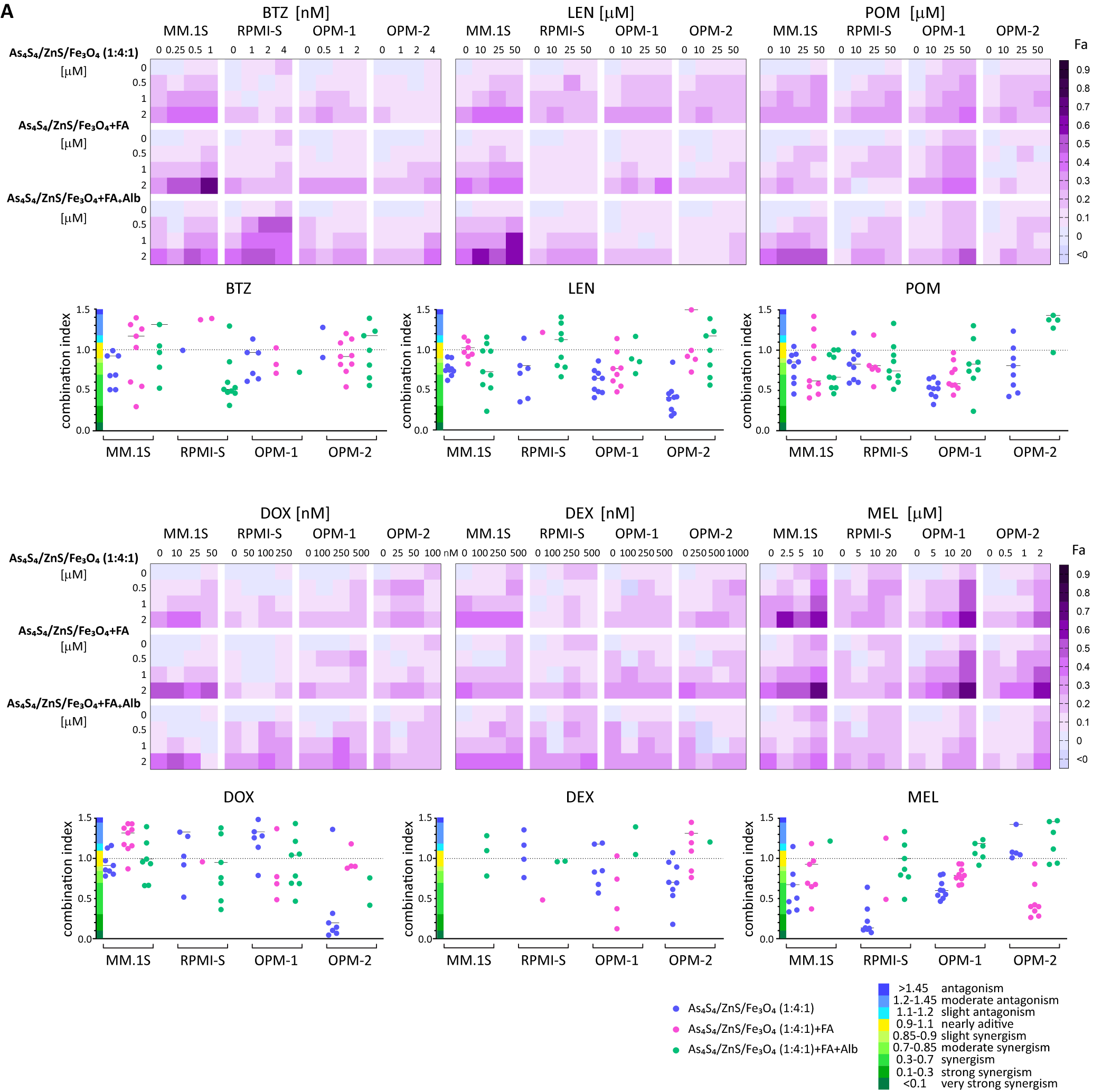

Supplement: Supplementary file 7 — Supplementary Information 7. [file 41598_2022_22672_MOESM7_ESM.tif]
