# Supplementary material for: In vitro and ex vivo anti-myeloma effects of nanocomposite As4S4/ZnS/Fe3O4
Source: Sci Rep. 2022 Oct 26;12:17961. doi: 10.1038/s41598-022-22672-5 (PMC9606304; doi:10.1038/s41598-022-22672-5)
Supplement: Supplementary file 8 — Supplementary Information 8. [file 41598_2022_22672_MOESM8_ESM.pdf]

Supplemental Methods

Drug viability assays

The inhibitory effects of $As_4S_4/ZnS/Fe_3O_4$, $As_4S_4/ZnS/Fe_3O_4$ with FA, and $As_4S_4/ZnS/Fe_3O_4$ with FA and Alb, alone or in combination with conventional or novel anti-MM agents, on survival of MM cell lines were determined by the 3-[4,5-dimethylthiazol-2-yl]-2,5-diphenyltetrazolium bromide assay (MTT; Sigma-Aldrich, St Louis, MO). MM cell lines were plated in 96-well plates at a density of 10,000 cells per well, and treated with increasing concentrations (0.125, 0.25, 0.5, 1, 2, 4, and 8 μM) of $As_4S_4/ZnS/Fe_3O_4$, $As_4S_4/ZnS/Fe_3O_4$ with FA, and $As_4S_4/ZnS/Fe_3O_4$ with FA and Alb for 24, 48 and 72 h. Formazan crystals were dissolved with addition of 150 μl of DMSO, and absorbance was measured at 540 and 690 nm in a Microplate reader (Dynatech Lab Inc., Chantilly, VA).

Purified MM patient cells and peripheral blood mononuclear cells (MNCs) were plated in 384-well plates at a density of 10,000 cells per well, and treated with increasing concentrations (0.5, 1, 2, 4, and 8 μM) of $As_4S_4/ZnS/Fe_3O_4$, $As_4S_4/ZnS/Fe_3O_4$ with FA, and $As_4S_4/ZnS/Fe_3O_4$ with FA and Alb for 48 h at 37 ºC. Viability was accessed using the CellTiterGlo (CTG; Promega) assay. CTG was added for 30 min, and plates were read with a Luminoskan luminometer (Labsystems, Franklin, MA).

The concentration of drug that inhibited cell survival to 50% (EC_{50}) was determined by Calcusyn software (Biosoft, Ferguson). To evaluate whether the interaction between $As_4S_4/ZnS/Fe_3O_4$, $As_4S_4/ZnS/Fe_3O_4$ with FA, or $As_4S_4/ZnS/Fe_3O_4$ with FA and Alb at concentrations: 0.5, 1, and 2 μM and novel: bortezomib (BTZ; 0.25, 0.5, 1, 2, and 4 nM), lenalidomide (LEN; 10, 25, and 50 μM), and pomalidomide (POM; 10, 25, and 50 μM) or conventional: doxorubicin (DOX; 10, 25, 50, 100, 250, and 500 nM), dexamethasone (DEX; 100, 250, 500, and 1000 nM), and melphalan (MEL; 0.5, 1, 2, 2.5, 5, 10, and 20 μM) anti-MM agents was synergistic, the CalcuSyn software (Biosoft, Ferguson, MO, USA) was used to perform isobologram analysis and calculate the combination index (CI), according to the Chou-Talalay method. When CI=1, the effects are considered additive, whereas CI<1 indicates synergism and CI >1 indicates antagonism.

Flow cytometric analysis of apoptosis and cell cycle

Detection of apoptosis

Apoptotic cells of $As_4S_4/ZnS/Fe_3O_4$-, $As_4S_4/ZnS/Fe_3O_4$ with FA-, and $As_4S_4/ZnS/Fe_3O_4$ with FA and Alb (1, 2, and 4 μM)-treated, and compared to control MM cells, were quantified using the Annexin V-FITC/PI apoptosis assay. Briefly, both suspension and adherent cells were collected and washed twice with cold PBS. Cells (3×10^5) were resuspended in 100 μl of manufacturer-supplied 1X binding buffer and mixed with 5 μl of Annexin V-FITC (BD Biosciences Pharmingen) and 5 μl of propidium iodide (PI). After 30 min incubation in the dark at room temperature, cells were analyzed by a FACS Canto II flow cytometer (Becton Dickinson) using a 96-well format.

Cytofluorimetric analysis of mitochondrial potential

The mitochondrial membrane potential of $As_4S_4/ZnS/Fe_3O_4$-, $As_4S_4/ZnS/Fe_3O_4$ with FA-, and $As_4S_4/ZnS/Fe_3O_4$ with FA and Alb (1, 2, and 4 μM)-treated, and compared to control MM cells, was studied using the JC-1 fluorescent probe, a mitochondria-selective probe that forms aggregates in normal polarized mitochondria resulting in an orange emission. Its monomeric forms, present in cells with depolarized mitochondrial membranes, emit only green fluorescence. Briefly, 3×10^5 cells were incubated in 200 μl of PBS/0.2% BSA containing 4 μM of JC-1 (Molecular Probes, Eugene, OR) for 30 min at 37 °C. After 30 min incubation in the dark at 37 °C, cells were analyzed using a FACS Canto II flow cytometer (Becton Dickinson) using a 96-well format.

Cell cycle analysis

Changes in cell cycle status of $As_4S_4/ZnS/Fe_3O_4$-, $As_4S_4/ZnS/Fe_3O_4$ with FA-, and $As_4S_4/ZnS/Fe_3O_4$ with FA and Alb (1, 2, and 4 μM)-treated cells were determined by flow cytometric measurement of DNA content of nuclei labeled with PI. Briefly, MM cells (3×10^5) were collected, washed twice with cold PBS, and incubated in 0.05% Triton X-100 and 15 μl RNA-se A (10 mg/ml) for 20 min at 37 °C. Then cells were cooled and incubated on ice for at least 10 min before PI (50 μg/ml) was added. Finally, the stained cells were analyzed using a FACS Canto II flow cytometer (Becton Dickinson) using a 96-well format.

Flow cytometry measurements and data analysis

Flow cytometry measurements were performed with a FACS Canto II flow cytometer equipped with 488 nm excitation laser. All fluorochromes were excited with a 488 nm laser, and data were collected through a respective photomultiplier as follows: Annexin V-FITC and PI (FL1, FL3); JC-1 (FL1, FL2, ratio FL2/FL1); and cell cycle (log FL3, sub G1; lin FL2, DNA cell cycle histogram; FL3 peak versus integral for doublets discrimination). Forward/side light scatter characteristic was used to exclude the cell debris from the analysis. For each analysis, 1-2 x 10^4 cells were acquired for analysis. Data were analyzed with De Novo FCS Express software (De Novo software, Los Angeles, CA, USA).

Co-culture model by CFSE assay

To distinguish MM cells in co-culture models, MM cells were labeled with carboxyfluorescein diacetate succinimidyl ester (CFSE), fluorescent cytoplasmic dye that is equally diluted between daughter cells during cell division, and seeded on the unlabeled bone marrow stromal HS-5 cells for 24 and 48 h in culture. Changes in mean fluorescence intensity of CFSE signal were used as a surrogate marker of cell proliferation. Briefly, MM cells were labeled with 1 μM CFSE (Molecular Probes, Eugene, OR, USA) for 10 minutes at 37°C in serum-free RPMI in the dark. The reaction was stopped by adding RPMI 1640 medium supplemented with 2% FBS, and cells were washed 3 times with 10% FBS RPMI 1640. Cells were then seeded, either alone or together with unlabeled bone marrow stromal HS-5 cells seeded 24 h prior to co-culture, followed by $As_4S_4/ZnS/Fe_3O_4$, $As_4S_4/ZnS/Fe_3O_4$ with FA, and $As_4S_4/ZnS/Fe_3O_4$ with FA and Alb (0.125, 0.25, 0.5, 1, and 2 μM) treatment.

Side population analysis by Hoechst 33342 assay

The cells were labelled with Hoechst 33342 dye using the method described by Goodell et al. with modifications. Cells were washed in pre-warmed RPMI 1640 with 2% FBS and 10 mmol/L Hepes buffer (Life Technologies, Carlsbad, CA, USA), and then resuspended in RPMI 1640 with 2% FBS and 10 mmol/L Hepes buffer containing 5 μg/mL of Hoechst 33342 dye (Molecular probes, Eugene, OR); cells were then incubated for 90 min at 37 °C with intermittent shaking. As a

negative control, cells were preincubated with 50 μmol/L reserpine, an ABC transporter inhibitor. At the end of the incubation, cells were washed with ice-cold PBS containing 2% FBS and 10 mmol/L Hepes buffer. To gate on only viable cells, 7-AAD (Molecular probes, Eugene, OR, USA; final concentration = 1 μg/mL) in PBS with 2% FBS and 10 mmol/L Hepes buffer was added to the cells. The cells were analyzed by a FACS Aria Special Sorter equipped with UV laser (Becton Dickinson, Mountain View, CA, USA). The Hoechst 33342 dye was excited at 357 nm, followed by dual-wavelength fluorescence analysis (blue, 402-446 nm; 650-670 nm).

Western immunoblotting analysis

After treatment with $As_4S_4/ZnS/Fe_3O_4$, $As_4S_4/ZnS/Fe_3O_4$ with FA, or $As_4S_4/ZnS/Fe_3O_4$ with FA and Alb (1, 2, and 4 μM), cells were washed twice with ice-cold PBS, resuspended in 100 μl of ice-cold cell lysis buffer (1% Nonidet P-40, 50 mM Tris, pH 7.4, 150 mM NaCl, 2 mM EDTA, 2 mM PMSF, 1 mM sodium vanadate, 1 mM sodium fluoride and 1 x protease mixture), and then incubated on ice for 20 min. After centrifugation for 10 min at 10,000 x g, supernatants were collected. Protein concentrations were measured using a Bradford protein assay kit. Equivalent amounts of protein (20 μg) were mixed with 4X SDS-PAGE sample buffer (Invitrogen, Carlsbad, CA) and 10X reducing agent (0.5 M dithiotreitol, Invitrogen, Carlsbad, CA) and separated by SDS-PAGE. Resolved proteins were transferred to a nitrocellulose membrane (Bio-Rad) using a semi-dry transfer system. Membranes were blocked for 1 h at room temperature with 5% non-fat dry milk in Tris–buffered saline (TBS), pH 7.4, containing 1% Tween 20 (TBS-T), followed by overnight incubation at 4 °C with 1:1000 dilution of the respective primary antibodies: using anti-caspase-3, -caspase-7, -caspase-8, -caspase-9, -Bax, -Apaf-1 (apoptotic protease activating factor-1), -PARP, -c-Myc, and -XIAP (x-linked inhibitor of apoptosis), -ATM, -p-ATM, -ATR, -p-ATR, -Chk1, -Chk2, -p-Chk2, -Cdc-2, -p-Cdc-2, -Cyclin B1, -histone H2AX (H2AX), -p-histone H2AX (p-H2AX), -histone H3 (H3), -p-histone H3 (p-H3), -ERK1/2, -p-ERK1/2, -STAT3, -p-JNK1/2/3, -c-Myc, -mTOR, -p-mTOR, and -GAPDH (Cell Signaling Technology). Membranes were washed in TBS-T, and then incubated with horseradish peroxidase-conjugated goat anti-mouse or anti-rabbit secondary antibodies (Cell Signaling Technology, Danvers, MA, USA) for 1 h at room temperature. Proteins were visualized with enhanced chemiluminescent (ECL) system (Amersham Bioscience, Little Chalfont, UK).

SUPPLEMENT FIGURE LEGENDS

Supplementary Figure S1: Cytotoxic effects of As4S4/ZnS/Fe3O4 (1:4:1), As4S4/ZnS/Fe3O4 (1:4:1) with FA, and As4S4/ZnS/Fe3O4 (1:4:1) with FA and Alb in MM cells. (A) MM cell lines (MM.1S, OPM-1, OPM-2, RPMI-S, RPMI-LR5, RPMI-DOX40, RPMI-MR20, KMS11, KMS34, U266, and L363) were treated with all 3NPs in the concentrations: 0.125, 0.25, 0.5, 1, 2, 4, and 8 μM for 24 and 72 h and cell survival was assessed by MTT assay. (B) The EC_{50} values of all 3NPs were determined in MM cells for 24 and 72 h by the CalcuSyn software. Each treatment with a specific concentration of NPs was done in triplicate. The data presented are mean ± standard deviation, expressed as survival/viability relative to untreated controls.

Supplementary Figure S2: Cytotoxic effects of As4S4/ZnS/Fe3O4 (1:4:1), As4S4/ZnS/Fe3O4 (1:4:1) with FA, and As4S4/ZnS/Fe3O4 (1:4:1) with FA and Alb in leukemia and solid cancer cell lines. (A) Acute promyelocytic leukemia cell lines (HL60, HL60-MDR1, and HL60/PLB-ABCG2) and adenocarcinoma Caco-2 and breast cancer MCF-7 cells were treated with all 3NPs in the concentrations: 0.125, 0.25, 0.5, 1, 2, 4, and 8 μM for 24, 48, and 72 h and cell survival was assessed by MTT assay. (B) The EC_{50} values of 3NPs were determined for 24, 48, and 72 h by the CalcuSyn software. Each treatment with a specific concentration of NPs was done in triplicate. The data presented are mean ± standard deviation, expressed as survival/viability relative to untreated controls.

Supplementary Figure S3: Cytotoxic effects of As4S4/ZnS/Fe3O4 (1:4:1), As4S4/ZnS/Fe3O4 (1:4:1) with FA, and As4S4/ZnS/Fe3O4 (1:4:1) with FA and Alb in healthy peripheral blood mononuclear cells. (A) Freshly isolated peripheral blood mononuclear cells (MNCs) from healthy donors (HD; N = 13) were treated with all 3NPs in the concentrations: 0-8 μM for 48 h and cell survival was assessed by MTT assay. (B) The EC_{50} values of all 3NPs were determined for 48 by the CalcuSyn software. Each treatment with a specific concentration of NPs was done in triplicate. The data presented are mean ± standard deviation, expressed as survival/viability relative to untreated controls.

Supplementary Figure S4: As4S4/ZnS/Fe3O4 (1:4:1), As4S4/ZnS/Fe3O4 (1:4:1) with FA, and As4S4/ZnS/Fe3O4 (1:4:1) with FA and Alb trigger apoptosis in MM cells. MM.1S, RPMI-S, OPM-1, and OPM-2 cells were cultured with all 3NPs at 1, 2, and 4 μM for 24 h. (A) Depletion of mitochondrial membrane potential in 3NPs-treated MM cell lines was quantified by straining with the fluorescent JC1 dye by increased levels of JC1 monomers, and analyzed by a FACS Canto II flow cytometer. Data are from three independent experiments. (B) Effects of all 3NPs on induction of apoptosis and necrosis were evaluated with Annexin V-FITC and PI staining. Percentages of early apoptotic (Annexin V-FITC+/PI−), late apoptotic (Annexin V+/PI+/−) and necrotic (Annexin V+/PI+) cells were analyzed by a FACS Canto II flow cytometer. Data are from two independent experiments, presented as mean ± standard deviation. Significant differences between treatments and control were identified by t-test with $^{*}p < 0.05$, $^{**}p < 0.01$, and $^{***}p < 0.001$.

Supplementary Figure S5: As4S4/ZnS/Fe3O4 (1:4:1), As4S4/ZnS/Fe3O4 (1:4:1) with FA, and As4S4/ZnS/Fe3O4 (1:4:1) with FA and Alb trigger cell cycle arrest in MM cells. MM.1S, RPMI-S, OPM-1, and OPM-2 cells were cultured with all 3NPs at 1, 2, and 4 μM for 48 h. The distribution of cells in G_0/G_1, S, and G_2/M phase were measured by a FACS Canto II flow cytometer and analyzed by *De Novo* FCS Express software. Data are representative of three independent experiments, presented as mean ± standard deviation. Significant differences between treatments and control were identified by t-test with $^{*}p < 0.05$, $^{**}p < 0.01$, and $^{***}p < 0.001$.

Supplementary Figure S6: As4S4/ZnS/Fe3O4 (1:4:1), As4S4/ZnS/Fe3O4 (1:4:1) with FA, and As4S4/ZnS/Fe3O4 (1:4:1) with FA and Alb inhibit proliferation of MM cells alone and in co-culture with bone marrow stromal cells (BMSC). Carboxyfluorescein diacetate succinimidyl ester (CFSE)-stained MM.1S, RPMI-S and OPM-1 cells, alone or in co-culture with BMSC HS-5 cells, were treated with all 3NPs at 1, 2, and 4 μM for 24 h. Non-viable MM (CFSE+/PI+) cells were determined by PI staining and analyzed by a FACS Canto II flow cytometer. (A) Fluorescence intensity of gated $CFSE^{+}PI^{-}$-stained MM cells is shown as a function of all 3NPs concentration (1, 2, and 4 μM). (B) The ratio of fraction affected (non-viable cells) MM cells triggered by 3NPs relative to untreated controls is shown as a function of all 3NPs concentration (1, 2, and 4 μM). Each treatment with a specific concentration of NPs was done in

triplicate from two independent experiments. The data are presented as mean ± standard deviation. Significant differences between treatments and control were identified by t-test with $*p < 0.05$, $**p < 0.01$, and $***p < 0.001$.

Supplementary Figure S7: $As_4S_4/ZnS/Fe_3O_4$ (1:4:1), $As_4S_4/ZnS/Fe_3O_4$ (1:4:1) with FA, and $As_4S_4/ZnS/Fe_3O_4$ (1:4:1) with FA and Alb enhance the effect of anti-MM agents *in vitro*. MM.1S, RPMI-S, OPM-1, and OPM-2 cells were cultured with all 3NPs in combination with novel anti-MM drugs: bortezomib (velcade; BTZ), lenalidomide (LEN) and pomalidomide (POM) and conventional anti-MM drugs: doxorubicin (DOX), dexamethasone (DEX) and melphalan (MEL) for 24 h. Cell viability was then assessed using the MTT assay. Fractions-affected (Fa – ratio of number of nonviable MM cells/total number of MM cells) cells was visualized in a color-coded format and compared to treatment with each drug alone. Isobologram analysis was performed to calculate the combination index (CI) for each combination by the Chou–Talalay method. The x-axis corresponds to the fractional effect at various combination doses and the y-axis represents the CI; $CI < 1$ indicates drug synergy, whereas $CI = 1$ is considered additive, and $CI > 1$ indicates antagonism. All experiments were performed in triplicate. Data represent mean (± standard deviation) of triplicate cultures.
