# Supplementary material for: In vitro and ex vivo anti-myeloma effects of nanocomposite As4S4/ZnS/Fe3O4
Source: Sci Rep. 2022 Oct 26;12:17961. doi: 10.1038/s41598-022-22672-5 (PMC9606304; doi:10.1038/s41598-022-22672-5)
Supplement: Supplementary file 9 — Supplementary Information 9. [file 41598_2022_22672_MOESM9_ESM.pdf]

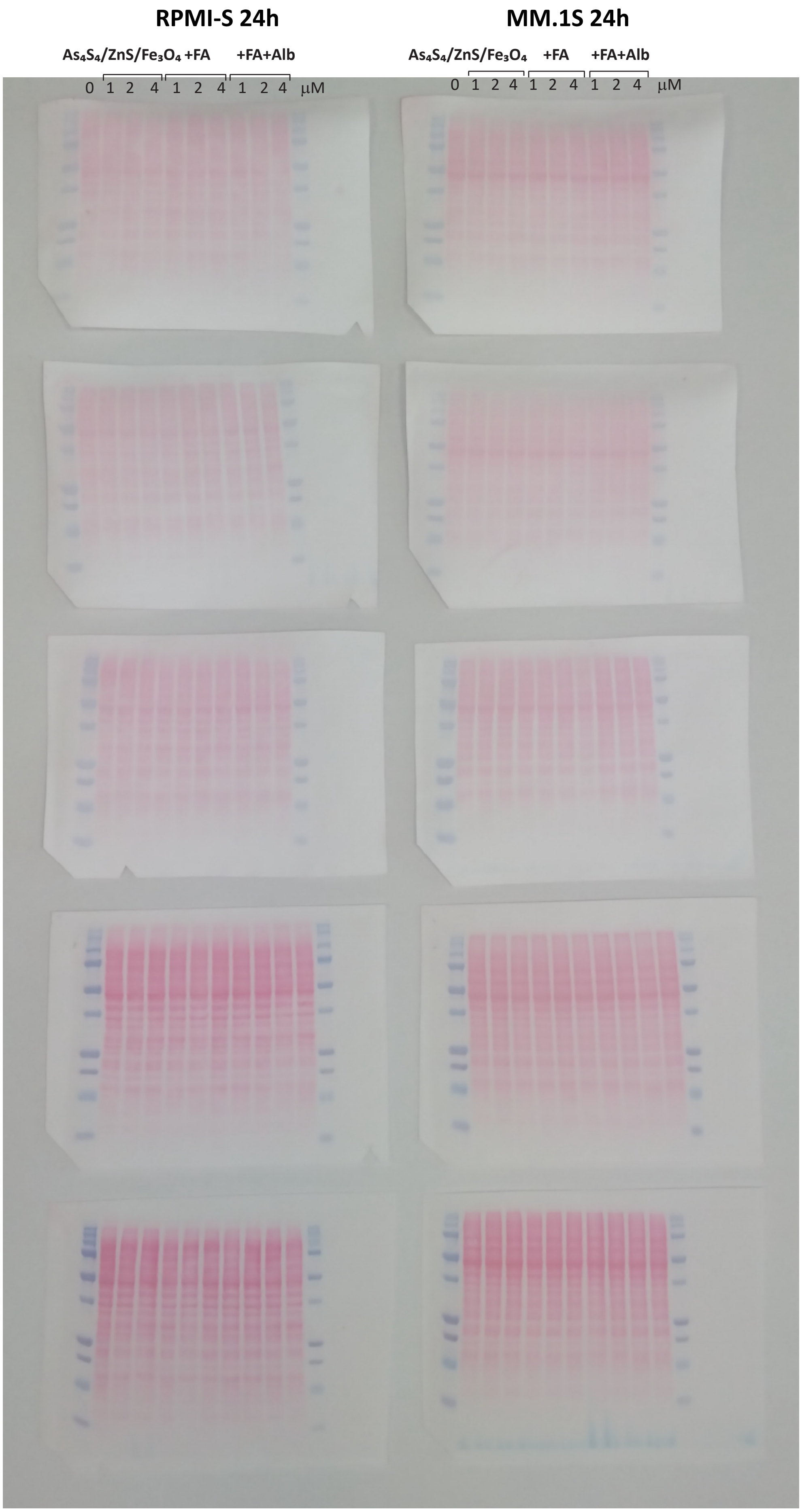
RPMI-S 24h
MM.1S 24h
$As_4S_4/ZnS/Fe_3O_4$ +FA +FA+Alb
0 1 2 4 1 2 4 1 2 4 μM
$As_4S_4/ZnS/Fe_3O_4$ +FA +FA+Alb
0 1 2 4 1 2 4 1 2 4 μM

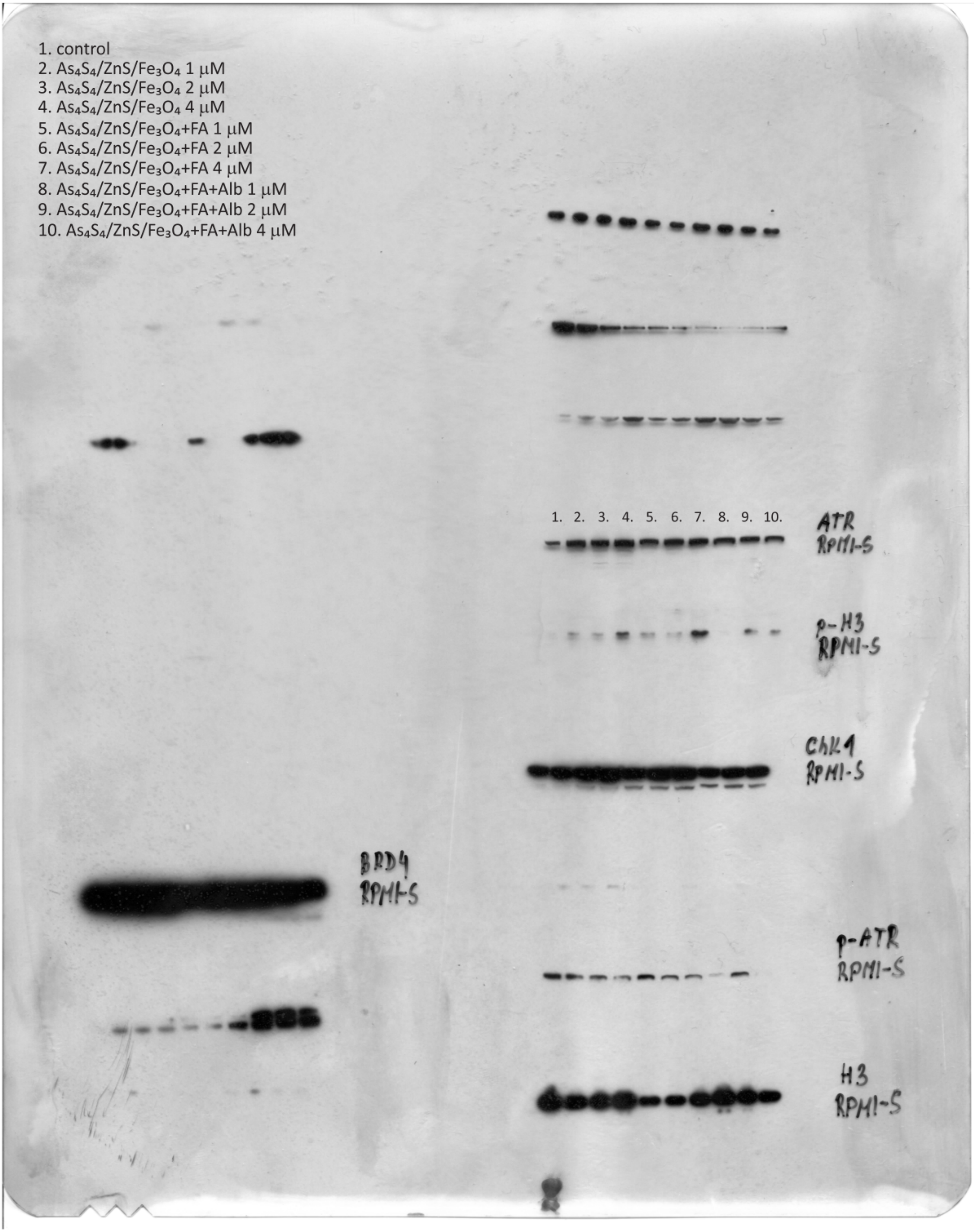
1. control
2. $As_4S_4/ZnS/Fe_3O_4$ 1 μM
3. $As_4S_4/ZnS/Fe_3O_4$ 2 μM
4. $As_4S_4/ZnS/Fe_3O_4$ 4 μM
5. $As_4S_4/ZnS/Fe_3O_4$+FA 1 μM
6. $As_4S_4/ZnS/Fe_3O_4$+FA 2 μM
7. $As_4S_4/ZnS/Fe_3O_4$+FA 4 μM
8. $As_4S_4/ZnS/Fe_3O_4$+FA+Alb 1 μM
9. $As_4S_4/ZnS/Fe_3O_4$+FA+Alb 2 μM
10. $As_4S_4/ZnS/Fe_3O_4$+FA+Alb 4 μM
1. 2. 3. 4. 5. 6. 7. 8. 9. 10.
ATR RPMI-S
p-H3 RPMI-S
ChK1 RPMI-S
BRD4 RPMI-S
p-ATR RPMI-S
H3 RPMI-S

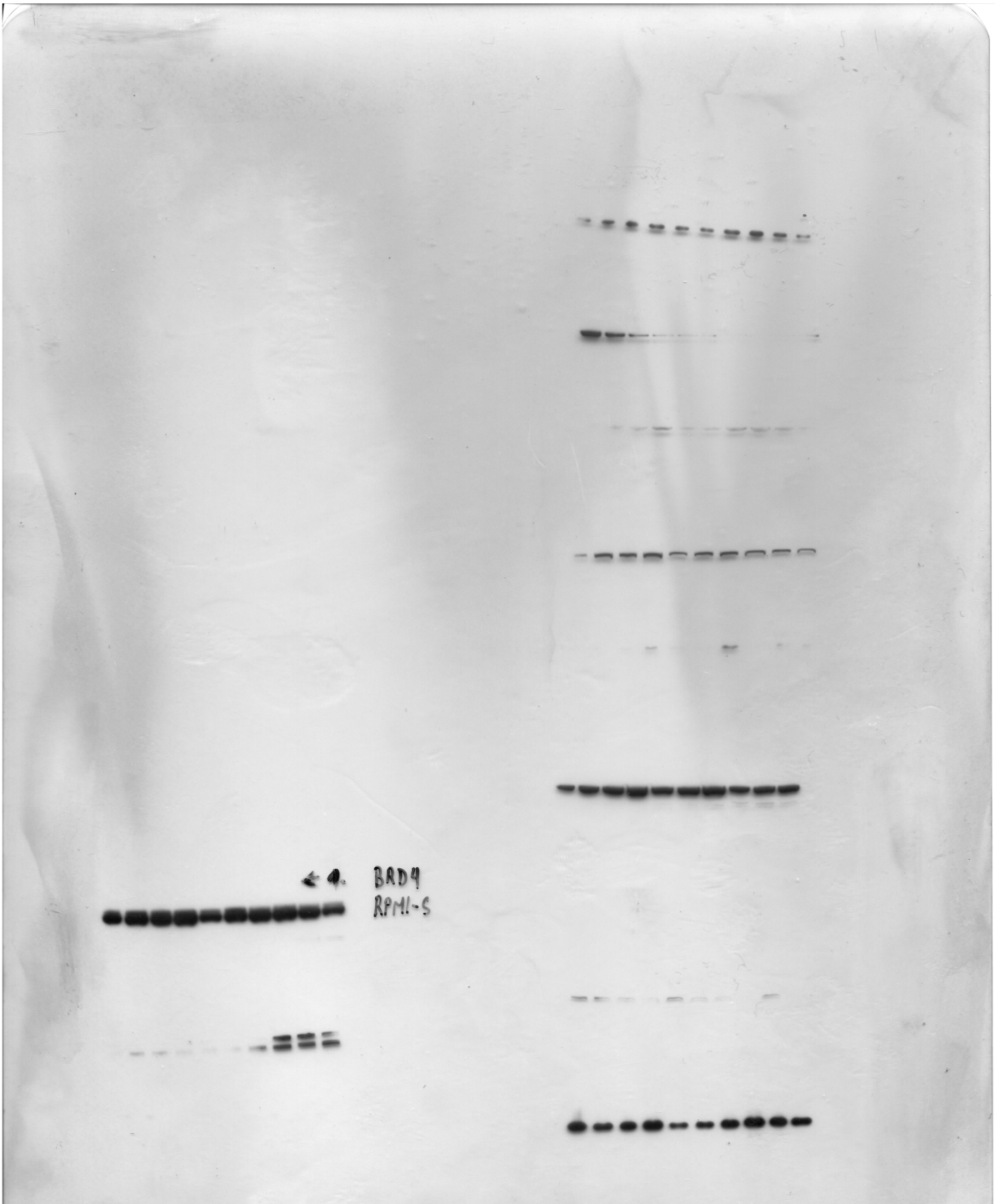
BRD4
RPMI-S

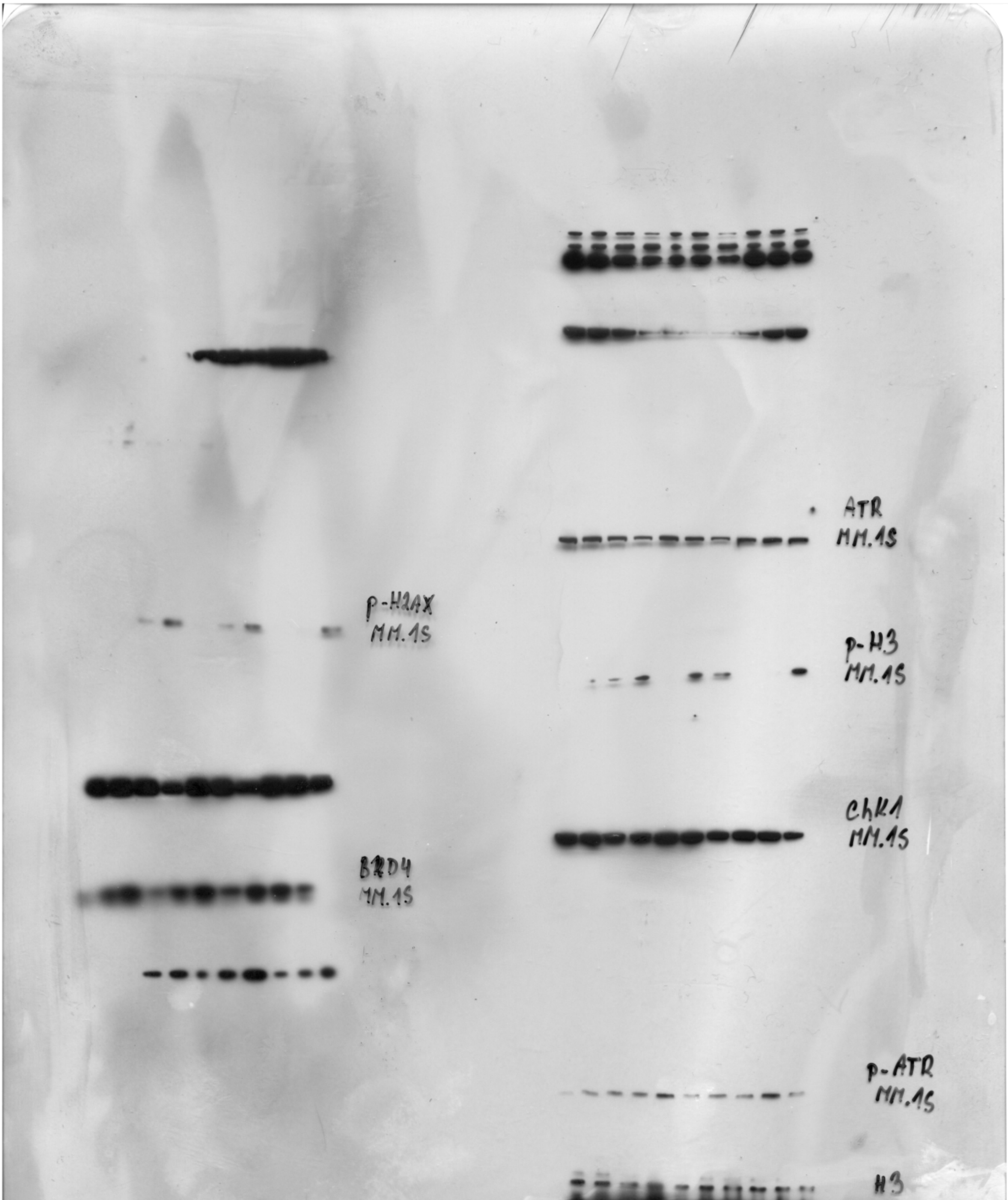

p-H2AX
MM.1S
BRD4
MM.1S
ATR
MM.1S
p-H3
MM.1S
ChK1
MM.1S
p-ATR
MM.1S
H3
MM.1S

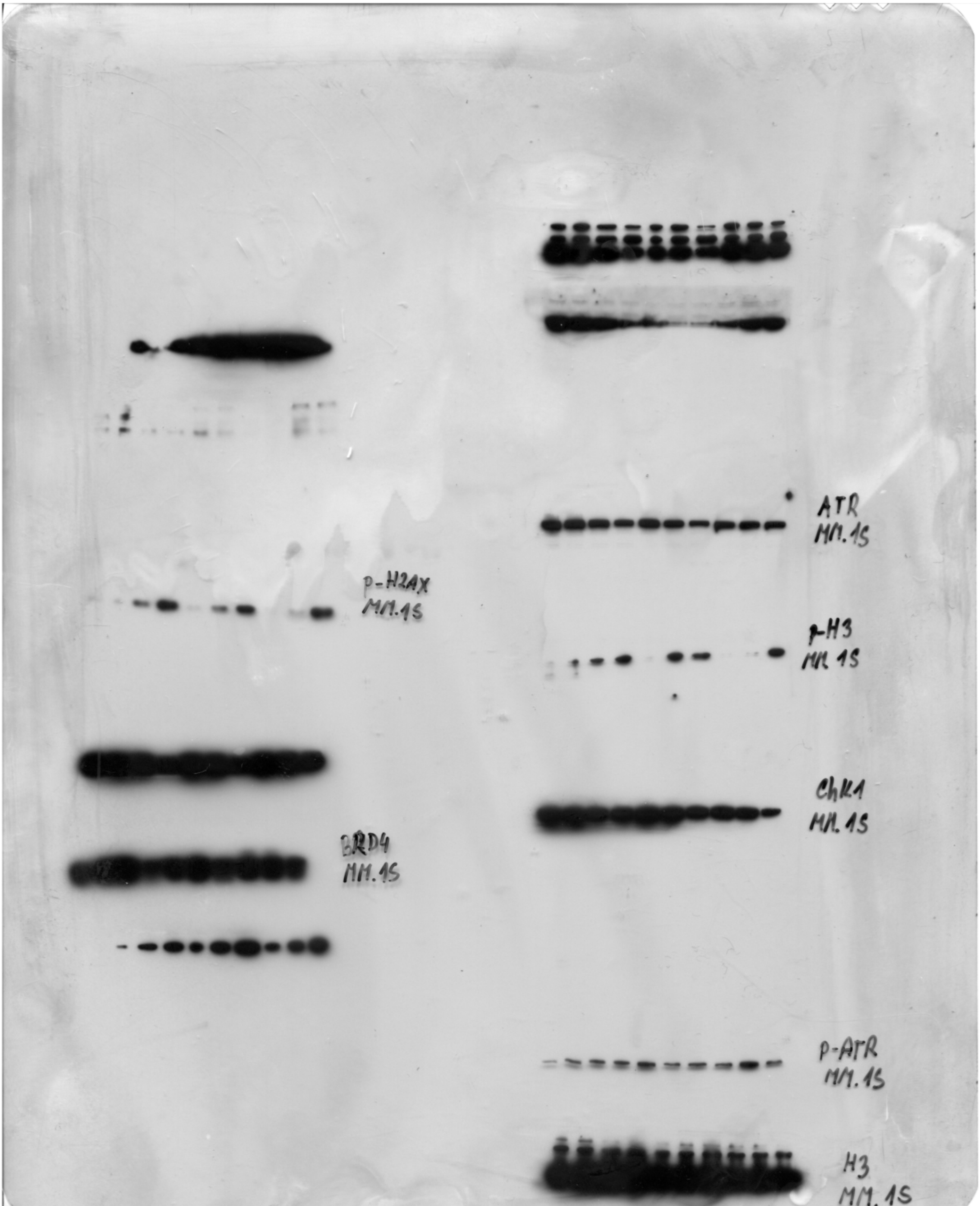
p-H2AX
MM.1S
BRD4
MM.1S
ATR
MM.1S
p-H3
MM.1S
ChK1
MM.1S
p-ATR
MM.1S
H3
MM.1S

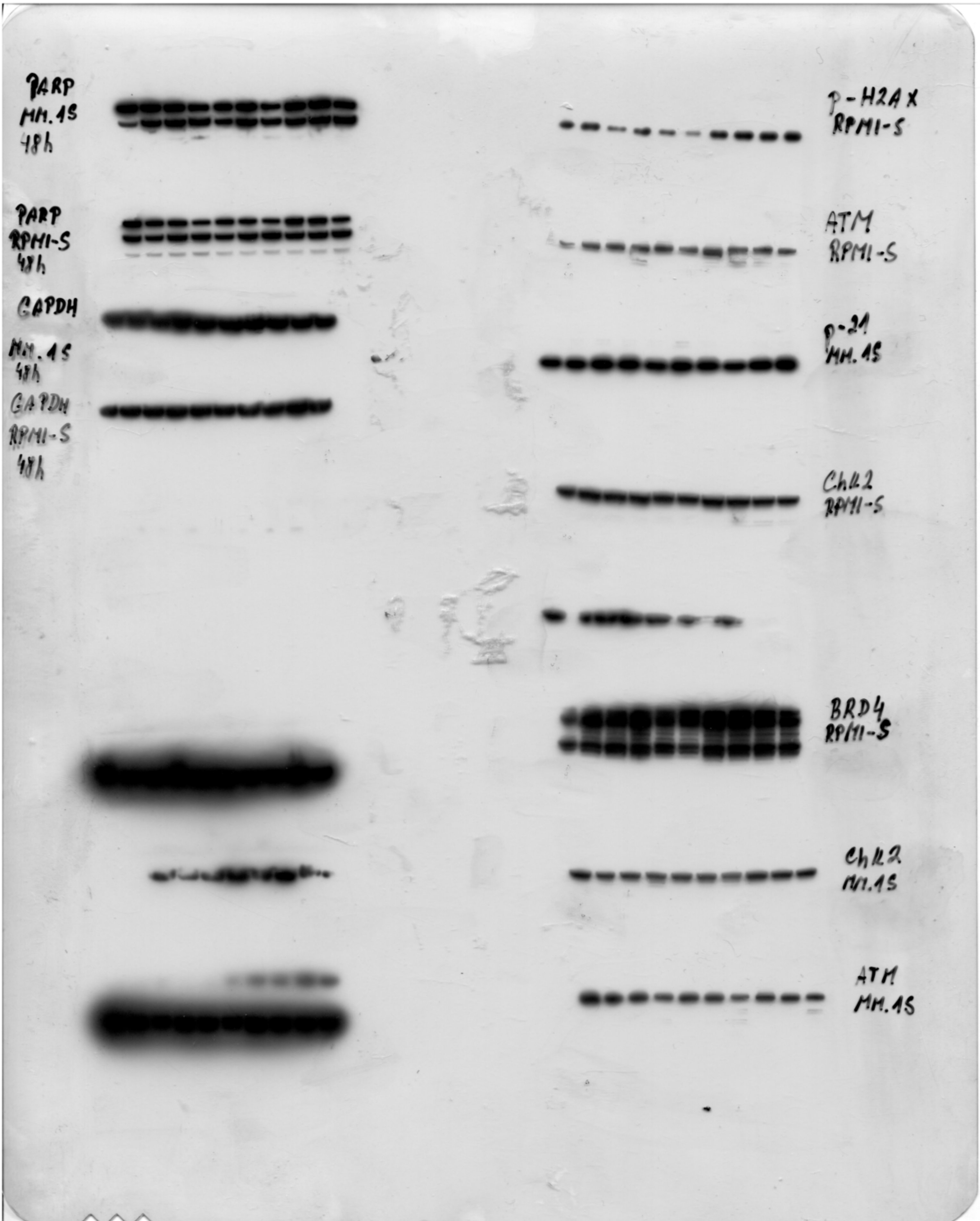
PARP
MM.1S
48h
PARP
RPMI-S
48h
GAPDH
MM.1S
48h
GAPDH
RPMI-S
48h
p-H2AX
RPMI-S
ATM
RPMI-S
p-21
MM.1S
Chk2
RPMI-S
BRD4
RPMI-S
Chk2
MM.1S
ATM
MM.1S

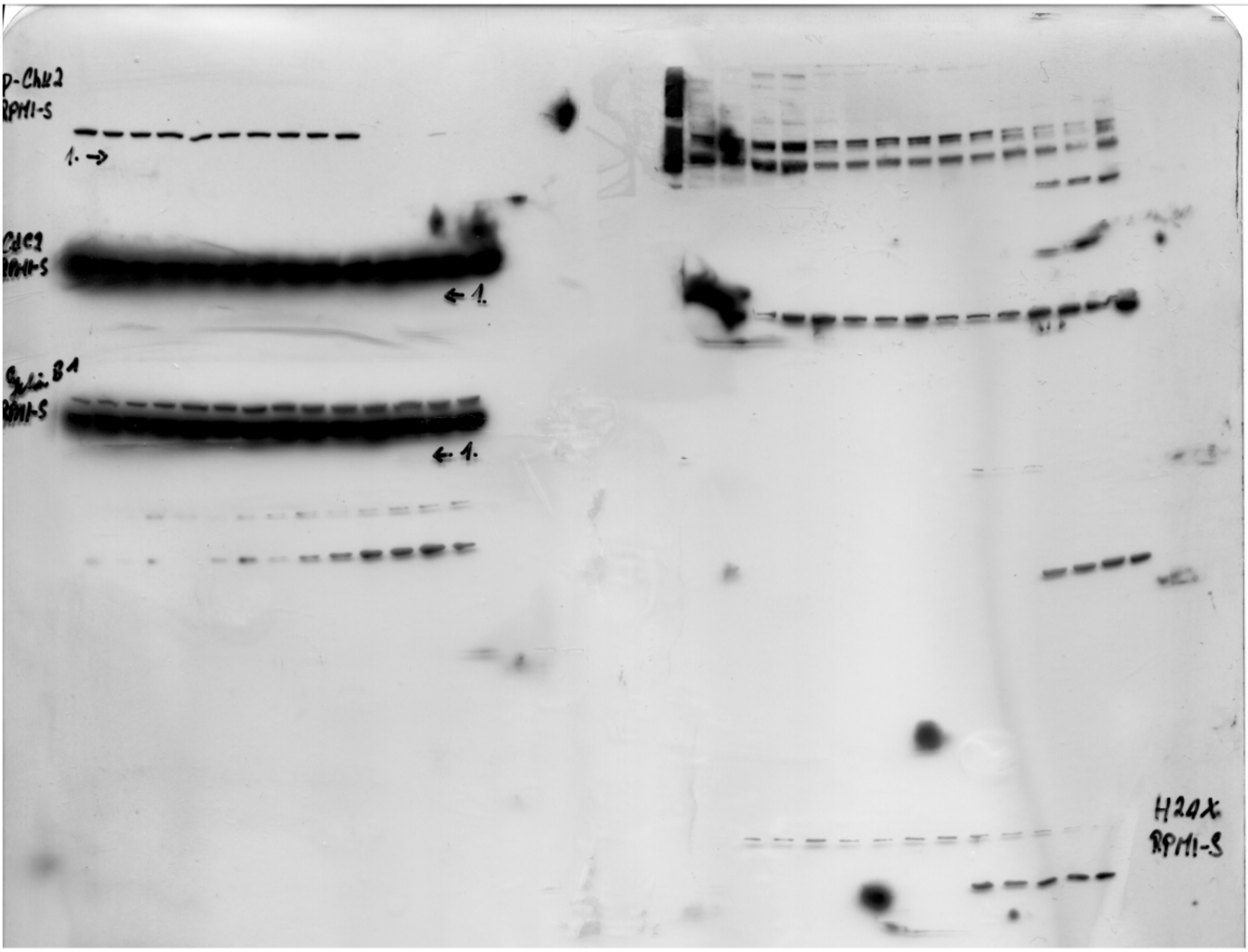

p-Chk2
RPMI-S
1. →
Cdc2
RPMI-S
← 1.
Cyclin B1
RPMI-S
← 1.
H2AX
RPMI-S


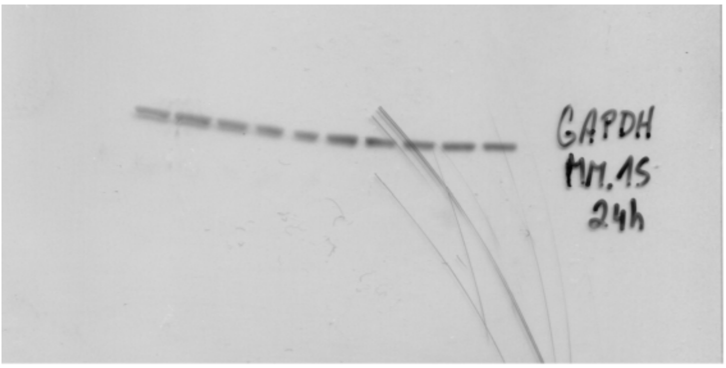

GAPDH
MM.1S
24h

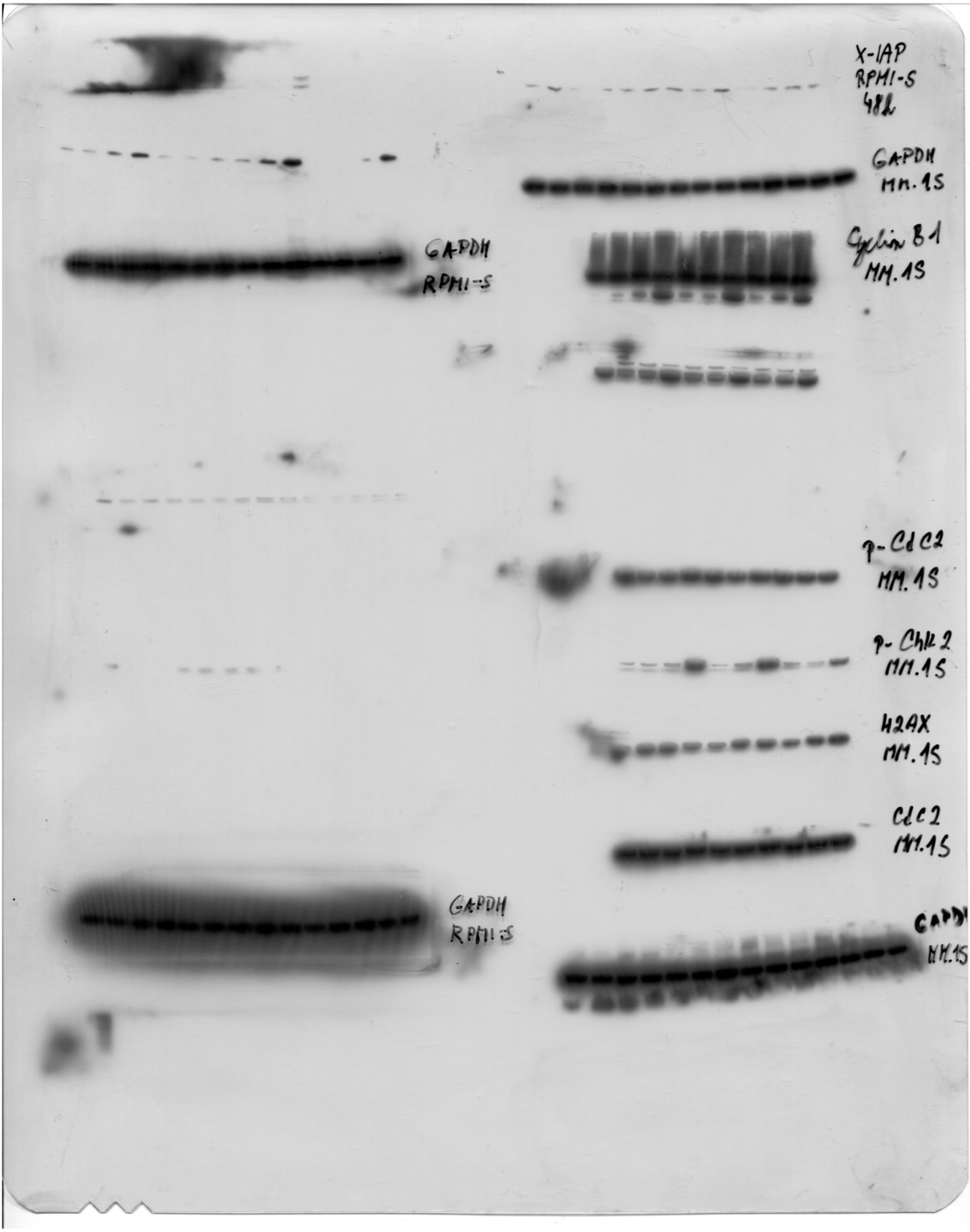
X-IAP
RPMI-S
48h
GAPDH
MM.1S
GAPDH
RPMI-S
Cyclin B1
MM.1S
p-Cdc2
MM.1S
p-Chk2
MM.1S
H2AX
MM.1S
Cdc2
MM.1S
GAPDH
RPMI-S
GAPD
MM.1S

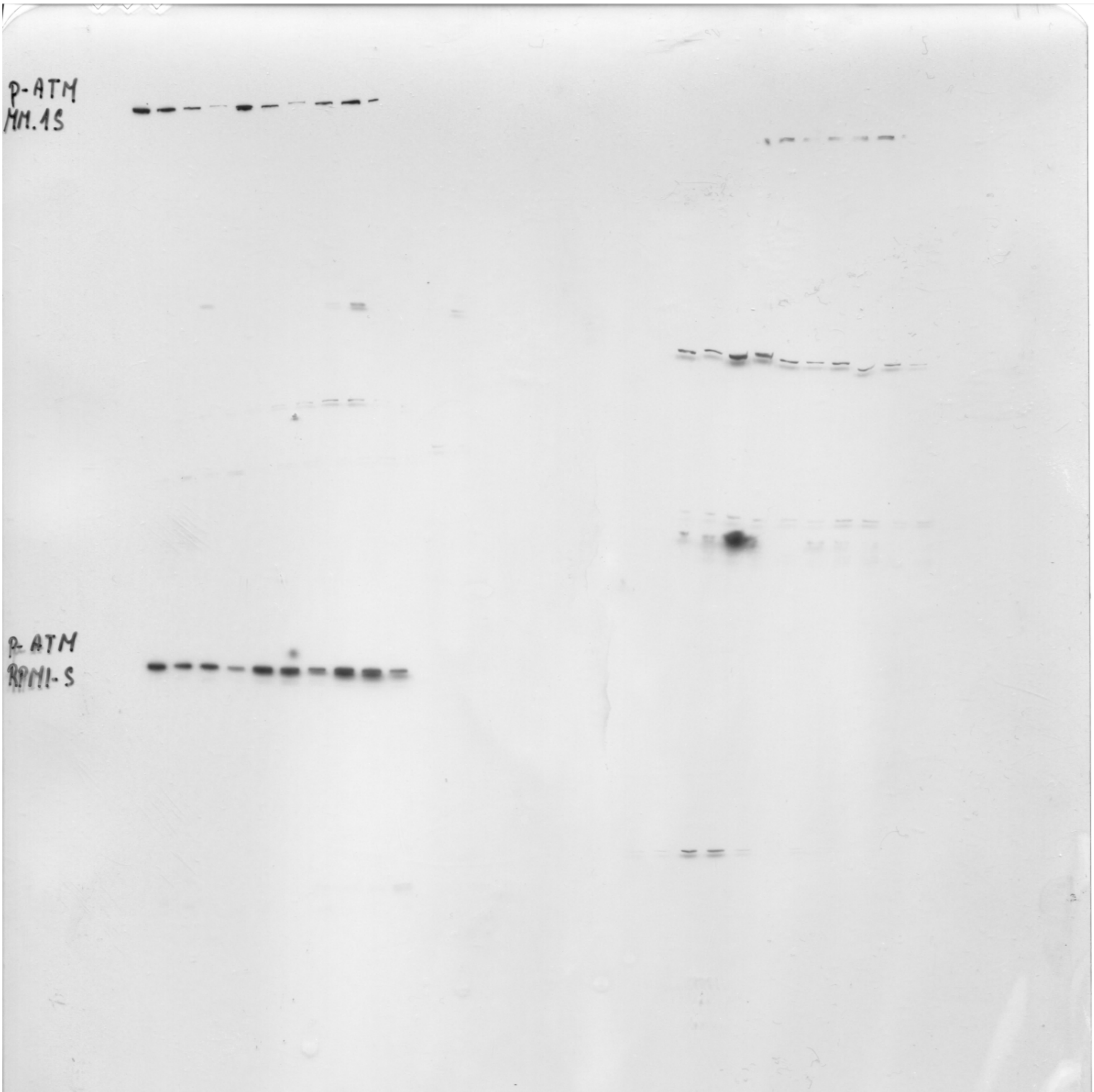
p-ATM
MM.1S
p-ATM
RPMI-S
2

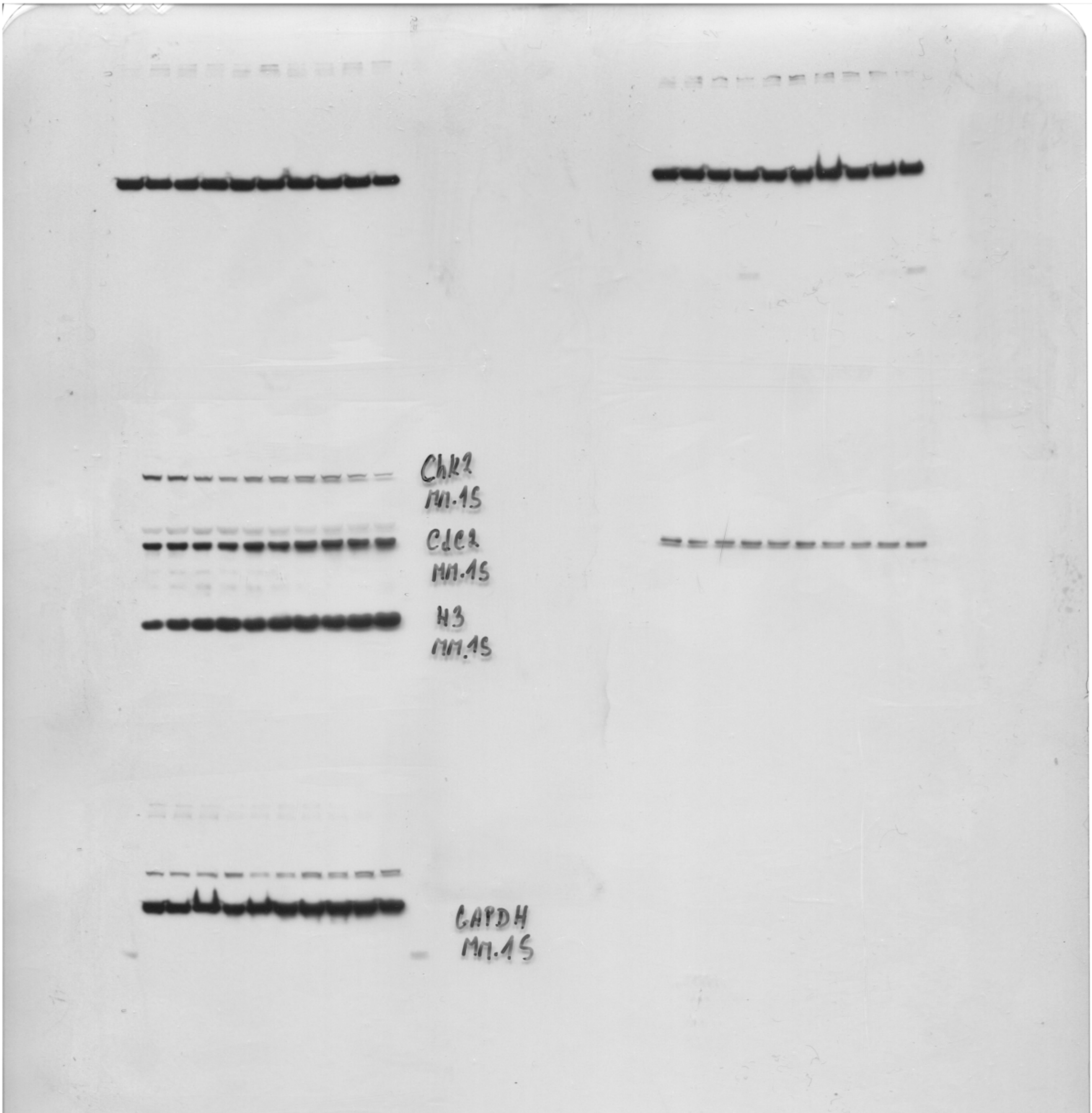
Chk2
MM.1S
Cdc2
MM.1S
H3
MM.1S
GAPDH
MM.1S

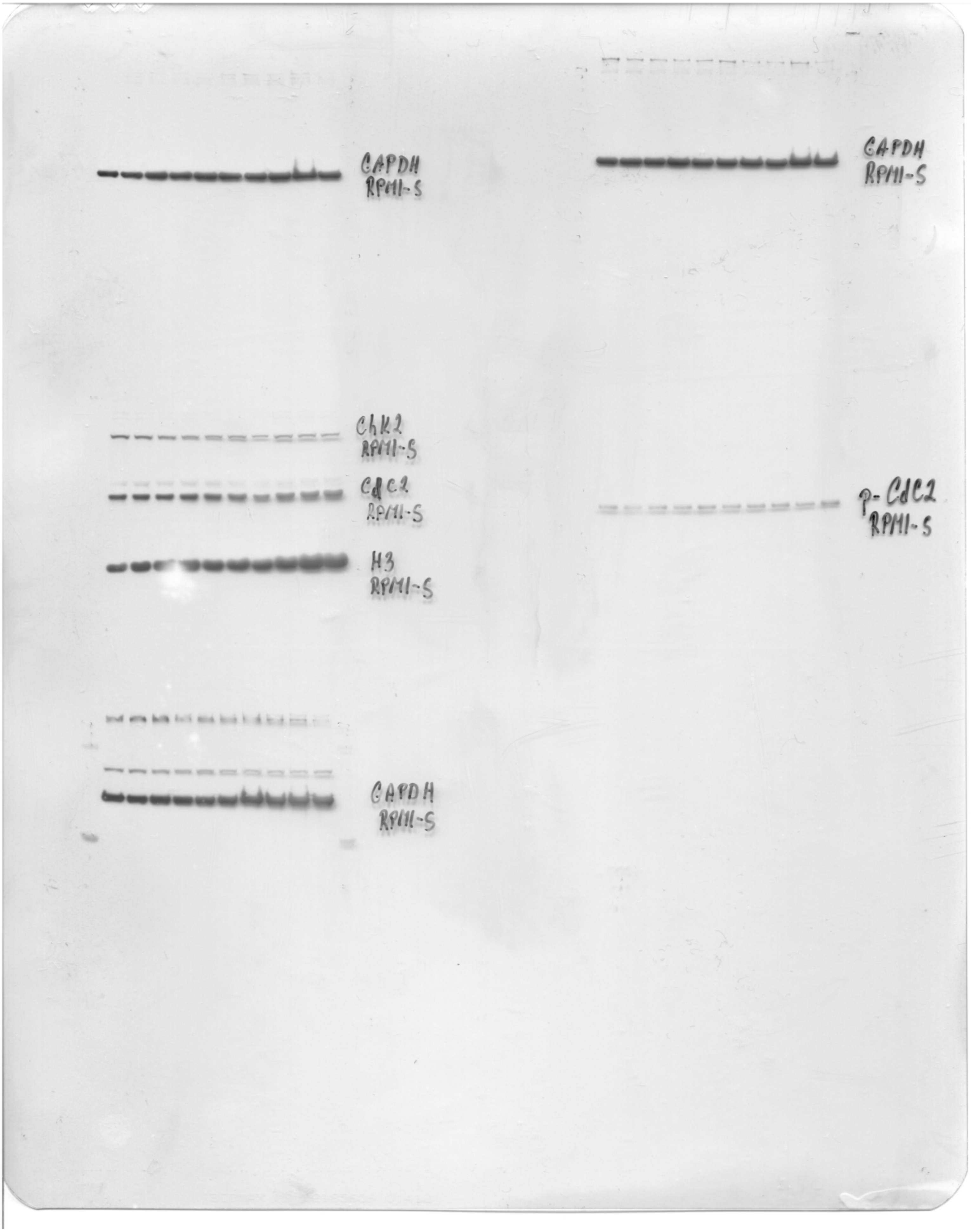
GAPDH RPMI-S
ChK2 RPMI-S
CdC2 RPMI-S
H3 RPMI-S
GAPDH RPMI-S
GAPDH RPMI-S
p-CdC2 RPMI-S

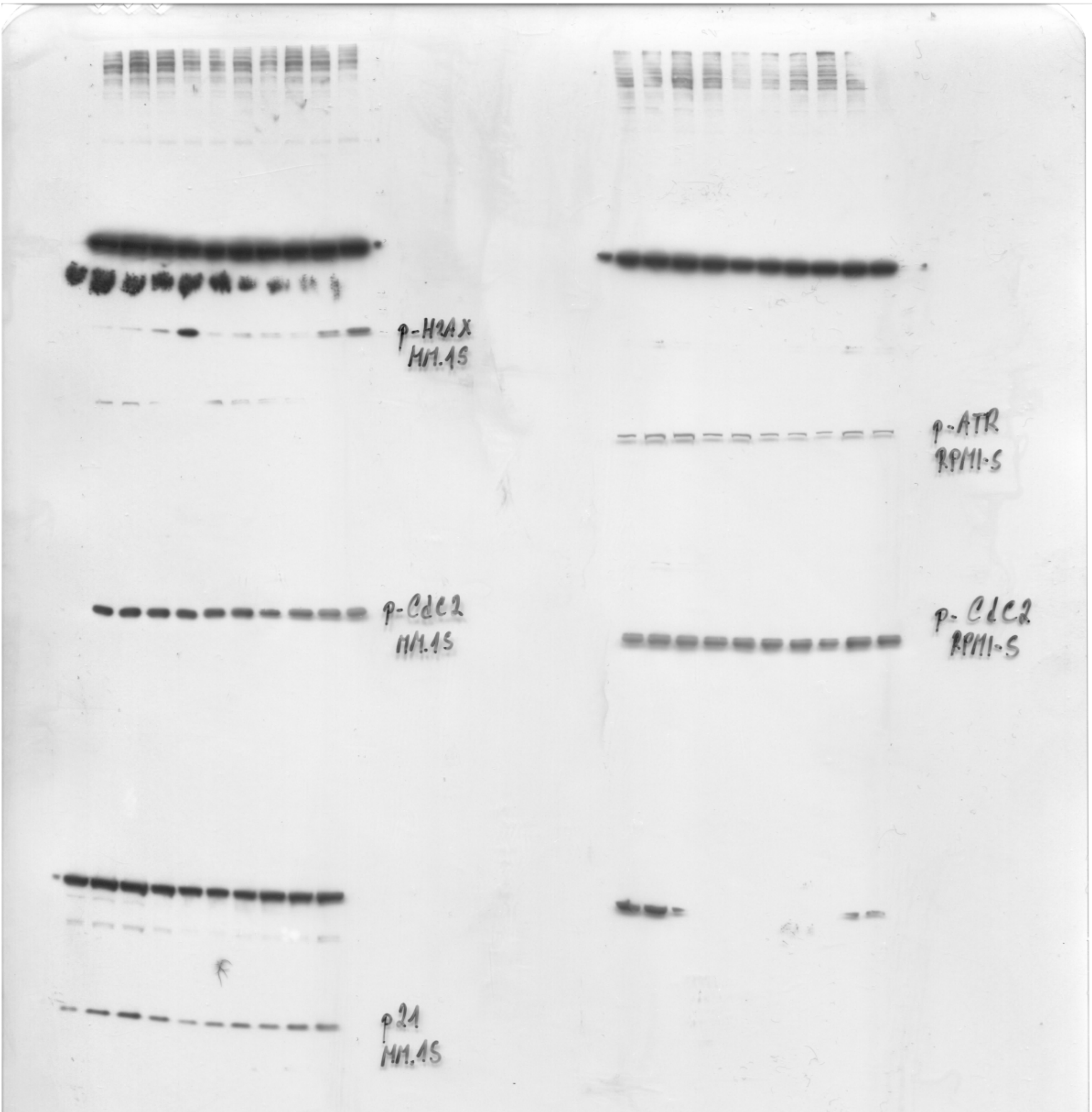
p-H2AX
MM.1S
p-ATR
RPMI-S
p-Cdc2
MM.1S
p-Cdc2
RPMI-S
p21
MM.1S
