# Supplementary material for: In vitro and ex vivo anti-myeloma effects of nanocomposite As4S4/ZnS/Fe3O4
Source: Sci Rep. 2022 Oct 26;12:17961. doi: 10.1038/s41598-022-22672-5 (PMC9606304; doi:10.1038/s41598-022-22672-5)
Supplement: Supplementary file 10 — Supplementary Information 10. [file 41598_2022_22672_MOESM10_ESM.pdf]

RPMI-S 48h

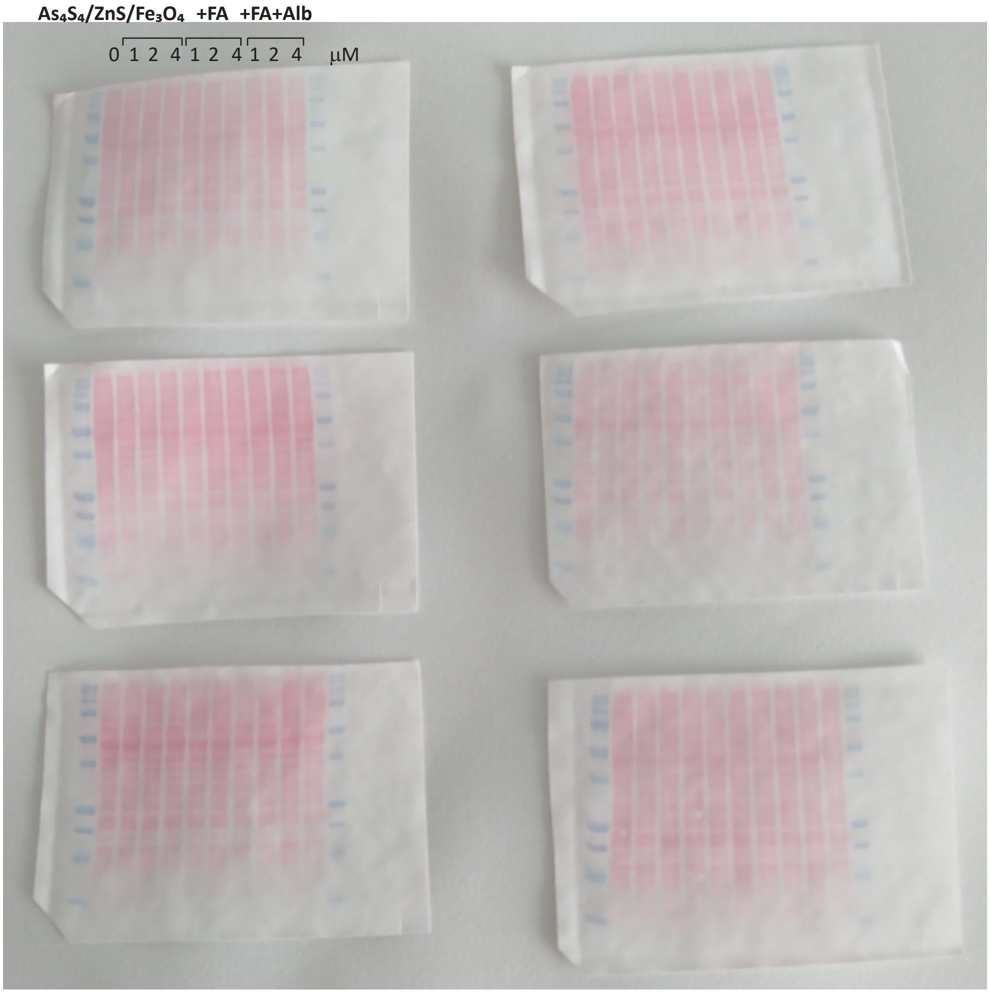

MM.1S 48h

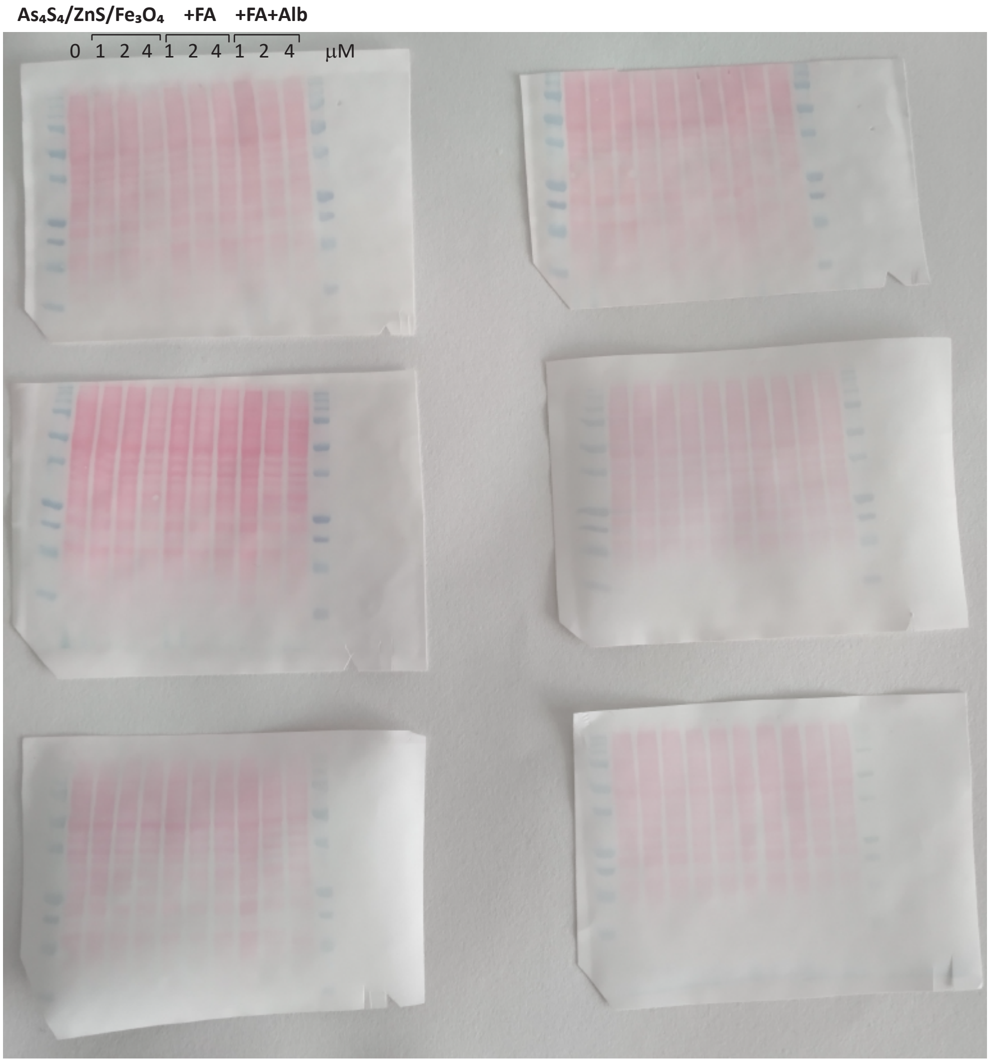

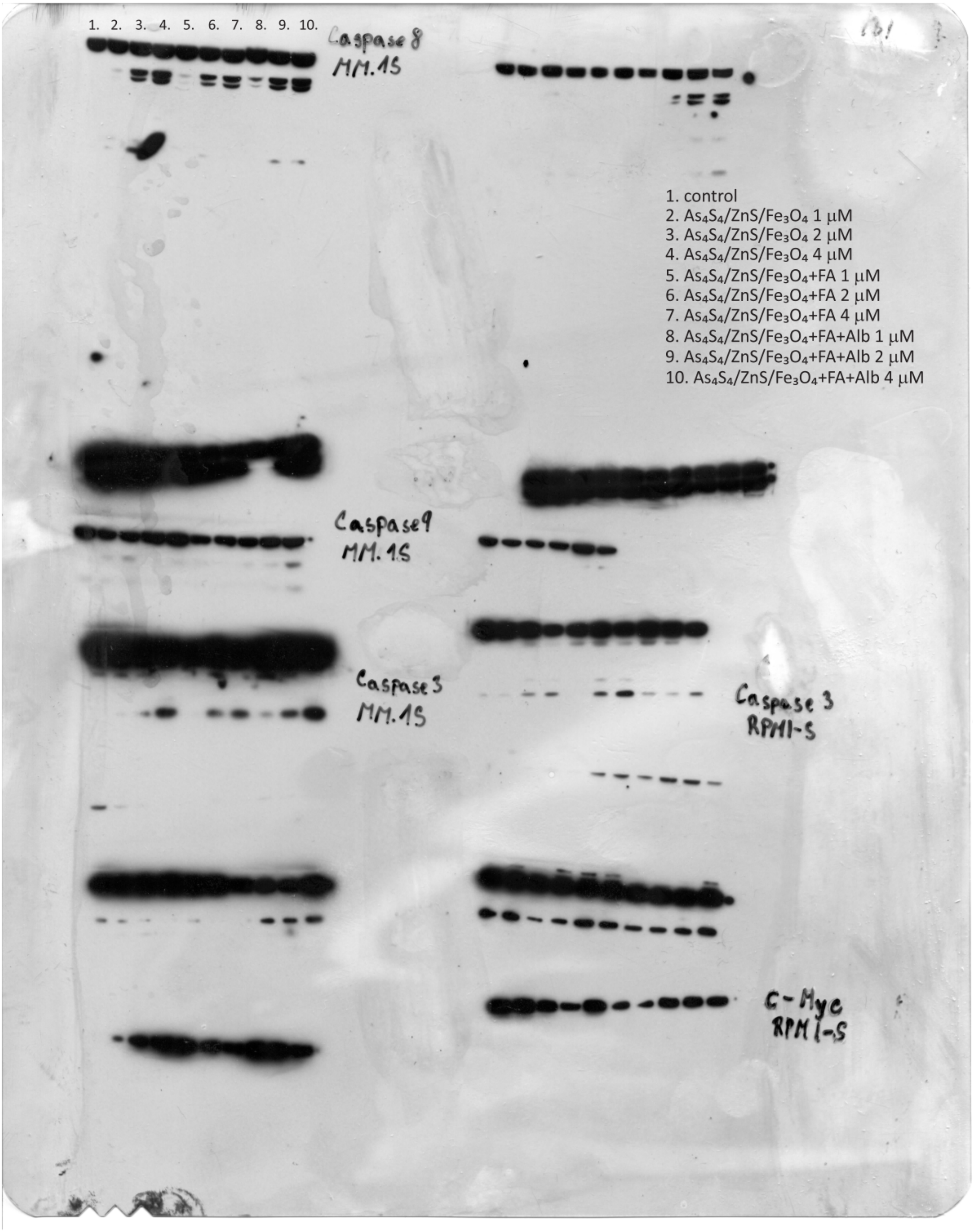
1. 2. 3. 4. 5. 6. 7. 8. 9. 10.
Caspase 8
MM.1S
Caspase9
MM.1S
Caspase3
MM.1S
Caspase 3
RPMI-S
C-Myc
RPMI-S
1. control
2. $As_4S_4/ZnS/Fe_3O_4$ 1 μM
3. $As_4S_4/ZnS/Fe_3O_4$ 2 μM
4. $As_4S_4/ZnS/Fe_3O_4$ 4 μM
5. $As_4S_4/ZnS/Fe_3O_4$+FA 1 μM
6. $As_4S_4/ZnS/Fe_3O_4$+FA 2 μM
7. $As_4S_4/ZnS/Fe_3O_4$+FA 4 μM
8. $As_4S_4/ZnS/Fe_3O_4$+FA+Alb 1 μM
9. $As_4S_4/ZnS/Fe_3O_4$+FA+Alb 2 μM
10. $As_4S_4/ZnS/Fe_3O_4$+FA+Alb 4 μM

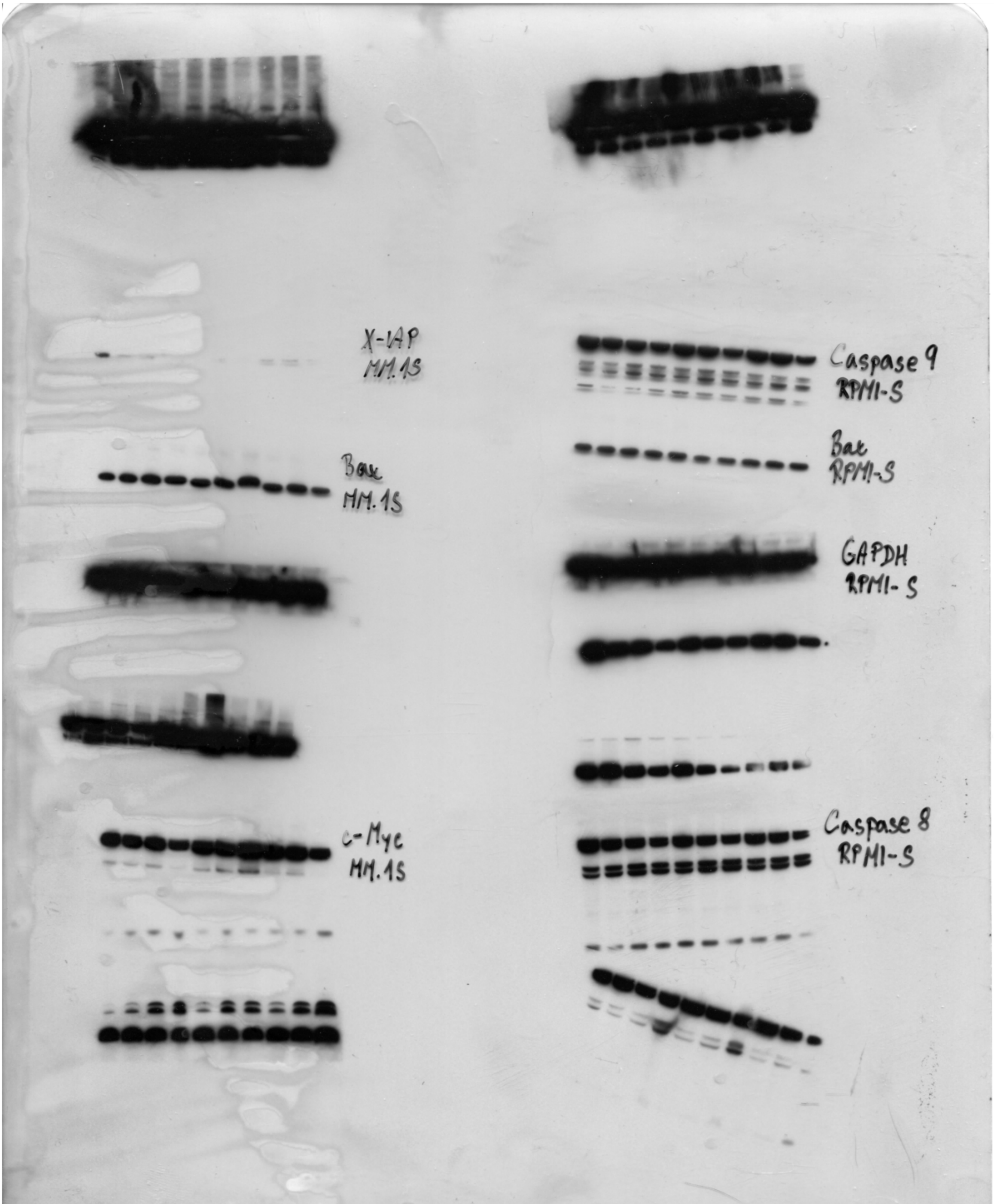
X-IAP
MM.1S
Bax
MM.1S
c-Myc
MM.1S
Caspase 9
RPMI-S
Bax
RPMI-S
GAPDH
RPMI-S
Caspase 8
RPMI-S

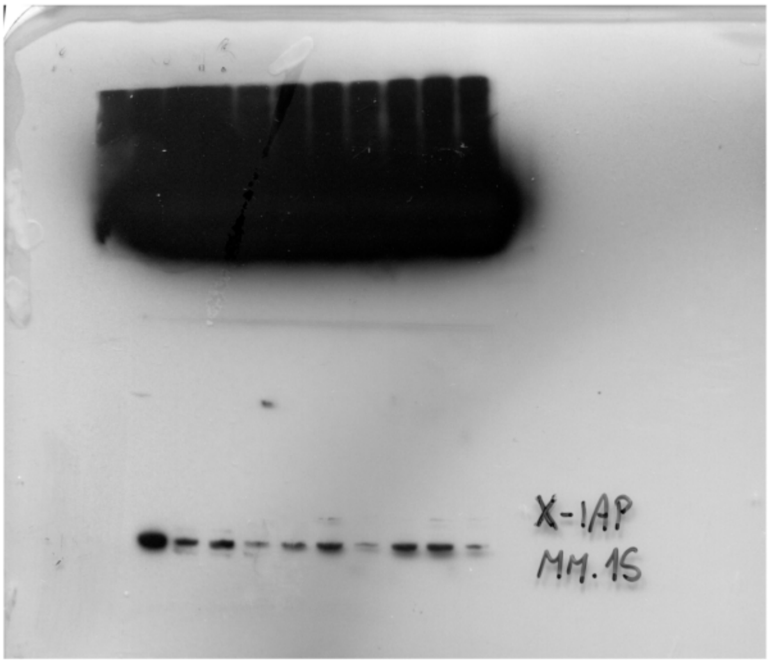
X-IAP
MM.1S

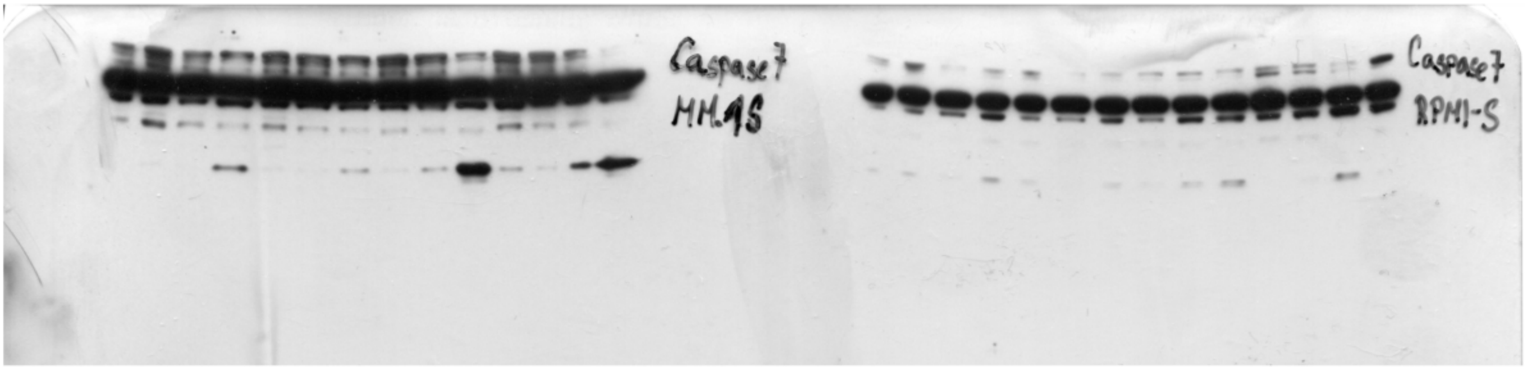
Caspase 7
MM.1S
Caspase 7
RPMI-S

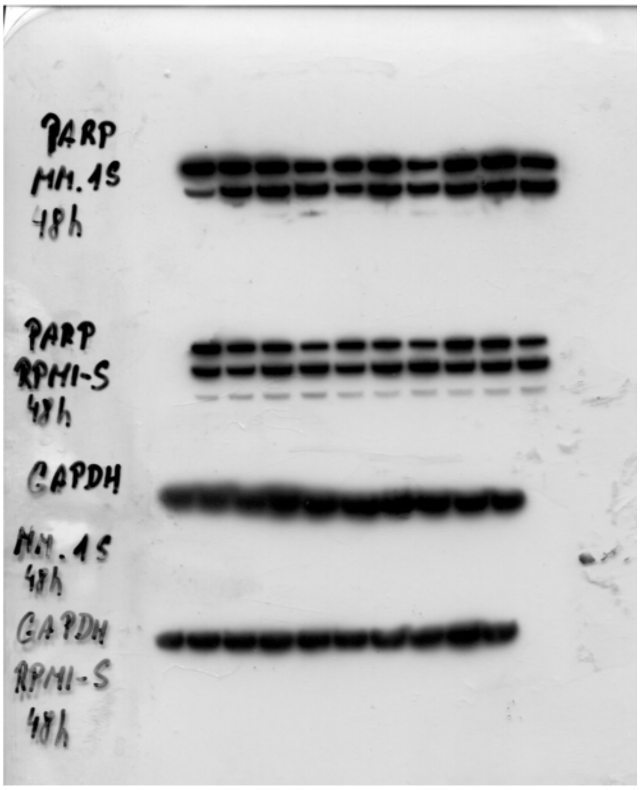
PARP
MM.1S
48h
PARP
RPMI-S
48h
GAPDH
MM.1S
48h
GAPDH
RPMI-S
48h

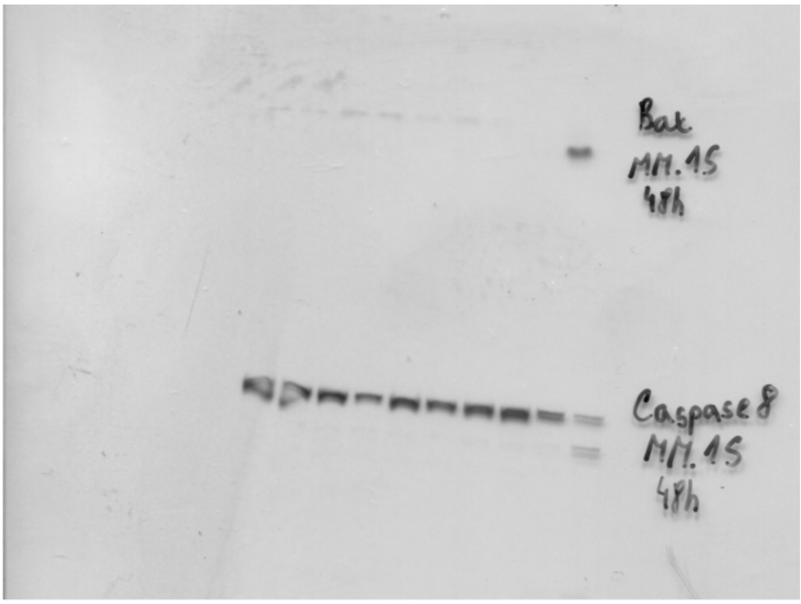
Bax
MM.1S
48h
Caspase 8
MM.1S
48h

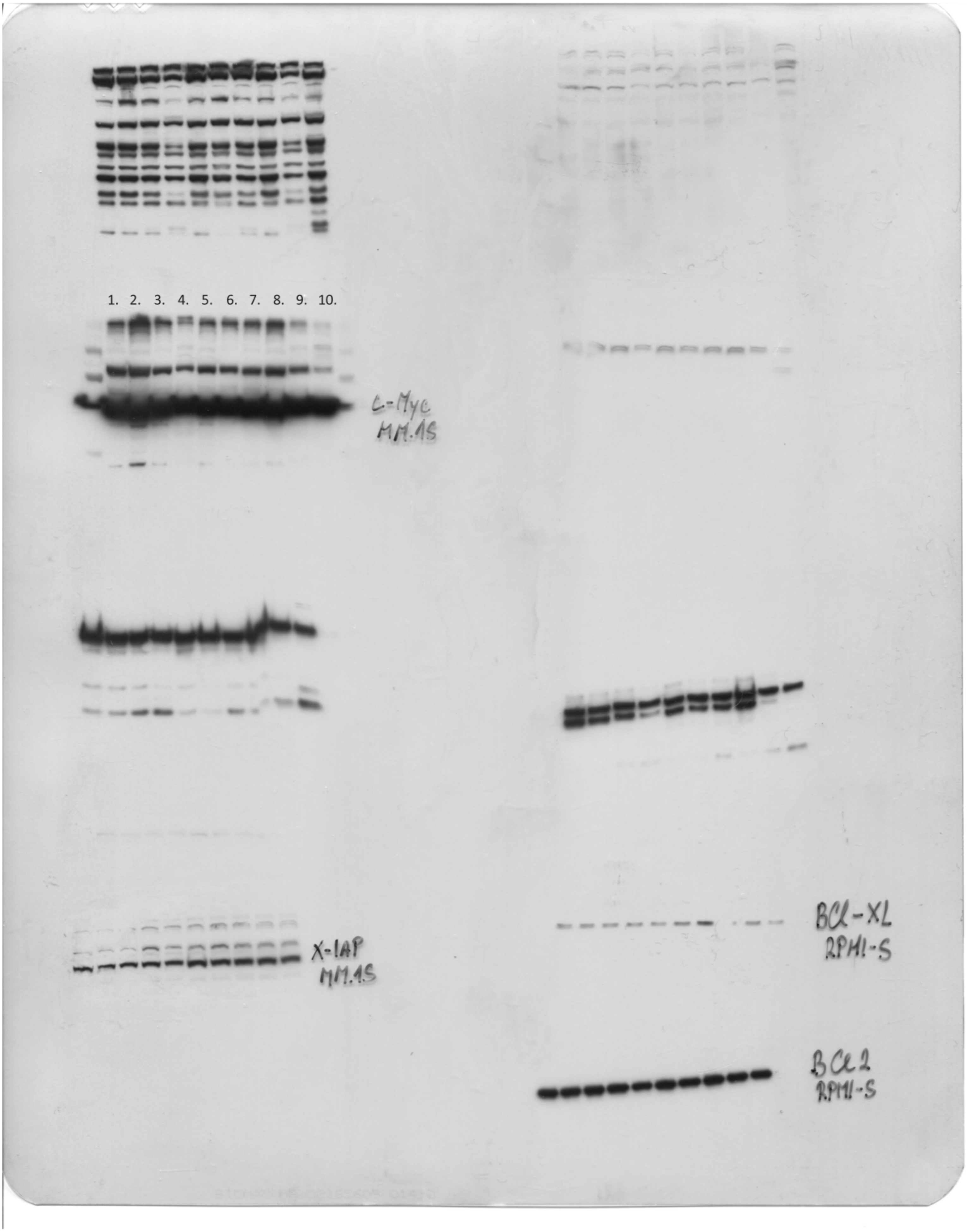
1. 2. 3. 4. 5. 6. 7. 8. 9. 10.
C-Myc
MM.1S
X-IAP
MM.1S
BCl-XL
RPMI-S
BCl 2
RPMI-S

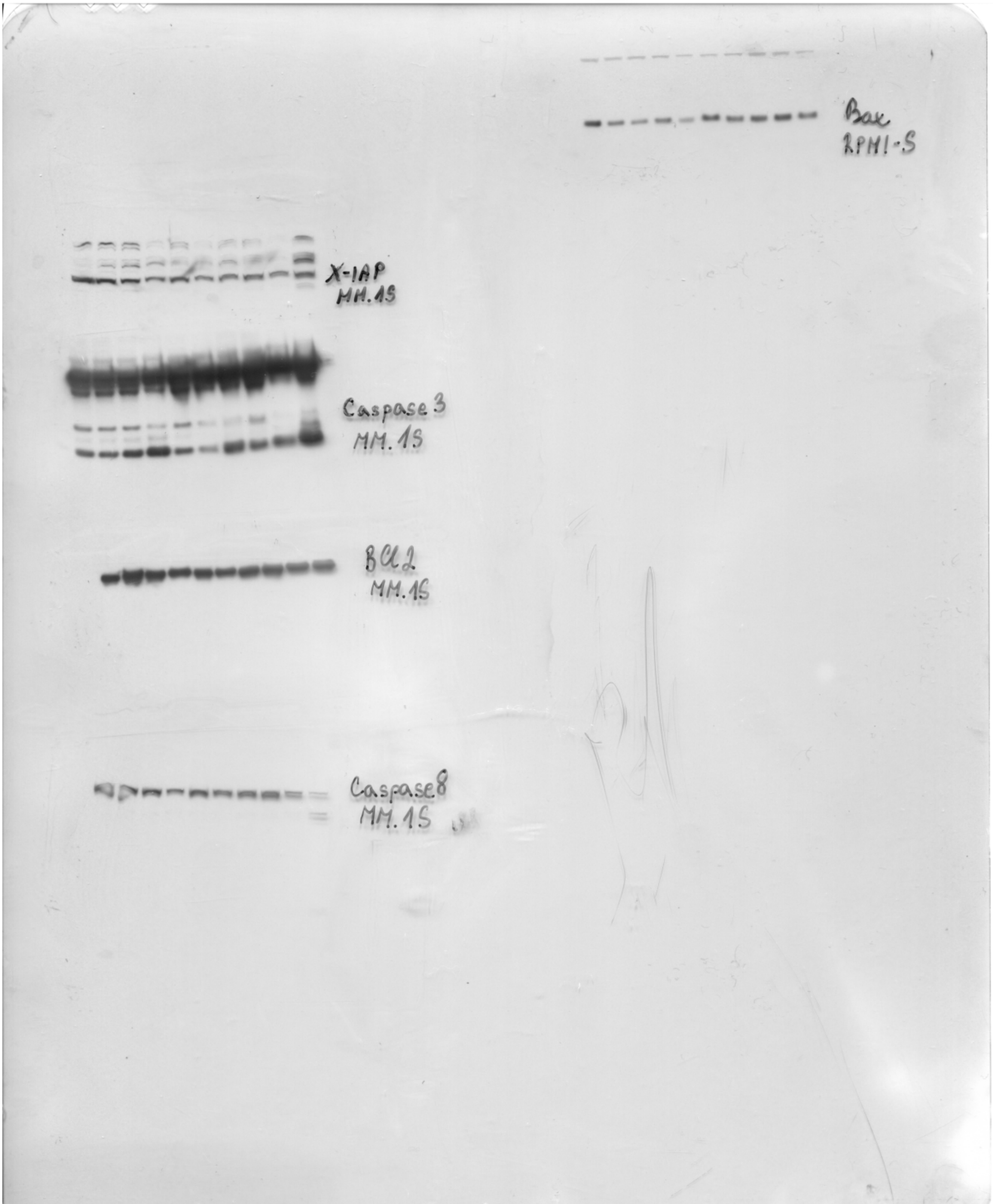
Bax
RPMI-S
X-IAP
MM.1S
Caspase 3
MM.1S
BCl2
MM.1S
Caspase8
MM.1S

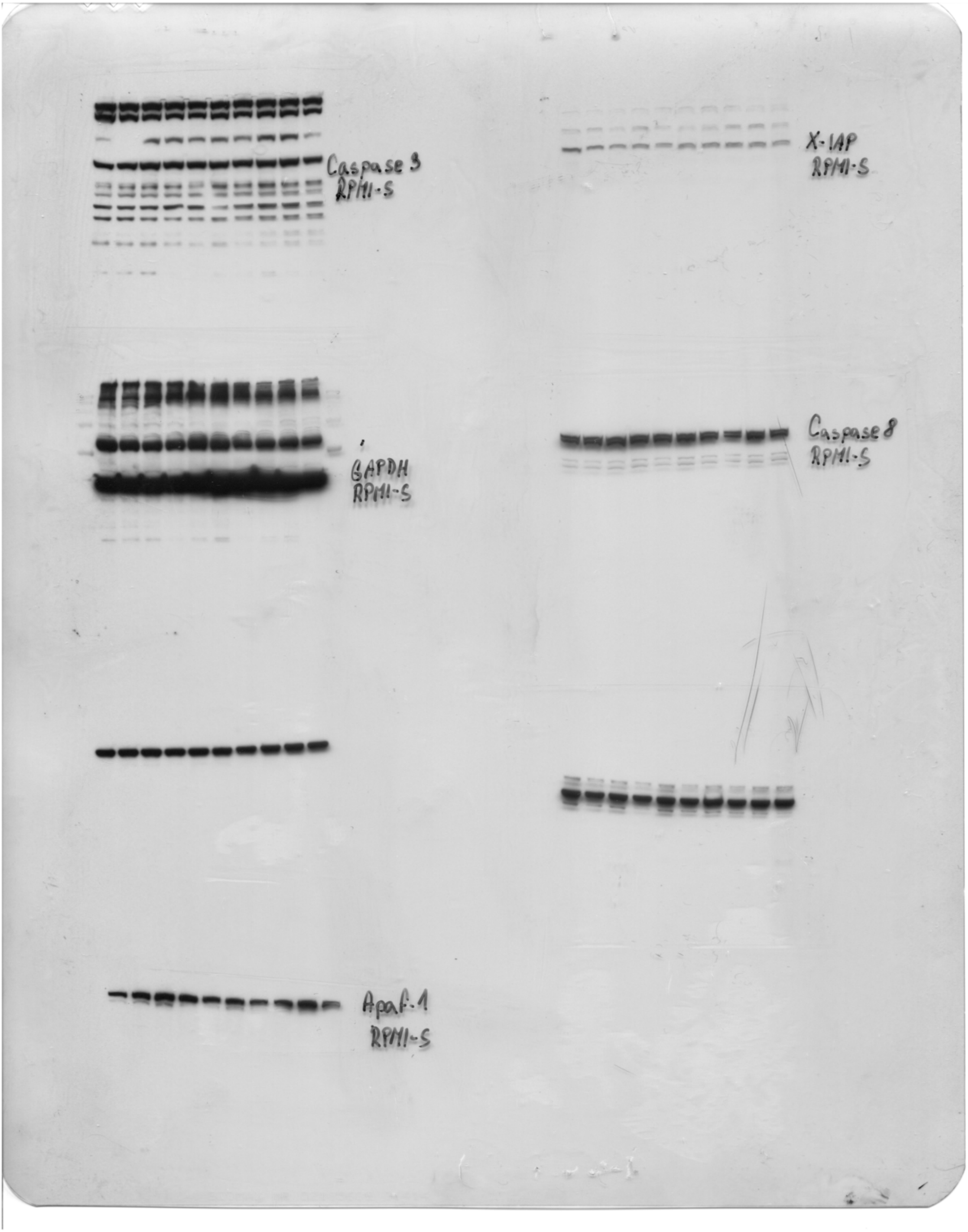

Caspase 3
RPMI-S
X-IAP
RPMI-S
GAPDH
RPMI-S
Caspase 8
RPMI-S
Apaf-1
RPMI-S

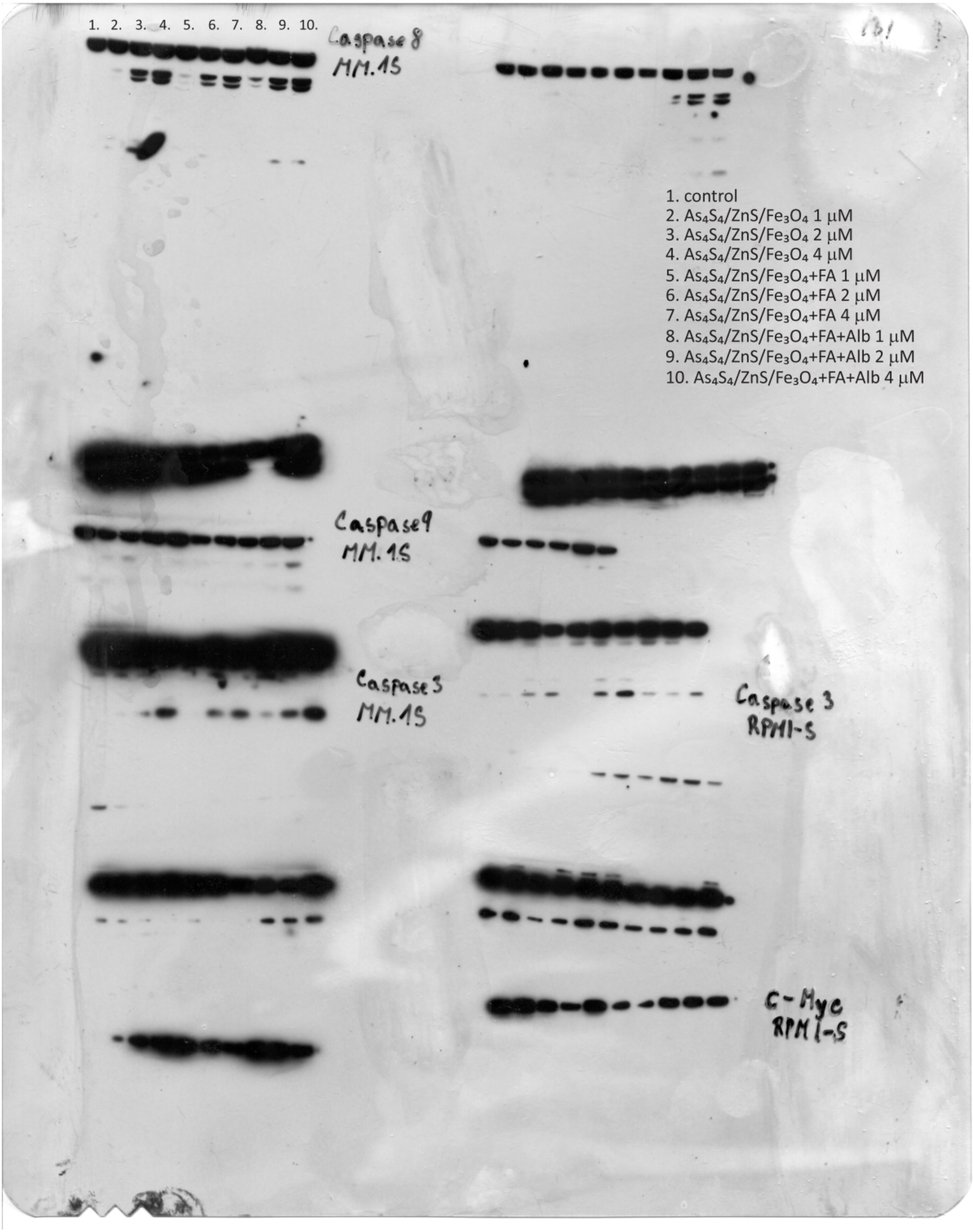
1. 2. 3. 4. 5. 6. 7. 8. 9. 10.
Caspase 8
MM.1S
Caspase 9
MM.1S
Caspase 3
MM.1S
Caspase 3
RPMI-S
C-Myc
RPMI-S
1. control
2. $As_4S_4/ZnS/Fe_3O_4$ 1 μM
3. $As_4S_4/ZnS/Fe_3O_4$ 2 μM
4. $As_4S_4/ZnS/Fe_3O_4$ 4 μM
5. $As_4S_4/ZnS/Fe_3O_4$+FA 1 μM
6. $As_4S_4/ZnS/Fe_3O_4$+FA 2 μM
7. $As_4S_4/ZnS/Fe_3O_4$+FA 4 μM
8. $As_4S_4/ZnS/Fe_3O_4$+FA+Alb 1 μM
9. $As_4S_4/ZnS/Fe_3O_4$+FA+Alb 2 μM
10. $As_4S_4/ZnS/Fe_3O_4$+FA+Alb 4 μM
