# Supplementary material for: In vitro and ex vivo anti-myeloma effects of nanocomposite As4S4/ZnS/Fe3O4
Source: Sci Rep. 2022 Oct 26;12:17961. doi: 10.1038/s41598-022-22672-5 (PMC9606304; doi:10.1038/s41598-022-22672-5)
Supplement: Supplementary file 11 — Supplementary Information 11. [file 41598_2022_22672_MOESM11_ESM.pdf]

RPMI-S 8h **MM.1S 8h**

$As_4S_4/ZnS/Fe_3O_4$ +FA +FA+Alb

$As_4S_4/ZnS/Fe_3O_4$ +FA +FA+Alb

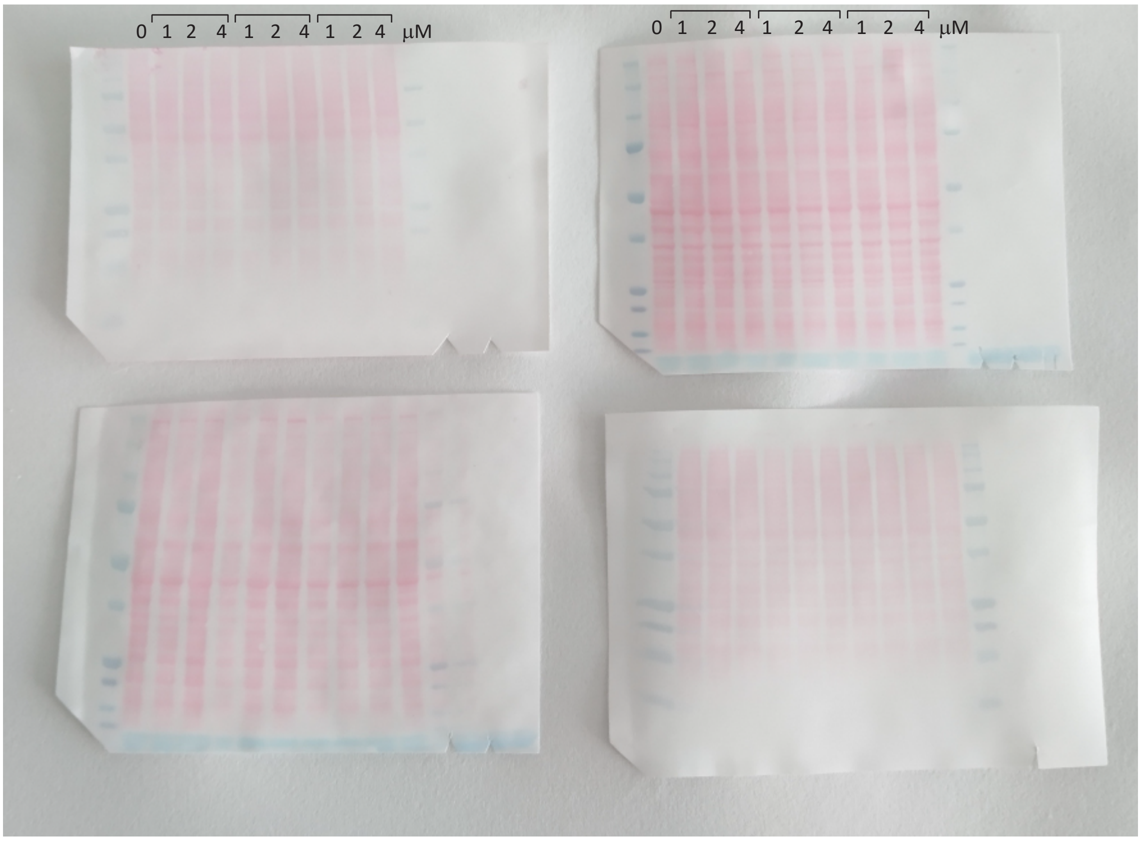

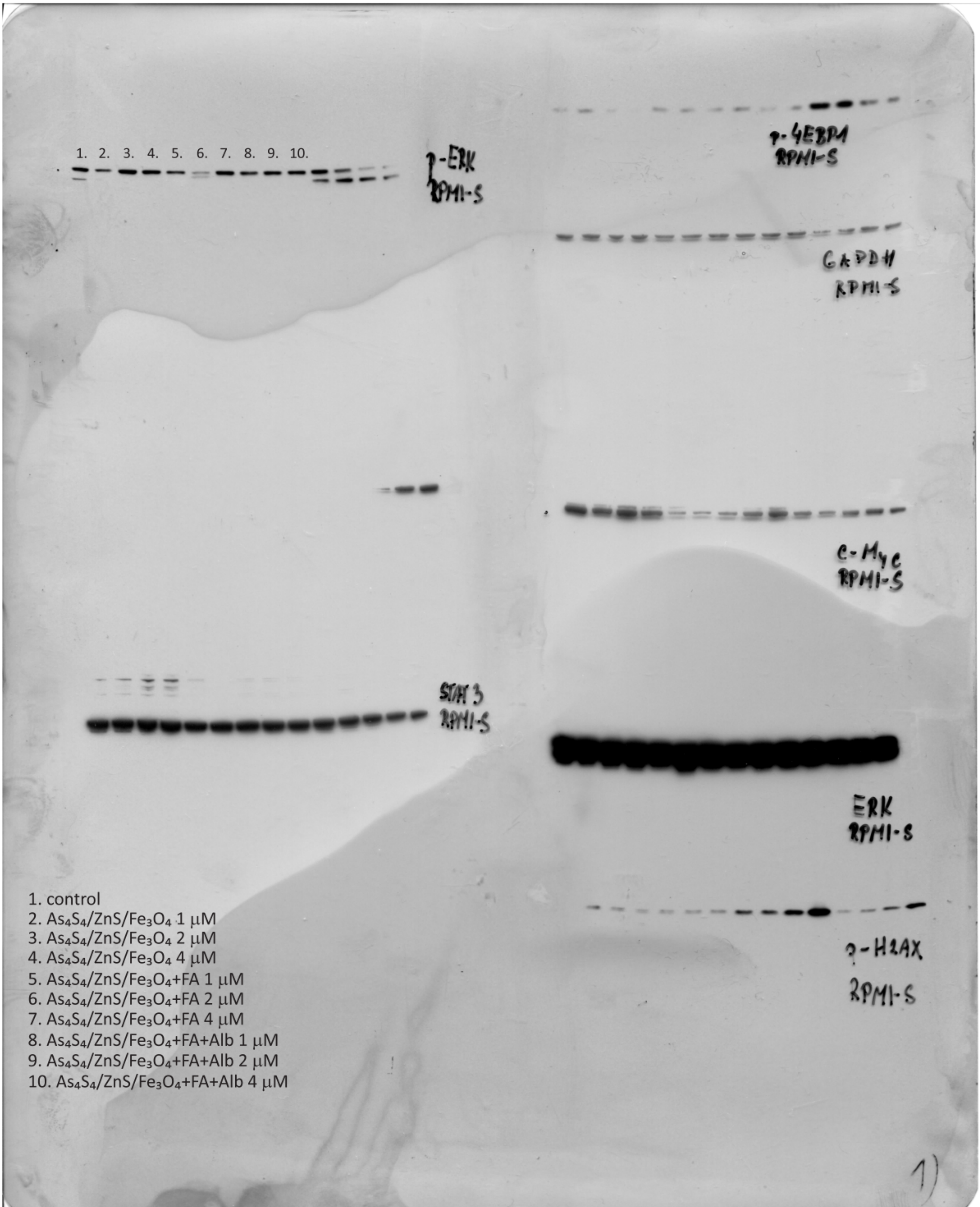

1. 2. 3. 4. 5. 6. 7. 8. 9. 10.
p-ERK RPMI-S
p-4EBP1 RPMI-S
GAPDH RPMI-S
c-Myc RPMI-S
STAT 3 RPMI-S
ERK RPMI-S
γ-H2AX RPMI-S
1. control
2. $As_4S_4/ZnS/Fe_3O_4$ 1 μM
3. $As_4S_4/ZnS/Fe_3O_4$ 2 μM
4. $As_4S_4/ZnS/Fe_3O_4$ 4 μM
5. $As_4S_4/ZnS/Fe_3O_4$+FA 1 μM
6. $As_4S_4/ZnS/Fe_3O_4$+FA 2 μM
7. $As_4S_4/ZnS/Fe_3O_4$+FA 4 μM
8. $As_4S_4/ZnS/Fe_3O_4$+FA+Alb 1 μM
9. $As_4S_4/ZnS/Fe_3O_4$+FA+Alb 2 μM
10. $As_4S_4/ZnS/Fe_3O_4$+FA+Alb 4 μM
1)

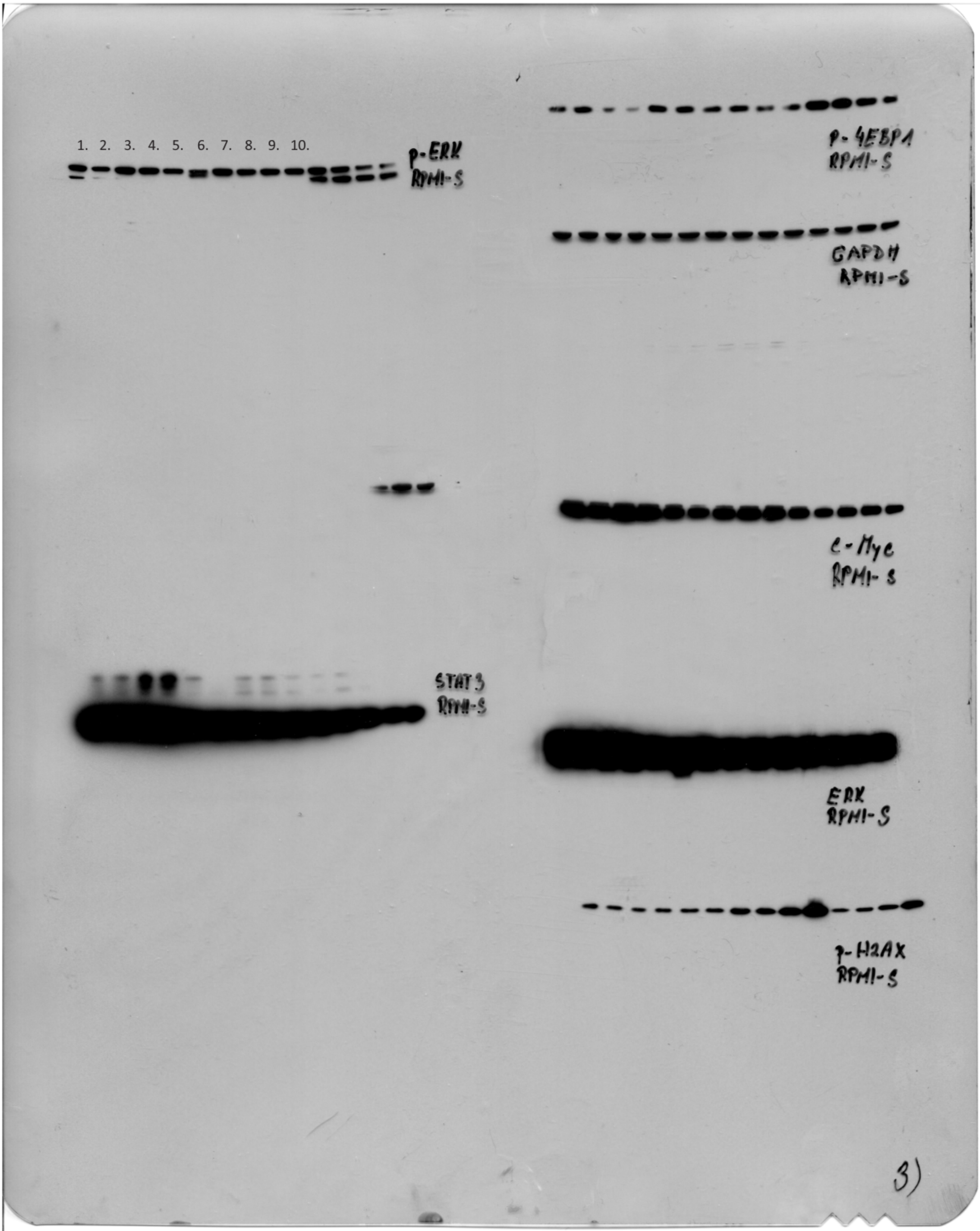
1. 2. 3. 4. 5. 6. 7. 8. 9. 10.
p-ERK RPMI-S
p-4EBP1 RPMI-S
GAPDH RPMI-S
c-Myc RPMI-S
STAT3 RPMI-S
ERK RPMI-S
p-H2AX RPMI-S
3)

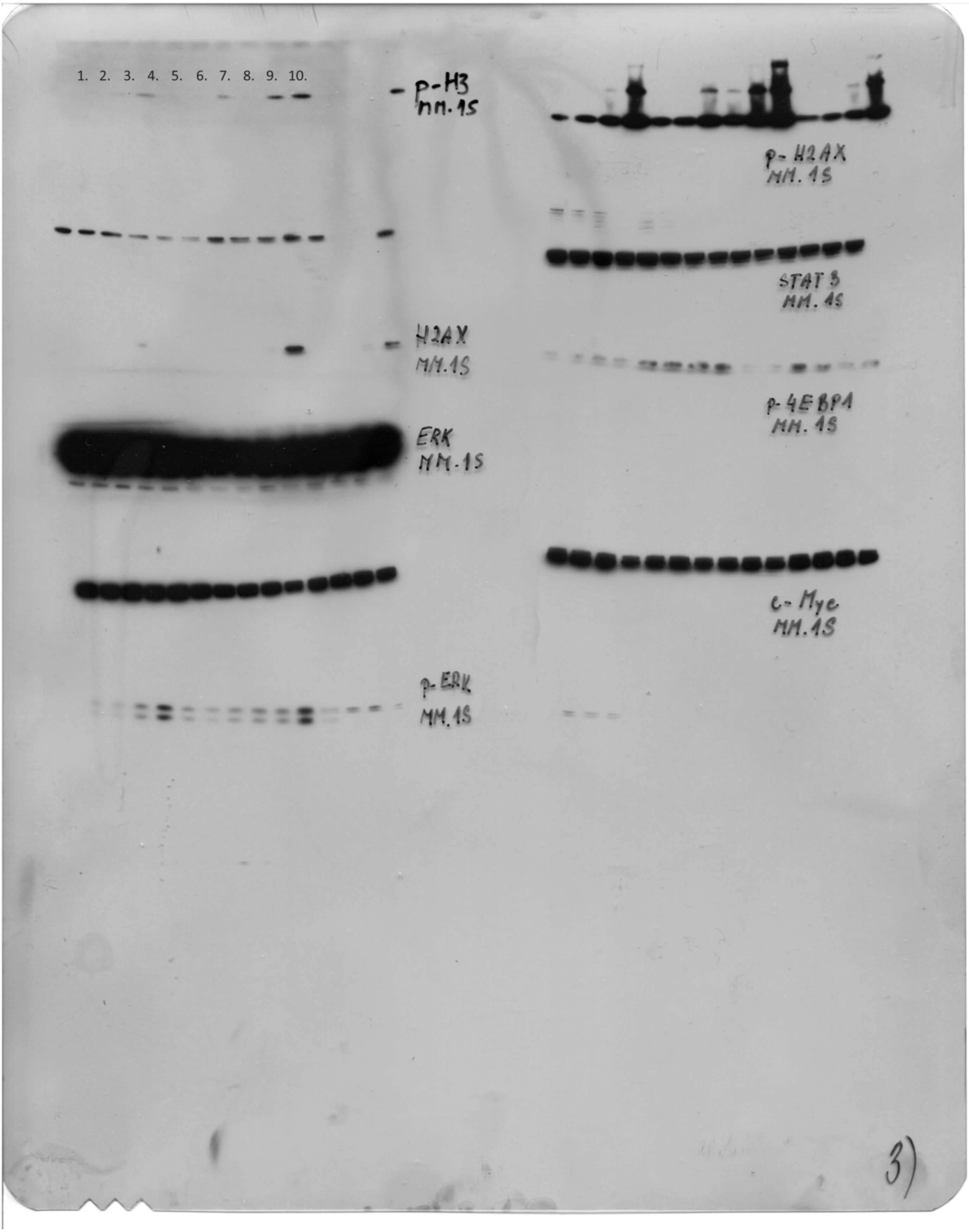
1. 2. 3. 4. 5. 6. 7. 8. 9. 10.
p-H3 MM.1S
H2AX MM.1S
ERK MM.1S
p-ERK MM.1S
p-H2AX MM.1S
STAT 3 MM.1S
p-4EBP1 MM.1S
c-Myc MM.1S
3)

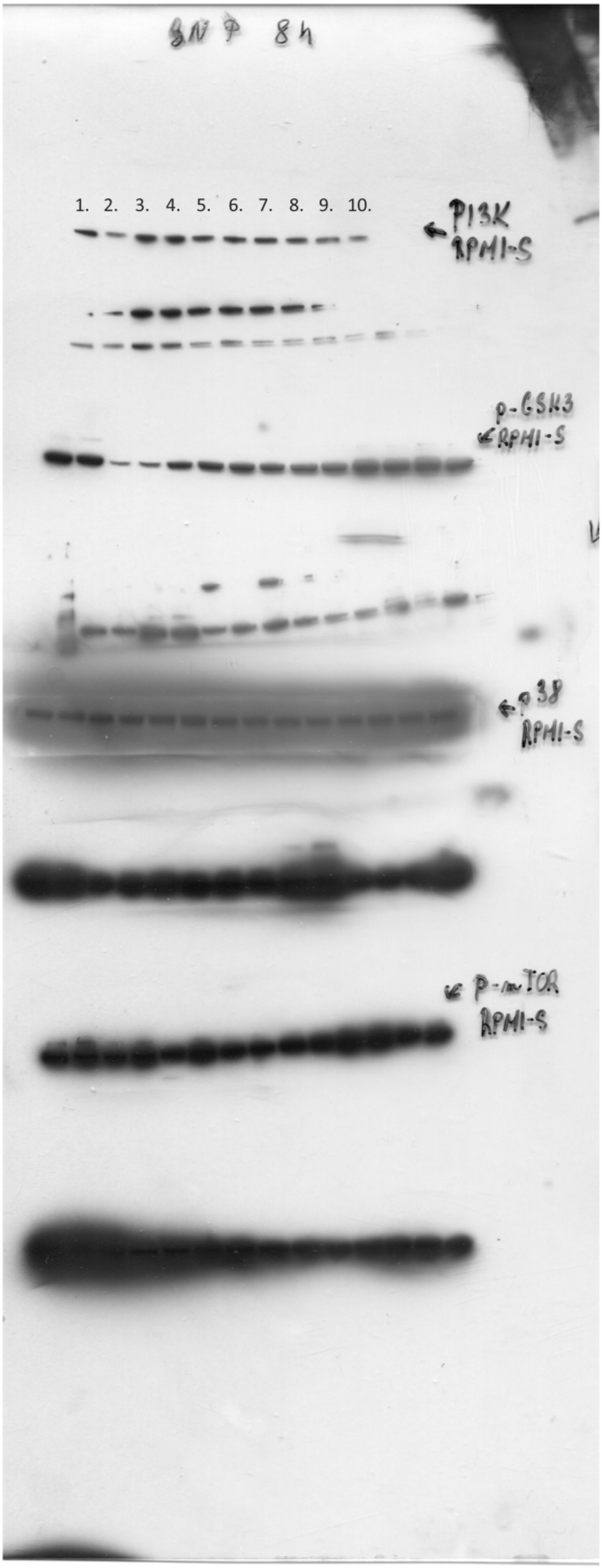
BNP 8h
1. 2. 3. 4. 5. 6. 7. 8. 9. 10.
PI3K RPMI-S
p-GSK3 RPMI-S
p38 RPMI-S
p-mTOR RPMI-S

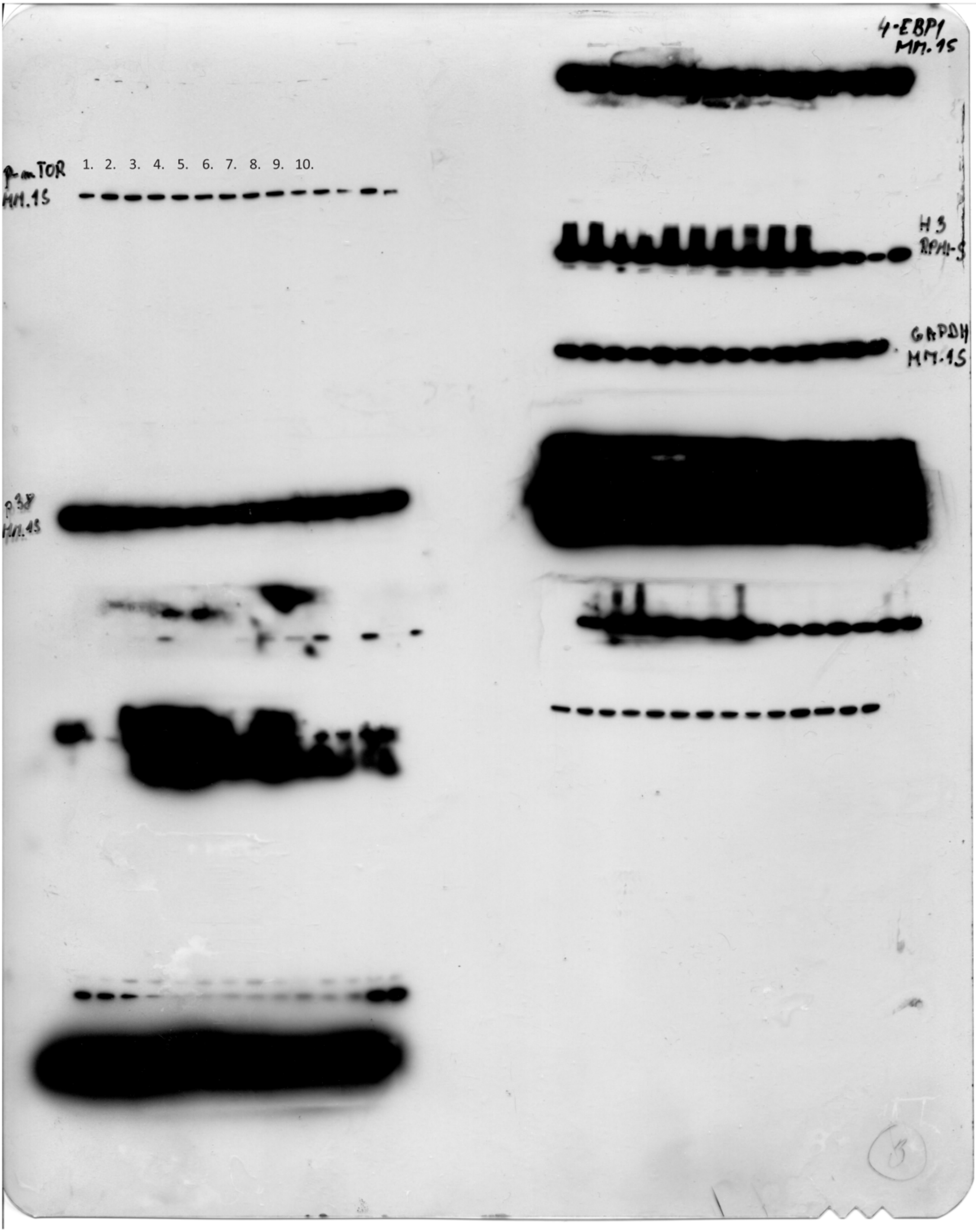

4-EBP1
MM.1S
p-mTOR
MM.1S
1. 2. 3. 4. 5. 6. 7. 8. 9. 10.
H3
RPMI-S
GAPDH
MM.1S
p38
MM.1S

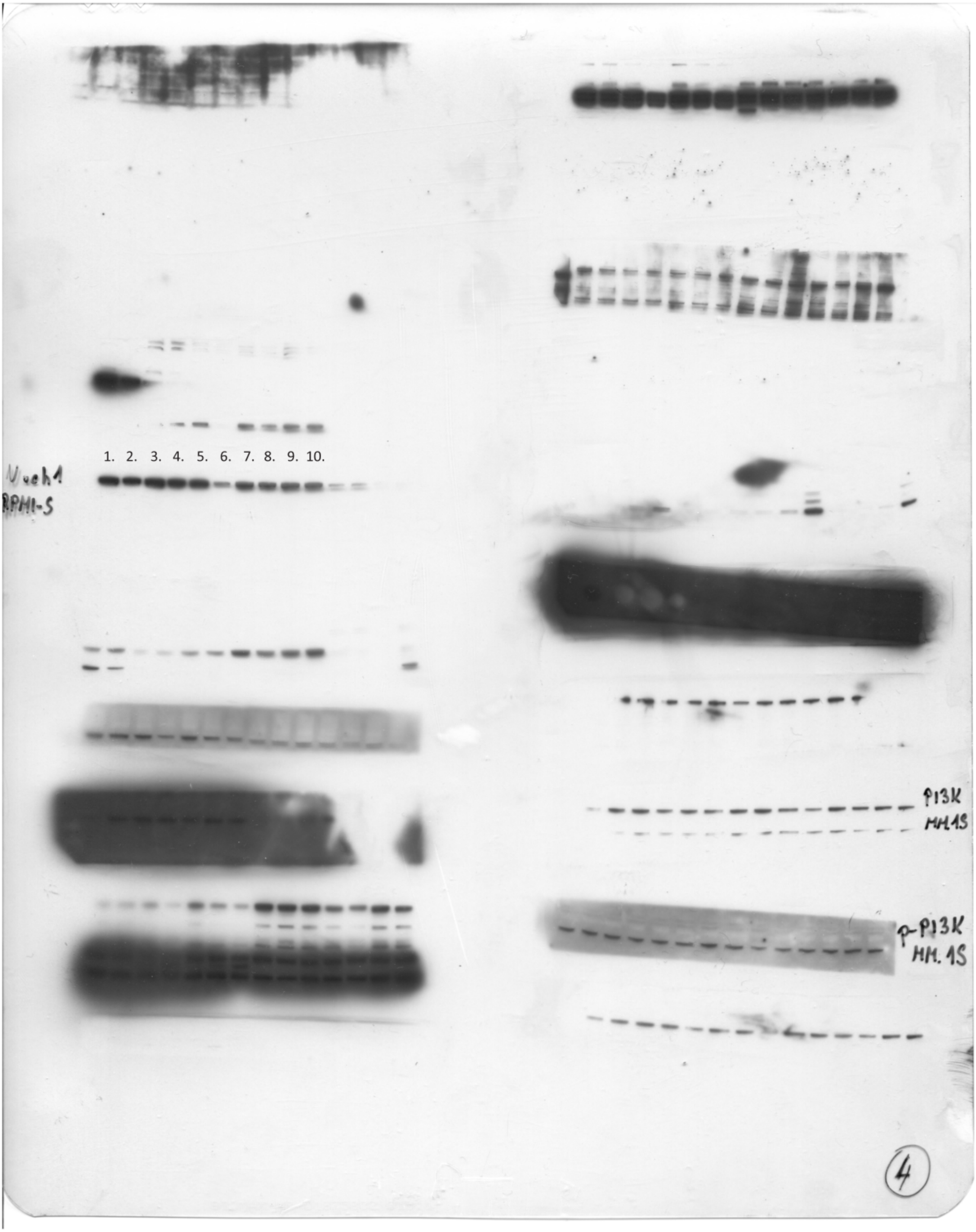
1. 2. 3. 4. 5. 6. 7. 8. 9. 10.
Nuch1
RPMI-S
PI3K
MM.1S
p-PI3K
MM. 1S
4

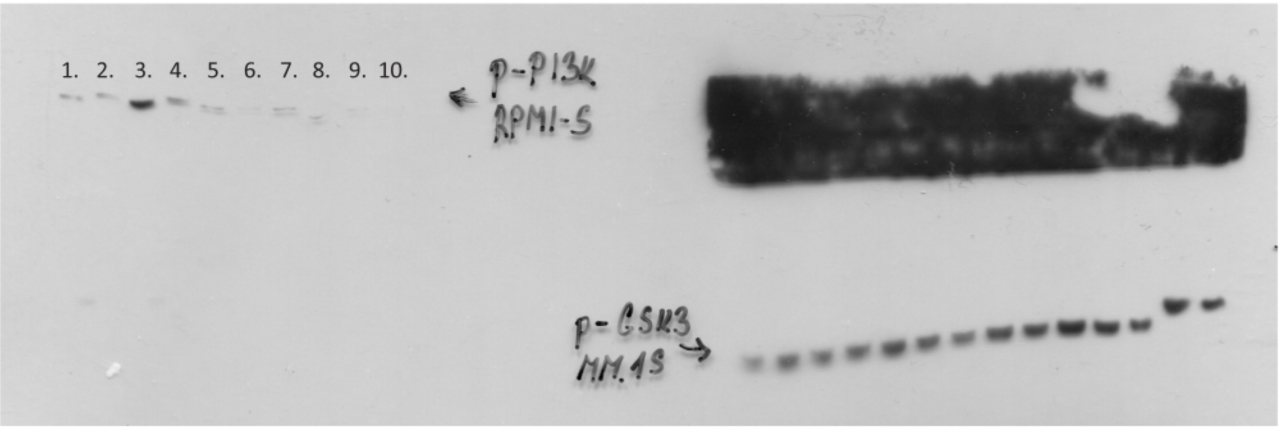
1. 2. 3. 4. 5. 6. 7. 8. 9. 10.
p-PI3K
RPMI-S
p-GSK3
MM.1S

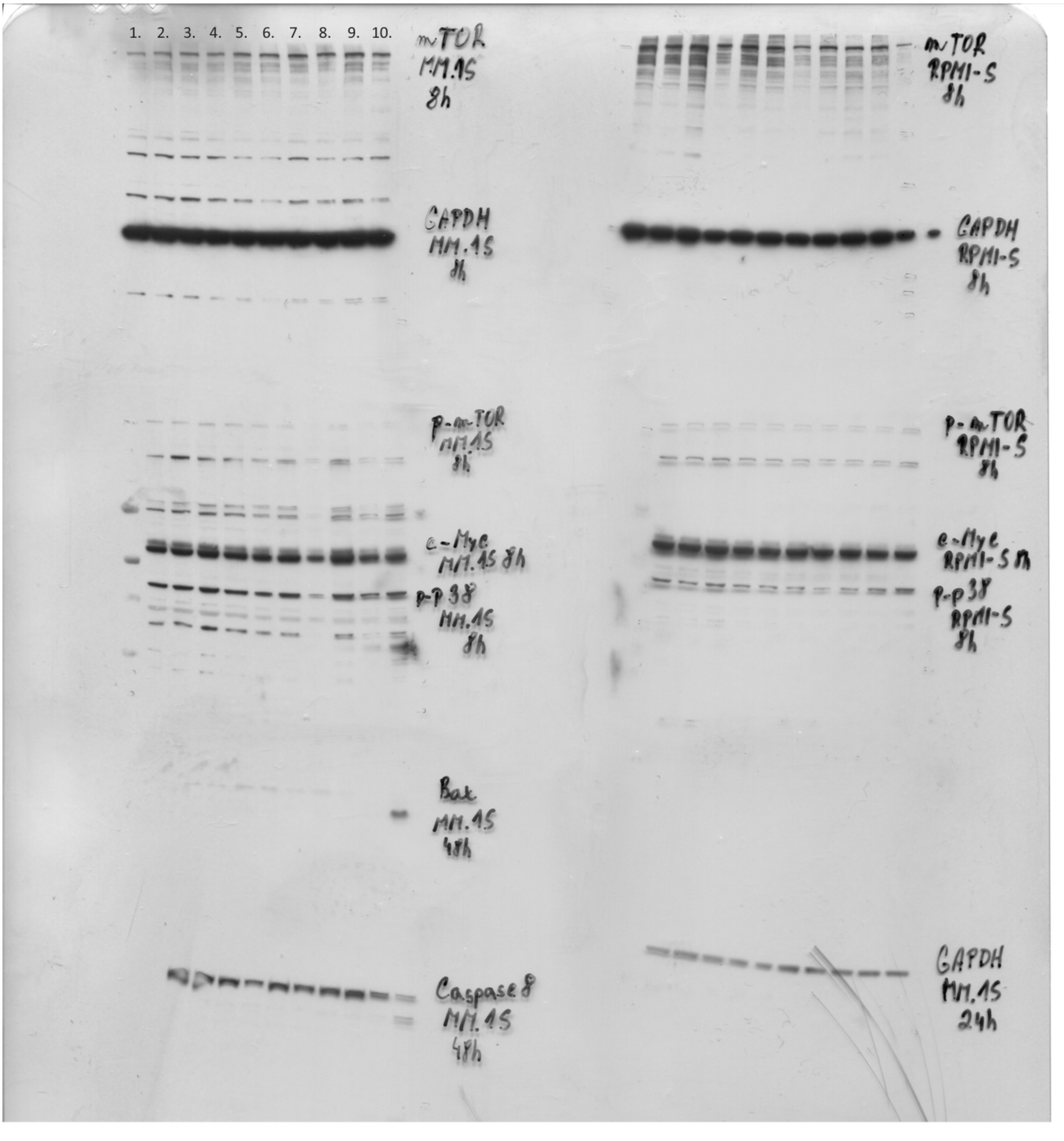
1. 2. 3. 4. 5. 6. 7. 8. 9. 10.
mTOR
MM.1S
8h
mTOR
RPMI-S
8h
GAPDH
MM.1S
8h
GAPDH
RPMI-S
8h
p-mTOR
MM.1S
8h
p-mTOR
RPMI-S
8h
c-Myc
MM.1S 8h
c-Myc
RPMI-S 8h
p-p38
MM.1S
8h
p-p38
RPMI-S
8h
Bax
MM.1S
48h
Caspase 8
MM.1S
48h
GAPDH
MM.1S
24h
